# Enhancing Activation of D_2_O for Highly Efficient Deuteration Using an Fe–P Pair-Site Catalyst

**DOI:** 10.1021/jacsau.5c00257

**Published:** 2025-05-21

**Authors:** Haifeng Qi, Yueyue Jiao, Qiang Wang, Nicholas F. Dummer, Jianglin Duan, Yujing Ren, Stuart H. Taylor, Haijun Jiao, Kathrin Junge, Graham J. Hutchings, Matthias Beller

**Affiliations:** † 28392 Leibniz-Institut für Katalyse e. V., Albert-Einstein-Straβe 29a, Rostock 18059, Germany; ‡ Max Planck-Cardiff Centre on the Fundamentals of Heterogeneous Catalysis FUNCAT, Translational Research Hub, 2112 Cardiff University, Maindy Road, Cardiff CF24 4HQ, U.K.; § SINOPEC Research Institute of Petroleum Processing Co., Ltd., Beijing 100083, P. R. China; ∥ Interdisciplinary Research Center of Biology & Catalysis, School of Life Sciences, 26487 Northwestern Polytechnical University, Xi’an 710072, China

**Keywords:** iron, single-atom catalyst, deuteration, D_2_O activation, catalytic pair

## Abstract

Deuterated amine derivatives have emerged as valuable compounds in medicinal chemistry and materials science due to their enhanced metabolic stability and unique physicochemical properties, emphasizing the need for cost-effective and efficient deuteration catalysts; yet this topic has rarely been explored. In this work, we present an atomically dispersed Fe–P pair-site catalyst with high catalytic efficiency and regioselectivity in the deuteration of arenes and heteroarenes using D_2_O as the deuterated source. Remarkably, these metal–nonmetal Fe–P catalytic pairs with low Fe loading (0.15 wt %) achieve superior catalytic efficiency with a turnover frequency of 131.3 h^–1^, demonstrating activity up to 30 times higher than the state-of-the-art Fe nanoparticle catalyst (4.9 wt %, TOF: 4.5 h^–1^). Mechanistic investigations and density functional theory reveal that Fe–P pair sites play a key role in activating D_2_O and the substrate, enabling the regioselective deuteration of (hetero)­arenes. The investigation further demonstrates the remarkable performance of the phosphorus-doped Fe single-atom catalyst (SAC) across a diverse array of substrates, including various functional group-substituted anilines, nitrogen-containing heterocycles, phenol derivatives, and even complex drug molecules, yielding a total of 39 deuterated compounds. The scale-up synthesis of the Fe–P–C catalyst and subsequent stability tests further underscore the catalyst’s potential for practical applications. This methodology introduces a promising direction for developing low-cost, non-noble metal SACs, offering significant potential for advancing the sustainable synthesis of fine chemicals.

## Graphical abstract

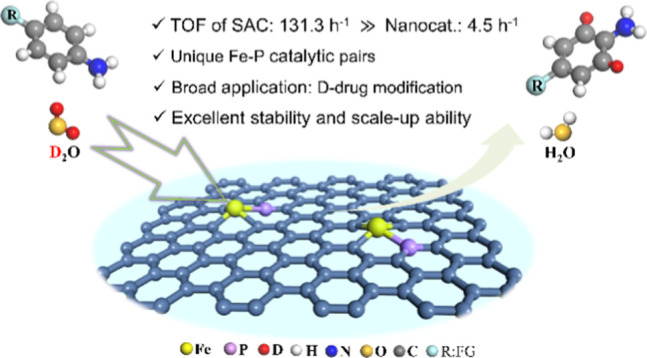

## Introduction

Hydrogen isotope deuterium (D)-labeled compounds play a crucial role in the development of new pharmaceuticals and materials.^1,2^ The incorporation of isotopes offers an opportunity to enhance drug safety by producing metabolites with decreased toxicity, improving drug tolerability, and increasing drug bioavailability compared to nondeuterated analogues.^3,4^ Consequently, deuterium-labeled compounds have attracted considerable interest as potential therapeutic agents; for instance, the U.S. Food and Drug Administration-approved Austedo (deutetrabenazine), the first deuterated drug, for the treatment of movement disorders associated with Huntington’s disease.^3–6^ Additionally, several other deuterium-modified drugs, such as CTP-543 (Ruxolitinib), CTP-656 (Kalydeco), DRX-065 (Pioglitazone), and BMS-986165 (Rosuvastatin), developed by companies such as Concert, Vertex, DeuteRx, and BMS, have already been in clinical trials.^5^ This demonstrates the potential of deuterated pharmaceuticals. Consequently, a wide range of synthetic methods has been developed to enable the preparation of deuterium-labeled compounds with high deuterium incorporation. Among these methodologies, hydrogen isotope exchange (HIE) has gained prominence due to its ability to enable the selective preparation of deuterated compounds at specific positions.^7,8^ As an example, Lei, Li, and co-workers have recently made a notable contribution to this area by developing a general electrocatalytic method that enables the reductive deuteration and deuterodefluorination of (hetero)­arenes.^9^

Aromatic hydrocarbons are abundant structural motifs in pharmaceuticals and agrochemicals, playing an important role as synthetic materials in numerous therapeutics and bioactive natural products. According to statistical data from the Njardarson group at the University of Arizona, amino-substituted (hetero)­aromatic hydrocarbons comprised over 50% of the top 200 best-selling small molecule drugs in 2023,^10^ highlighting their potential as building blocks in the synthesis of deuterated heterocyclic pharmaceuticals. However, the selective synthesis of deuterium-labeled (hetero)­arenes via HIE owning a precise position with high isotopic purity (≥95%) is more challenging than fully incorporating deuterium atoms of the arene ring. Following the initial development of Raney alloy deuterated catalysts, significant progress has been made in designing both homogeneous and heterogeneous catalytic systems capable of achieving selective deuterium labeling of aniline derivatives (Figure 1). Here, homogeneous catalysts are based on Pd, Ir, Ag, and Ru complexes (Figure 1a) but often suffer from their inherent instability, difficulty in recycling, and the need for large amounts of organic ligands.^11–20^ In addition, heterogeneous catalytic systems, predominantly utilizing noble metals including Pd, Pt, Ru, and Rh, have been explored for such transformations (Figure 1b).^21–23^

**Figure 1:**
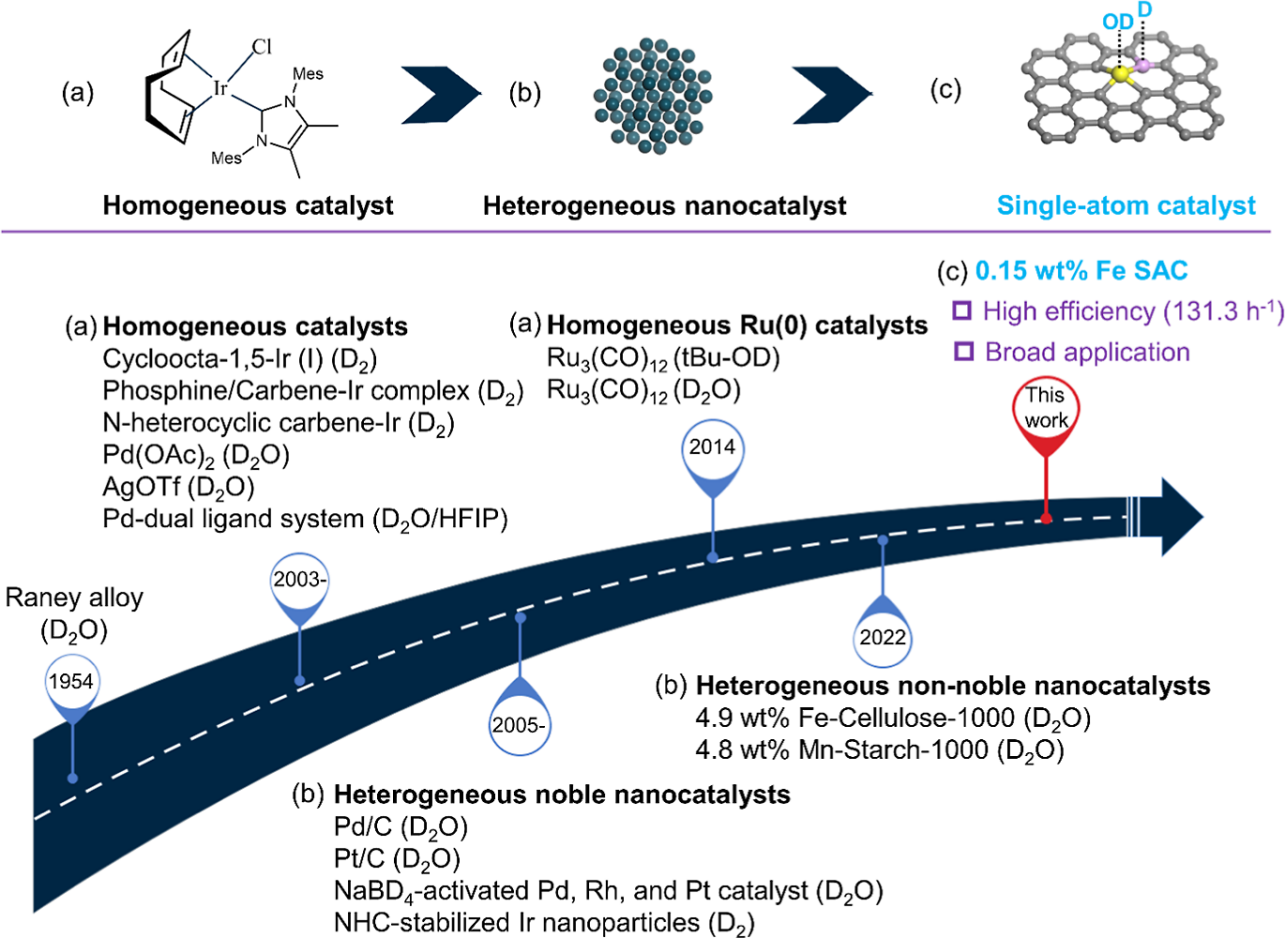
Historical development of selected (a) homogeneous and (b) heterogeneous catalysts for deuterium labeling of (hetero)­arenes, and (c) this work of Fe single-atom catalyst (SAC).

More recently, advanced heterogeneous Fe- and Mn-based nanocatalysts have emerged for the efficient deuteration of (hetero)­arenes (Figure 1b).^24,25^ Nonetheless, the relatively high metal contents required (approximately 20 mol % Fe and 10 mol % Mn), coupled with the structural heterogeneity of metal nanoparticles, complicate the identification of active sites and hinder the development of more efficient deuteration catalysts. In this regard, SACs, characterized by atomically dispersed metal species anchored on solid supports with well-defined mononuclear active sites, offer a promising approach that could combine the benefits of homogeneous catalysts with those of heterogeneous catalysts.^26–28^ We envision that through the rational design of SACs, it will be possible to achieve highly efficient deuteration performance in this critical area.^29^

In this work, we present the utilization of atomically dispersed Fe–P catalytic pairs, supported on phosphorus-doped carbon, to achieve the selective deuteration of (hetero)­arenes. This process utilizes inexpensive D_2_O as the deuterium source, as illustrated in Figure 1c. The Fe–P–C SAC (0.16 mol % Fe usage), a recently developed catalyst,^29^ exhibits remarkable performance and stability in the deuteration of a broad range of anilines, heterocycles, phenol derivatives, and drug molecules.

## Results and Discussions

### Benchmark Reaction: Catalytic Performance

Inspired by previous works suggesting that an M–N site as a frustrated Lewis acid–base pair could effectively catalyze the activation of H_2_O to form a Brønsted acid–base pair (H^δ+^–N–M–OH^δ−^),^30,31^ we began synthesizing the different nitrogen (N)-doped carbon supported Fe SACs, along with a series of Fe-based materials coordinated with sulfur (S) or phosphorus (P). For instance, the P-doped carbon-supported Fe catalysts were synthesized via high-temperature pyrolysis of a mixture comprising phytic acid and Fe­(NO_3_)_3_·9H_2_O under an argon atmosphere; the detailed synthetic process can be found in the Supporting Information.^29,32,33^

As a model system, we investigated the deuterated labeling of *p*-anisidine **1a**, a raw material for producing Apixaban, in D_2_O using the prepared catalysts (Table 1 and Table S1). Initially, Fe–N–C materials exhibited minimal deuteration activity (entry 1, Table 1 and Table S1). Then, we examined the impact of the coordination environment surrounding the dispersed metal centers on their catalytic performance. Specifically, we evaluated the catalytic performance of S- and P-doped carbon-supported Fe-based materials in the model reaction. Over the Fe–S–C materials, with sulfur-doped carbon, an improved deuterium incorporation (D) was already observed (∼67% D, entry 2, Table 1 and Table S1). The Fe–P–C, featuring a P-doped carbon support, showed a significant increase in regioselectivity for the ortho-deuteration of **1b**, achieving 98% deuteration content (entry 3, Table 1), highlighting the critical role of doping P.

**Table 1:**
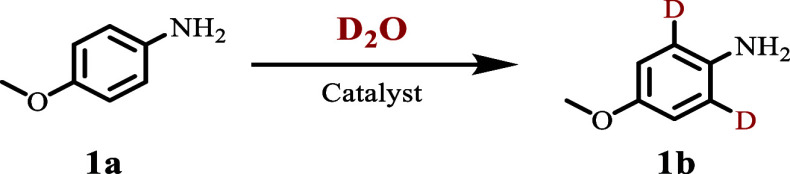
Deuterated Labeling of *p*-Anisidine over Carbon-Supported Metal Catalysts^a^

entry	catalysts	deuterium incorporation (%) **1b**	turnover frequency (h^–1^)^c^
1	Fe–N–C-800	<5	
2	Fe–S–C-800	67	28.0
3	Fe–P–C-800	98	131.3
4	Fe_NP_/C	97	4.5
5	Fe–P–C-700	79	91.1
6	Fe–P–C-900	91	100.9
7	Cu–P–C-800	63	53.3
8	Co–P–C-800	83	83.5
9	Ni–P–C-800	79	79.2
10	P–C	21	
11	without catalyst	<5	
12	Fe(NO3)3	<5	
13^b^	PC + Fe(NO_3_)_3_	<5	

a Reaction conditions: 0.5 mmol **1a**, 30 mg catalyst (0.16 mol % Fe for Fe–P–C-800), 1.5 mL D_2_O, 2 MPa H_2_, 120 °C, 12 h.

b 30 mg P–C (phosphorus-doped carbon) + 1 mg Fe­(NO_3_)_3_·9H_2_O.

c The calculation of TOF was based on 1 h reaction data, e.g., see time course for Fe–P–C catalyst in Figure S1; the Nuclear Magnetic Resonance (NMR) spectra of product **1b** are shown in Figure S19.

Notably, the Fe–P–C catalyst featuring a low loading of Fe (0.15 wt %) exhibited 30 times higher activity with turnover frequency (TOF) of 131.3 h^–1^, (entry 3, Table 1), compared to an Fe nanoparticulate catalyst with high Fe loading (4.9 wt %) as reported in previous works (TOF: 4.5 h^–1^, entry 4, Table 1),^24^ highlighting the high efficiency and potential of this single-atom catalytic system. To further enhance catalytic activity, we modulated the coordination environment of the active metal centers through variation of the pyrolysis temperature.^34–36^ The Fe–P–C material, pyrolyzed at 800 °C, exhibited superior performance (entries 3, 5–6, Table 1). To facilitate comparison, other heterogeneous catalysts (Cu, Co, Ni) were tested in the model reaction showing relatively lower reactivity (63–83% yield, entries 7–9, Tables 1 and S1), underscoring the importance of metal and phosphorus interaction. Minimal activity was observed in the absence of metal loading or catalysts (entries 10–11, Table 1). In addition, some commercial catalysts, including Fe powder, Ru/C, Pd/C, and Pt/C, were tested. None of these catalysts exhibited deuteration activity, although the noble metal catalysts produced some benzene hydrogenation byproducts. We also investigated and optimized critical reaction parameters, including the temperature and pressure (Figure S2). The catalytic process proved efficient using D_2_O as the deuteration source under 2 MPa of H_2_ at 120 °C. To verify that the Fe–P–C catalyst functions via a truly heterogeneous mechanism, control experiments were conducted using homogeneous Fe­(NO_3_)_3_·9H_2_O and the corresponding P–C support under optimized conditions, and both exhibited negligible catalytic activity (entries 12–13, Table 1). Additionally, a hot-filtration experiment conducted at 4 h (Figure S3) revealed no further conversion after removing Fe–P–C SAC, providing further evidence that the reaction proceeded exclusively heterogeneously.

### Structural Characterization of the Fe–P–C Catalyst

In order to elucidate the fundamental structural features responsible for the superior performance of the Fe–P–C catalyst, extensive characterization analyses were conducted. The inductively coupled plasma optical emission spectroscopy (ICP-OES) result revealed that the optimal Fe–P–C catalyst contained a low Fe loading of 0.15 wt % (Table S2). Only two broad peaks at 23° and 42°, corresponding to the reflections of the (002) and (101) lattice planes of carbon, respectively, were observed in the X-ray diffraction (XRD) pattern (Figure S4).^37^ The weak and broad (101) reflection suggests that a lower degree of graphitic crystallinity and more defects were formed in the carbon matrix after introducing P, which was further confirmed by the broadening of the D and G bands at 1325 cm^–1^ and 1589 cm^–1^, respectively, in the Raman spectrum (Figure S5).^38^ Furthermore, low-magnification scanning transmission electron microscopy (STEM) (Figures 2a and S6) images revealed no detectable nanoparticles, and the XRD pattern showed no diffraction peaks corresponding to Fe or FeO_*x*_ phases, indicating that the Fe species are highly dispersed on the carbon support without large Fe-containing crystalline particles. The material exhibited a Brunauer–Emmett–Teller (BET) surface area of 489 m^2^ g^–1^ (Figure S7), most likely due to the snowflake-like morphology of the carbon observed in the STEM images (Figure S6). Finally, sub-Ångström-resolution high-angle annular dark-field scanning transmission electron microscopy (HAADF-STEM) images (Figures 2b, and S8) provide direct visual evidence that the Fe species, appearing as bright dots, and are atomically dispersed across the P-doped carbon support. The ratio of signal intensities between Fe_1_ and P_1_ atoms was ≥2.0, in line with the atomic number (Z) contrast image-forming principle (Z^1.7^). The result indicates that the bright and dark spots are associated with Fe and P elements, respectively, pointing to the formation of Fe–P coordination structures in the Fe–P–C catalyst. Single-atom-sensitive electron energy loss spectroscopy (EELS) is also a powerful technique for identifying the central Fe atom and its coordinated P atoms.^39^ However, acquiring high-quality EELS data poses significant challenges due to limitations in both instrumental resolution and operational techniques. In our study, we utilized contrast differences to distinguish the heavier Fe atoms from the lighter P elements.

**Figure 2:**
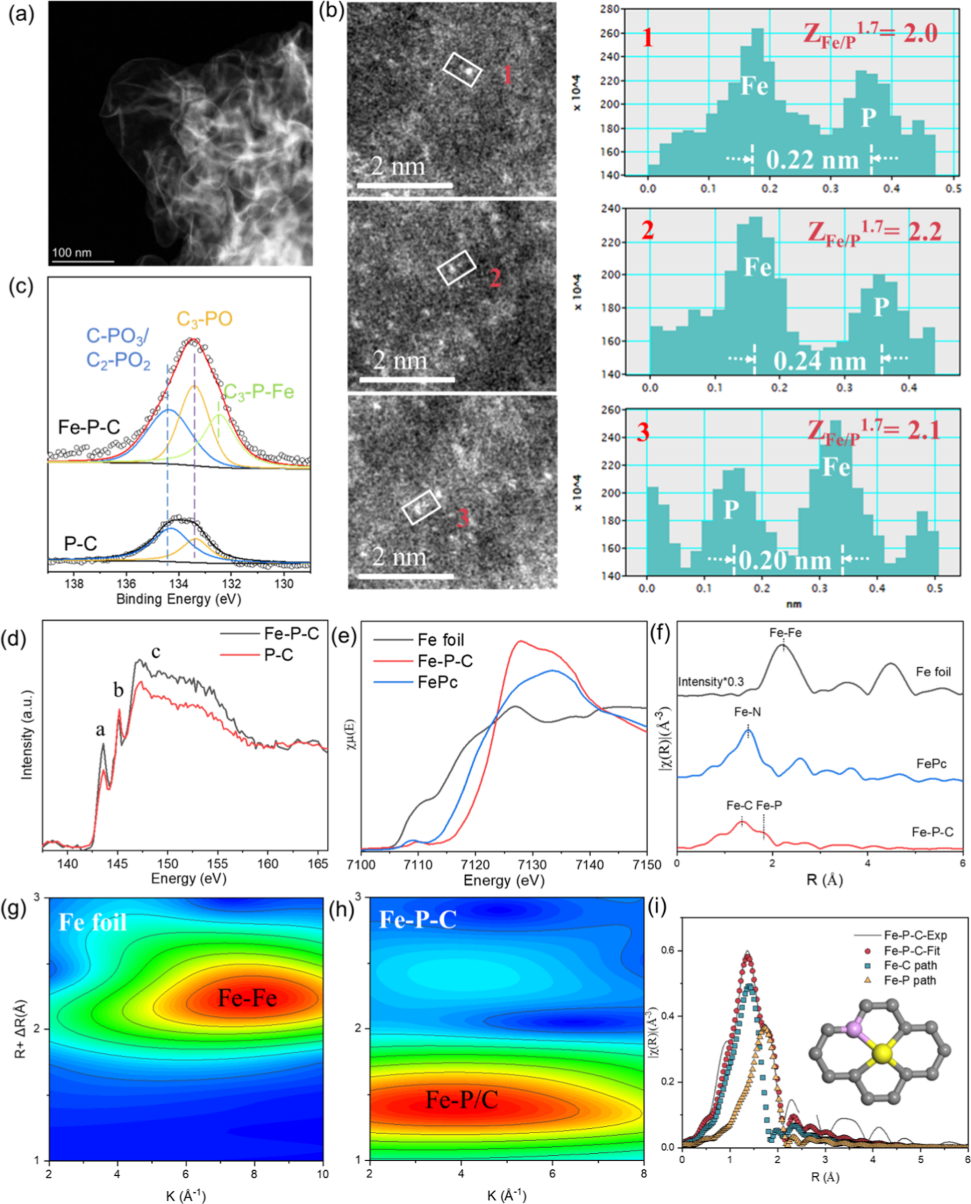
Structural characterization of the Fe–P C catalyst. (a) STEM image and (b) HAADF-STEM image, where the bright spots represent isolated Fe single atoms, (c) P 2p X-ray photoelectron spectrum, (d) normalized X-ray absorption near-edge spectrum (XANES) at the P *L*_2,3_-edge, (e) normalized XANES at the Fe *K*-edge, (f) *k* ^2^-weighted Fourier transform extended X-ray absorption fine structure spectrum (EXAFS) in *r*-space (FePc = iron phthalocyanine), (g,h) wavelet transformation analysis of the *k* ^2^-weighted EXAFS signal, and (i) EXAFS fitting curve in the range of 1.0–2.1 Å, shown in *k* ^2^-weighted *r*-space of the Fe–P–C catalyst.

To gain further insights into the interactions between Fe and P, X-ray photoelectron spectroscopy (XPS) and X-ray absorption spectroscopy (XAS) were employed. The Fe–P–C catalyst exhibits a significantly higher P content (3.2 at. %) than the pristine P–C (1.9 at. %), accompanied by negatively shifted binding energies and the appearance of Fe–P species in the P 2p XPS spectra (Figure 2c and Table S3), strongly suggesting the formation of Fe–P coordination environments within the carbon matrix. Low-energy peaks **a** and **b** in the P *L*_2,3_-edge X-ray absorption near-edge structure (XANES) spectrum are attributed to transitions of electrons from the spin–orbit split 2p_3/2_ and 2p_1/2_ levels into the lowest unoccupied 3s-like antibonding state (Figure 2d),^40^ showing a pronounced increase upon P incorporation and suggesting electron transfer from P to Fe, likely resulting from Fe–P orbital hybridization. The higher-energy side peak **c**, located around 147–155 eV, is attributed to 2*p* → 3*d* transitions and is sensitive to the local chemical environment of phosphorus. The higher intensity of this broad peak in Fe–P–C compared to P–C further suggests a potential Fe–P interaction. The XANES of the Fe *K*-edge is presented in Figure 2e. The adsorption threshold E_0_ for the Fe–P–C sample is higher than iron phthalocyanine (FePc) yet lower than Fe_2_O_3_, indicating that the Fe atoms carry partial positive charges (+δ, where 2 < δ < 3; Figure 2e), in agreement with the XPS characterization (Figure S9). Furthermore, the coordination environment of the Fe atoms was investigated by extended X-ray absorption fine structure (EXAFS) spectroscopy. As illustrated in the *k* ^2^-weighted Fourier-transformed EXAFS spectra at the Fe K-edge (Figure 2f), the Fe–P–C catalyst displays one dominant peak at ∼1.36 Å (not phase-corrected) with a small shoulder at ∼1.82 Å (not phase-corrected), which can be assigned to Fe–C and Fe–P coordination, respectively, with the latter likely resulting from the relatively longer Fe–P bond length.^41–43^ The Fe–Fe peak at ∼2.21 Å (not phase-corrected) seen in the Fe foil was not detected in Fe–P–C, confirming the atomic dispersion of Fe. To further identify the coordination atoms in Fe–P–C, two-dimensional EXAFS wavelet transform analysis was performed, providing high resolution in both ***k*** and ***r*** spaces (Figures 2g,h and S10).^44,45^ A broad lobe in the range 1.0–2.0 Å was observed for Fe–P–C, which is clearly distinct from those of the Fe foil (1.8–2.8 Å) and FePc (1.0–1.8 Å), supporting the assignment of the scattering contribution to a heavier coordinating element (P) around a mononuclear Fe center.^46,47^ Based on the detailed characterization shown above, the formation of Fe–P bonds in Fe–P–C could be corroborated. Least-squares fitting of the EXAFS data yielded quantitative structural parameters for the first coordination shell of Fe, indicating Fe–C and Fe–P coordination numbers of 2.9 and 1.1, and average Fe–C and Fe–P bond distances of 1.92 Å and 2.26 Å, respectively (Figure 2i and Table S4). The fitting results suggest each Fe atom was isolated by one P atom and three C atoms and Fe–P_1_C_3_ entities are likely formed in the material (see the inset structure in Figure 2i).

### Mechanism and Density Functional Theory (DFT) Calculations

To elucidate the specific role of the Fe–P pair site in the deuteration reaction, a series of targeted poisoning experiments were performed (Figure S11). A pronounced decline in catalytic activity was observed upon sulfur poisoning of the P sites, suggesting that phosphorus not only tunes the electronic properties of the Fe active site but also may be directly involved in the catalytic mechanism. Additionally, the reaction was inhibited when the Fe site was poisoned with SCN^–^, underscoring the critical involvement of Fe in the deuteration process. To gain a deeper understanding of the catalytic roles of the Fe and P sites in activating H_2_O and the substrate *p*-anisidine on the Fe–P pair, DFT calculations were performed. By systematic method tests (Figure S12), the results calculated by Perdew–Burke–Ernzerhof (PBE) are shown in Figure 3.

**Figure 3:**
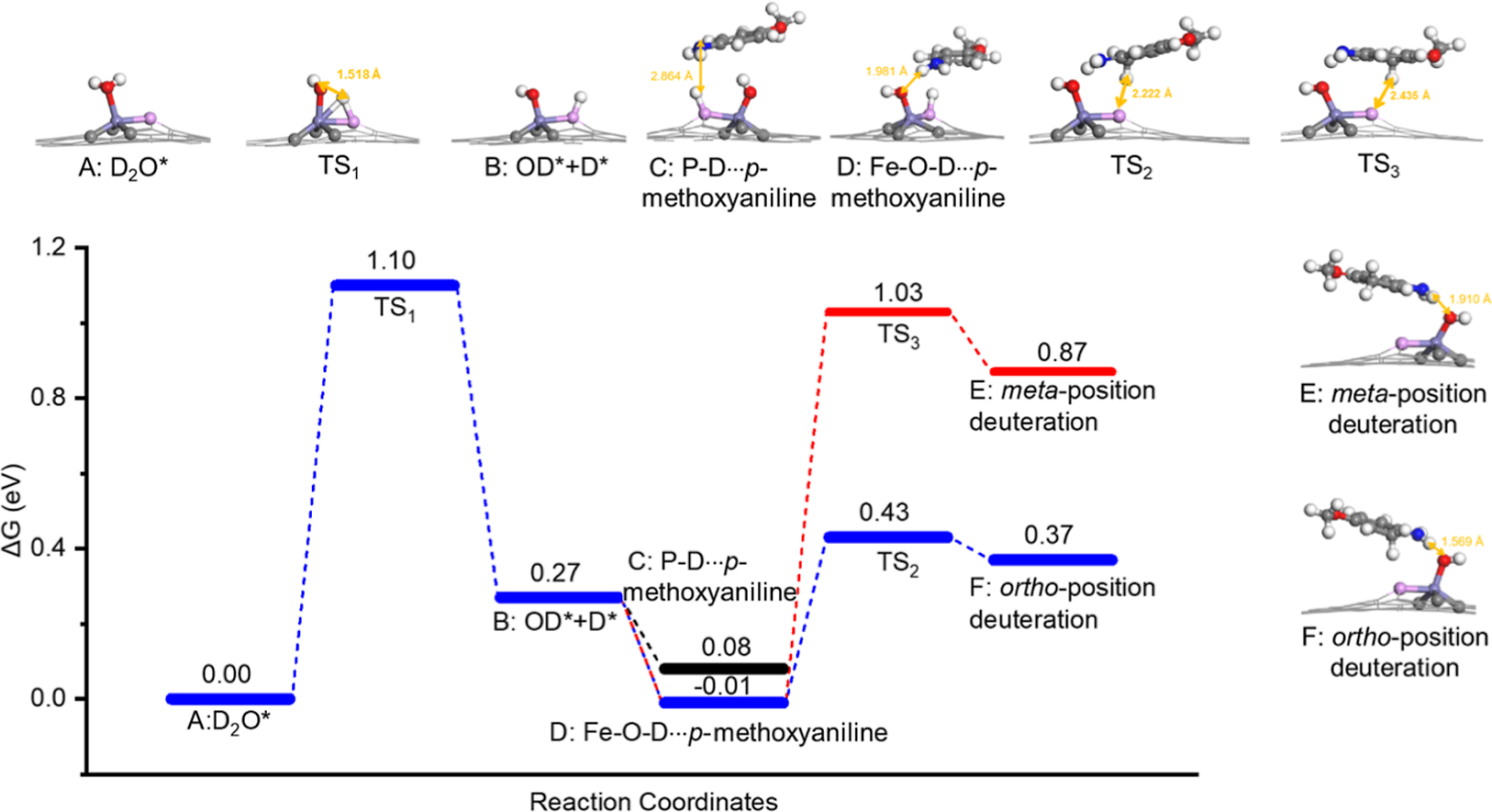
Optimized structures of adsorbed intermediates (top) and relative Gibbs free energy (Δ*G*) profiles of protonation on FeP_1_C_3_ (bottom) (purple/Fe; pink/P; gray/C; blue/N; red/O; white/D).

DFT calculations reveal that on the Fe–P catalytic pair, D_2_O can be adsorbed (A) and is followed by a transition state (TS_1_) with a Gibbs free energy barrier of 1.10 eV, suggesting that the dissociation of D_2_O to form OD and D is the rate-determining step.^24^ It is then dissociated into OD* at the Fe Lewis acid site and D* at the P site (B), respectively, which is endergonic by 0.27 eV. Subsequently, the substrate *p*-methoxyaniline introduces an amino group (−NH_2_), which can potentially form hydrogen bonding with either P-D* (C) or Fe-OD* (D). The results indicate that the –NH_2_ group of *p*-methoxyaniline prefers to interact with Fe-OD*, forming a Fe–O-D···H–NH hydrogen bonding with a bond length of 1.981 Å (D), while the P-D···H–NH hydrogen bonding formed by the –NH_2_ group and P-D* (C, 2.864 Å) is less stable by 0.09 eV. Based on this optimized configuration (D) and from the remote distance, the meta-position H is more spatial farther from P-D* (2.927 Å, Figure S13), making its deuteration more challenging, while the ortho-position H is closer (2.459 Å, Figure S13), facilitating easier deuteration. We further calculated the deuteration on the ortho and meta positions of *p*-methoxyaniline by the D species on P-D*. The ortho-position deuteration (F) is more favorable thermodynamically (0.38 vs 0.88 eV) and kinetically (0.44 vs 1.04 eV) than the meta-position deuteration (E). The stronger ortho-position deuteration over the meta-position deuteration can be rationalized by the resonance structure and stability of the protonated isomer (Figure S14). The primary distinction between the dissociation of H_2_O and D_2_O lies in their Gibbs free energy barriers, which are 1.03 and 1.10 eV, respectively. This difference is illustrated and explained in Figure S15. And the results including the solvent effects and weak intermolecular forces are shown in Figure S16. Overall, all of these calculation results provide a reliable explanation for the exclusive regioselective deuteration observed at the ortho-position of the aniline ring, which aligns well with our experimental findings (Table 1).

### Synthesis of Diverse Deuterated Products

To explore the applicability of the novel Fe–P–C SAC system, a wide range of substrates were tested under optimized conditions, yielding the corresponding deuterium-labeled products with good to excellent yields. As shown in Figure 4a, various ortho-, meta-, and para-substituted anilines bearing either electron-donating or electron-withdrawing groups were efficiently labeled with high deuterium contents (most showing >90% selectivity, **2b**–**10b**). Functional groups such as methoxy (**3b**, **9b**), halides (**4b**–**6b**), and more challenging ones like sulfur (**7b**), which often cause dehalogenation side-reactions and can poison metal catalysts, were well tolerated. Notably, the deuteration of more difficult substrates, including diamines (**11b**) and weakly basic secondary amine (**12b**–**15c**), also proceeded successfully, yielding the corresponding deuterated anilines.

**Figure 4:**
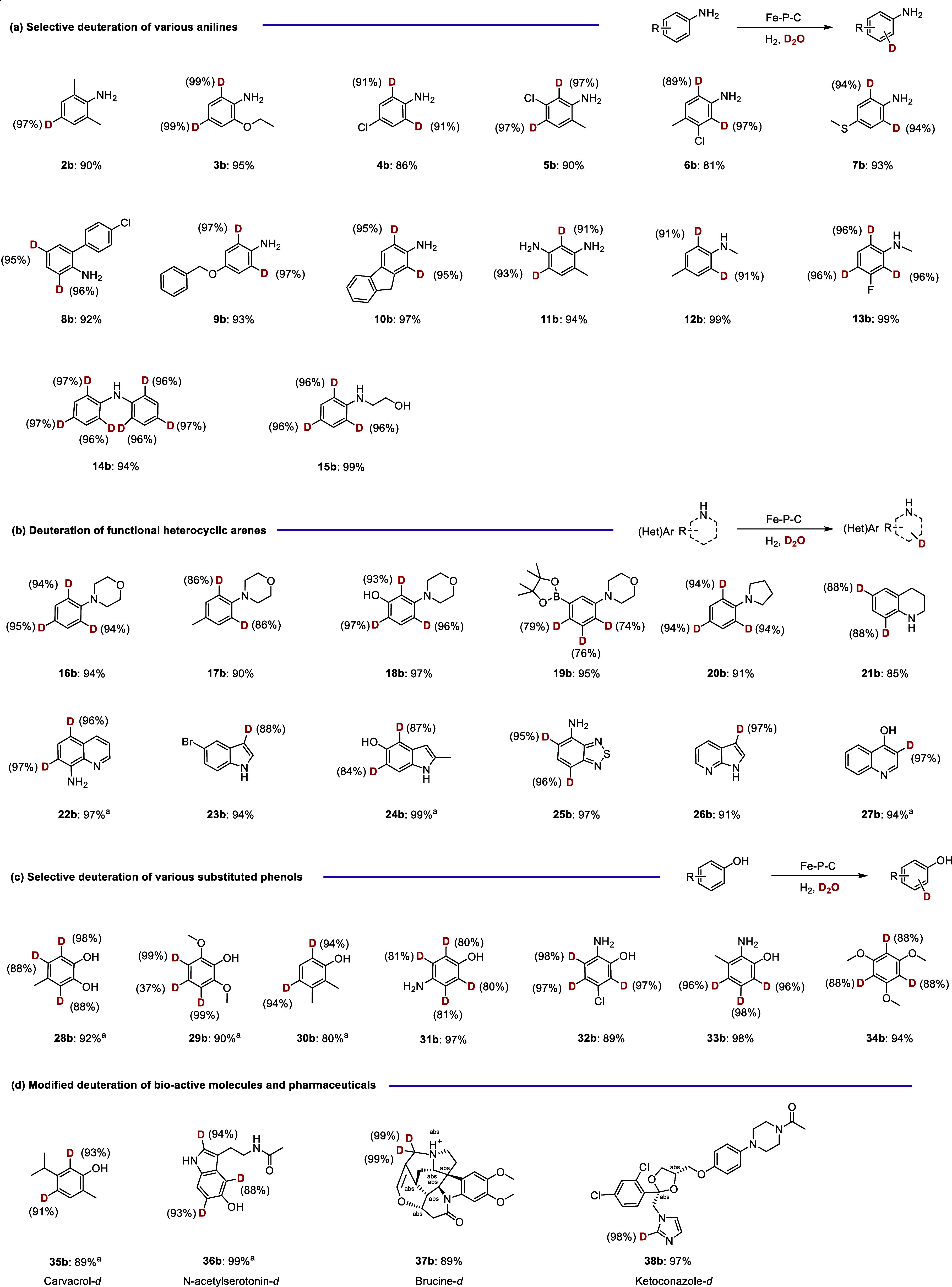
Exploring substrate scope through deuteration of a range of different anilines (a), heterocyclic arenes (b), phenols (c), and bioactive molecules (d). Reaction conditions: 0.25 mmol **1a**, 15 mg Fe–P–C-800 catalyst, 1.5 mL of D_2_O, 2 MPa H_2_, 120 °C, 12 h; ^a^ 30 mg of Fe–P–C-800 catalyst, 140 °C. Subscripts under the product represent isolated yields; the numbers around D indicate the degree of deuterium incorporation; the NMR spectra of the products are provided in Figures S20–S56.

As outlined in the introduction, nitrogen-containing (hetero)­arenes are versatile scaffolds widely employed in the synthesis of various agrochemical and pharmaceutical compounds: 82% of FDA-approved drugs contain a nitrogen heterocyclic motif.^48^ Given this, the reactivity of a wide range of functionalized nitrogen heterocycles was further investigated (Figure 4b). Remarkably, the Fe–P–C catalyst facilitated the efficient deuteration of 12 distinct heterocycles, exhibiting outstanding chemo- and regioselectivity (**16b**–**27b**). Functional group-containing tertiary amines, such as those with methyl, phenolic hydroxyl, and boronate ester groups, were deuterated with excellent results (**16b**–**19b**) without significant decomposition and occurrence of side reactions, a common issue when using a boronate ester-substituted group. Even challenging heterocyclic anilines, including tetrahydropyrrole, piperidine, and unsaturated pyrrole or pyridine (**20b**–**24b**), were efficiently deuterated by using this catalyst system. Importantly, substrates bearing multiple sulfur and nitrogen atoms—typically known to poison metal catalysts—also performed well under the standard reaction conditions (**25b**–**26b**). Additionally, the synthesis of deuterated hydroxyl-substituted pyridine (**27b**), which is a common motif in biologically active and natural products, was successfully achieved.

Phenolic hydroxy compounds are particularly significant because of their crucial role in regulating various biological functions.^49^ Typically, these substrates exhibit a lower reactivity compared to that of anilines. However, with our Fe–P–C catalytic system, regioselective deuterium incorporation for most substrates was observed (Figure 4c). Even challenging methyl-substituted catechol (**28b**) and electron-rich aminophenols (**29b**–**33b**) achieved a high D content (up to 99%). Additionally, 1,3,5-trimethoxybenzene (**34b**), a biomarker and a xenobiotic metabolite of flavonoid consumption in humans, was efficiently deuterated, yielding the corresponding deuterated biomolecule.

To underscore the potential of the heterogeneous Fe–P–C catalyst in modifying complex and sensitive molecules frequently encountered in the life sciences, we evaluated its performance in the deuteration of four representative natural products and drug molecules. As shown in Figure 4d, the deuteration transformation of carvacrol **35b**, *N*-acetylserotonin **36b**, Brucine **37b**, and ketoconazole **38b** proceeded smoothly with excellent D content (88–99%). The catalytic efficacy demonstrated through our novel methodology provides a powerful synthetic tool for the synthesis of deuterated drugs.

### Scale-Up Application and Recycling Studies

Scale-up synthesis of catalysts is a key barrier to transitioning SACs from an academic concept to a practical solution for industrial chemical processes. Therefore, we applied our simple mixing, calcination, and etching protocol to prepare approximately 4.8 g of the Fe–P–C catalyst (details available in the Supporting Information). Structural characterization via XRD and HAADF-STEM unequivocally demonstrated the successful scale-up synthesis (Figures 5a,b and S17). Beyond catalyst synthesis, the recyclability and scalability of the deuteration reaction are also crucial for any heterogeneous catalyst application, given the simplified product purification process. As shown in Figure 5c, our scale-up synthesized Fe–P–C SAC demonstrates excellent catalytic performance and can be efficiently recycled at least five times without any noticeable loss of activity, even at the gram scale. More importantly, ICP analysis of the hot-filtrated reaction solution confirms the absence of Fe leaching, while electron microscopy of the spent catalyst (Figure S18) shows no Fe cluster formation, further demonstrating its stability throughout the reaction. These findings underscore the excellent scalability and stability of the Fe–P–C SAC, representing a substantial advantage over homogeneous catalytic systems and a critical factor for potential industrial applications.

**Figure 5:**
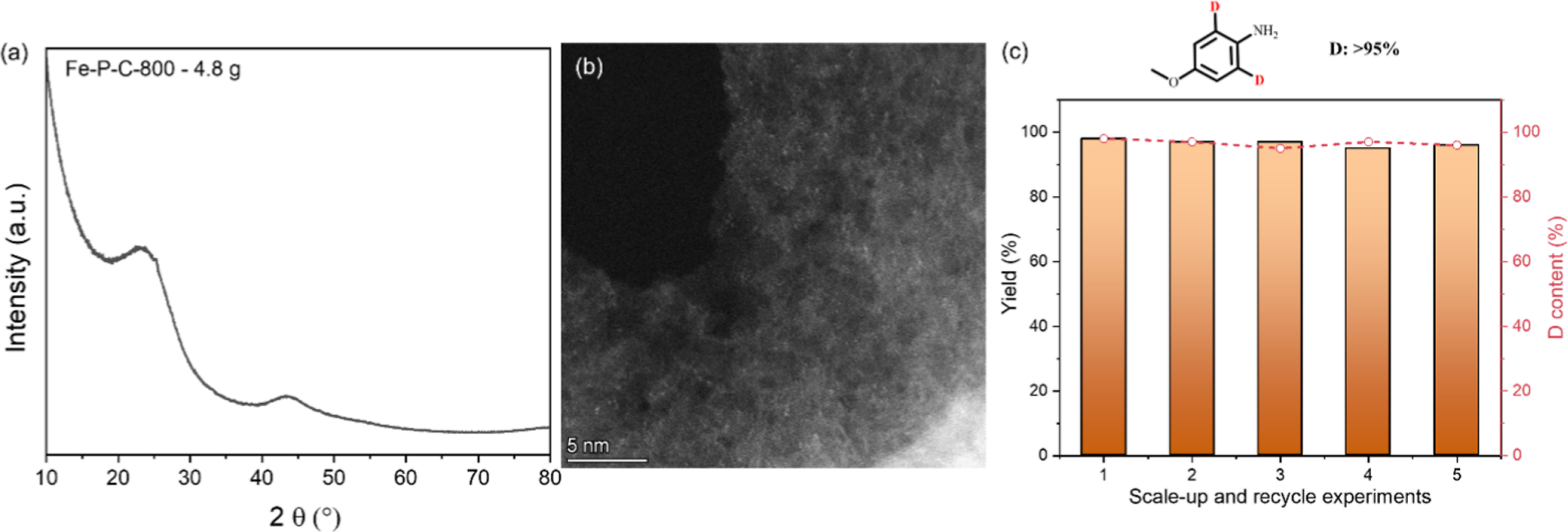
Substrate scope. (a) XRD and (b) HRTEM of scale-up of the Fe–P–C catalyst, (c) gram-scale recycle experiments. ^a^ Reaction conditions: 2 g **1a**, 1 g Fe–P–C-800 catalyst, 50 mL D_2_O, 3 MPa H_2_, 120 °C, 24 h.

Furthermore, the catalytic performance of this Fe–P–C SAC has been demonstrated to exceed that of previous homogeneous and heterogeneous catalytic systems (Table S5). From a sustainable perspective, iron is an ideal catalyst system for deuteration labeling due to its abundance (making up 34.5% of Earth’s mass), low price, and minimal toxicity. The Fe–P–C SAC’s exceptional catalytic performance, ease of recycling, scalability, and environmental friendliness are noteworthy advantages.

## Conclusions

In summary, a novel Fe single-atom catalytic system has been developed, enabling the highly efficient deuteration of (hetero)­arenes using D_2_O as a deuterium source under mild conditions. This system demonstrates superiority over previous homogeneous and heterogeneous catalytic systems. The Fe–P pair site activates D_2_O forming adsorbed *D species. These species then attack the ortho position of the adsorbed aniline, forming a protonated intermediate and achieving selective deuteration. The catalyst exhibits high regioselectivity for deuterium labeling of anilines, heterocycles, and phenol derivatives, with broad functional group tolerance, including bioactive and pharmaceutical molecules. Notably, the Fe–P–C SAC’s low cost, environmentally friendly nature, and ease of recycling are key advantages, making it an attractive option for a wide range of organic transformations.

## Methods

### Chemicals

Phytic acid solution (50 wt % in H_2_O) was purchased from Tokyo Chemical Industry Co., Ltd., while AEROSIL fumed silica was acquired from Evonik. Fe­(NO_3_)_3_·9H_2_O and *p*-anisidine were sourced from Sigma-Aldrich, and D_2_O was purchased from Eurisotop.

### Catalyst Preparation

All materials were prepared through a template-sacrificial approach. For example, the Fe–P–C-800 catalyst was synthesized by dissolving 30 mg of Fe­(NO_3_)_3_·9H_2_O and 4 g of a 50 wt % phytic acid solution in 50 mL of H_2_O. The mixture was refluxed at 120 °C for 30 min, after which 2.0 g of fumed silica was added, and the solution was stirred at 120 °C for an additional 12 h. Subsequently, the reflux condenser was removed, allowing for the slow evaporation of H_2_O over a 24 h period. Once the solvent had evaporated and the solid mixture was fully dried, it was transferred to a crucible and heated in a furnace under argon flow. The temperature was increased to 800 °C at a rate of 5 °C/min and maintained for 2 h. The black powder obtained was then washed twice with 500 mL of 1 mol/L NaOH solution under 90 °C for 12 h to etch the silica template. The residue was rinsed with 2 L of water and dried at 80 °C for 12 h. The resulting material was designated Fe–P–C-800. In this manuscript, Fe–P–C refers to Fe–P–C-800 unless specified otherwise. For comparison, similar samples pyrolyzed at 700 and 900 °C were designated Fe–P–C-700 and Fe–P–C-900, respectively.

Using the same procedure, Fe–N–C and Fe–S–C catalysts were prepared by employing 1,10-phenanthroline as the N-containing precursor and 2,2′-bithiophene as the S-containing precursor, respectively. To prepare the P–C sample, triphenylphosphine was employed as the P-containing precursor, and no Fe salts were introduced during the synthesis. This method ensured the production of tailored materials for further catalytic applications.

Scale-up synthesis of 4.8 g Fe–P–C: 600 mg of Fe­(NO_3_)_3_·9H_2_O and 80 g of 50 wt % phytic acid solution were dissolved in 1000 mL of H_2_O, followed by the addition of 20 g fumed silica; the mixture was stirred and refluxed at 120 °C for 24 h to obtain a homogeneous slurry. Then the water in the mixture was removed by rotary evaporation and then placed in a drying oven at 160 °C for another 48 h. The obtained black lumps were ground into fine powder and then transferred to a crucible and heated in a furnace under argon flow. The temperature was controllably ramped at a rate of 5 °C min^–1^ to 800 °C and maintained at 800 °C for 2 h. When it was cooled to room temperature, the obtained carbon@silica composite was treated with 1 mol/L NaOH solution at 90 °C for 12 h, followed by filtration and washing with ultrapure water (2 L); this procedure was repeated three times to completely remove the silica template. Finally, the powder was washed with ultrapure water (2 L) twice and then dried under vacuum at 80 °C for 12 h.

### Reaction Tests

A typical reaction was carried out by combining 0.5 mmol of *p*-anisidine, 30 mg of the Fe–P–C catalyst, and 1.5 mL of D_2_O in an 8 mL vial with a septum cap. A needle was inserted through the septum to allow the introduction of gaseous reactants. The autoclave was then sealed and purged three times with H_2_, before being pressurized with 20 bar of H_2_. The reaction was conducted under magnetic stirring at 500 rpm and heated to 120 °C for 12 h. Once the reaction stopped, the reaction mixture was extracted with ethyl acetate (3 × 4 mL). The combined organic layers were vigorously shaken with 4 mL of water to hydrolyze any ND_2_ groups to NH_2_. The aqueous layer was subsequently extracted again with ethyl acetate (3 × 4 mL). The pooled organic phases were dried over anhydrous Na_2_SO_4_, and the solvent was evaporated under reduced pressure. The purified product was analyzed by NMR spectroscopy to determine the degree of deuterium incorporation.

The yield of **1b** (Y_**1b**_) and the deuterium content of **1b** (D_**1b**_) were calculated by using the following equations

$$Y_{1b}\left(\%\right)=\left({{mol}_{1b}}_{isolated}\right)/\left({{mol}_{1a}}_{fed}\right)\times 100$$

D_**1b**_ (%) = (1–corresponding ^1^H NMR peak area_**1b** produced_/(corresponding ^1^H NMR peak area_**1a** standard_)) × 100.

The TOF calculation formula: TOF (h^–1^) = $\frac{{mmol}_{1h\;D\;content\;of\;1a}}{{mmol}_{Fe}\times 1h}$ .

### Reusability Test

After each reaction cycle, the catalyst was recovered by centrifugation followed by sequential washing with acetonitrile (3 × 50 mL) and ethanol (2 × 50 mL). The cleaned catalyst was then dried at 80 °C for 2 h and reused directly in the subsequent reaction.

The actual Fe loadings were determined by ICP-OES using an IRIS Intrepid II XSP instrument (Thermo Electron Corporation).

XRD analysis was performed on a PANalytical X’pert diffractometer equipped with Cu Kα radiation source (λ = 0.15432 nm), operating at 40 kV and 40 mA. Data were collected over a scanning angle (2θ) of 10°–80°.

STEM and energy-dispersive X-ray spectroscopy (EDS) analyses were conducted by using a JEOL JEM-2100F microscope operated at 200 kV. The instrument was equipped with an Oxford Instruments ISIS/INCA EDS system featuring a Pentafet Ultrathin Window detector.

Aberration-corrected high-angle annual dark-field STEM (AC-HAADF-STEM) was carried out on a JEOL JEM-ARM200F instrument equipped with a CEOS probe corrector, offering a spatial resolution of 0.08 nm. Prior to imaging, the sample was ultrasonically dispersed in ethanol for 15–20 min, and a drop of the suspension was deposited onto a copper TEM grid coated with a holey carbon film.

XPS measurements were performed on a Thermo ESCALAB 250 spectrometer equipped with an Al Kα radiation source. The C 1s peak at 284.0 eV was used as an internal standard for the charge correction.

Soft XAS (soft-XAS) measurements at the P *L*_2,3_-edge were carried out at the MCD-A beamline of the National Synchrotron Radiation Laboratory in Hefei, China.

X-ray absorption spectra (XAS), including X-ray absorption near edge structure (XANES) and extended X-ray absorption fine structure (EXAFS) at the Fe *K*-edge, were measured at beamline 14 W of the Shanghai Synchrotron Radiation Facility (SSRF), China. The beam was monochromated using a Si(111) crystal, and the energy calibration was performed using a Fe foil. Data were collected at room temperature in transmission mode.

NMR spectra were recorded by using CDCl_3_ or DMSO-*d*_6_ as deuterated reagents on a 300/400 MHz Bruker DRX-300/400 spectrometer.

Spin-polarized DFT calculations were performed using the Vienna ab initio simulation package (VASP).^50^ The projector augmented wave pseudopotentials (PAW)^51^ were used to describe the interaction between atomic cores and valence electrons. To evaluate the reliability of our computational approach, we performed optimization of the reaction potential energy surface using several exchange–correlation functionals, including Perdew–Burke–Ernzerhof (PBE)^52^ and revised PBE from Hammer et al. (RPBE)^53^ and from Zhang and Yang (revPBE).^54^ Additionally, we employed PBE including van der Waals dispersion corrections with the lates parameter (D3)^55^ to account for van der Waals interactions and utilized an implicit solvent model to incorporate solvation effects with VASPSol mode^56^ The cutoff energy was set by 500 eV. The FeP_1_C_3_ site was constructed in a 6 × 6 periodic graphene supercell according to the experimental coordination number. The vacuum layers were set at 20 Å. A 1 × 1 × 1 Gamma centered Monkhorst Pack *k*-point sampling was chosen.^57^ Geometry optimizations were pursued until the force on each atom falls below the convergence criterion of 0.02 eV/Å and energies were converged within 10^–5^ eV. The climbing-image nudged elastic band method^58^ in combination with the DIMER method^59^ was used to search the TS1, which was verified by only one imaginary frequency connecting the initial and transition states. All reported energetic data include the zero-point-energy (ZPE) correction. The energy profiles are corrected using the references of single H_2_O molecular, single *p*-methoxyaniline molecular, and clean slab. And the Gibbs free energies of periodic model system are estimated by VASPKIT code.^60^

## Supplementary Material



## Notes

Additional experimental details, methods, and some experimental results, including XRD, Raman, STEM, BET, XPS characterizations for the catalyst, and NMR spectra for all compounds (PDF)
