# Supplementary material for: Enhancing Activation of D2O for Highly Efficient Deuteration Using an Fe–P Pair-Site Catalyst
Source: JACS Au. 2025 May 21;5(6):2666–76. doi: 10.1021/jacsau.5c00257 (PMC12188401; doi:10.1021/jacsau.5c00257)
Supplement: Supplementary file 1 [file au5c00257_si_001.pdf]

Enhancing Activation of D_2O for Highly Efficient Deuteration using an Fe-P Pair-site Catalyst

Haifeng Qi[1, 2, 5], Yueyue Jiao[1, 3, 5], Qiang Wang[1], Nicholas F. Dummer[2], Jianglin Duan[1], Yujing Ren[4], Stuart H. Taylor[2], Haijun Jiao[1, *], Kathrin Junge[1, *], Graham J. Hutchings[2, *], and Matthias Beller[1, *]

[1] Leibniz-Institut für Katalyse e. V., Albert-Einstein-Straβe 29a, Rostock 18059, Germany.

[2] Max Planck-Cardiff Centre on the Fundamentals of Heterogeneous Catalysis FUNCAT, Translational Research Hub, Cardiff University, Maindy Road, Cardiff CF24 4HQ, UK.

[3] SINOPEC Research Institute of Petroleum Processing Co., Ltd., Beijing 100083, P. R. China.

[4] Interdisciplinary Research Center of Biology & Catalysis, School of Life Sciences, Northwestern Polytechnical University, Xi'an 710072, China.

[5] These authors contributed equally

* Corresponding authors: K. Junge (kathrin.junge@catalysis.de)

H. Jiao (haijun.jiao@catalysis.de)

G. J.Hutchings (hutch@cardiff.ac.uk)

M. Beller (matthias.beller@catalysis.de)

Inventory of Supporting Information

Methods..4

Figure S1...8

Figure S2...9

Figure S3...10

Figure S4...11

Figure S5...12

Figure S6...13

Figure S7...14

Figure S8...15

Figure S9...16

Figure S10...17

Figure S11...18

Figure S12...19

Figure S13...21

Figure S14...22

Figure S15...23

Figure S16...24

Figure S17...25

Figure S18...26

Table S1..27

Table S2..28

Table S3..29

Table S4..30

Table S5..31

Supplementary References..32

NMR Spectra...34-72

Methods

Materials. Phytic acid solution (50 wt.% in H_2O) was purchased from TCI. AEROSIL® fumed silica was acquired from Evonik. $Fe(NO_3)_3 \cdot 9H_2O$ and *p*-anisidine were obtained from Sigma-Aldrich. D_2O was purchased from Eurisotop. All other chemical reagents were used as received without further purification.

Catalyst preparation. All catalysts were synthesized using a template-sacrificial approach. For instance, the Fe-P-C-800 catalyst was prepared by dissolving 30 mg of $Fe(NO_3)_3 \cdot 9H_2O$ and 4 g of a 50 wt.% phytic acid solution in 50 mL of H_2O. The mixture was refluxed at 120 °C for 30 minutes, after which 2.0 g of fumed silica was added, and the solution was stirred at 120 °C for an additional 12 hours. Subsequently, the reflux condenser was removed, allowing for the slow evaporation of H_2O over a 24-hour period. Once the solvent had evaporated and the solid mixture was fully dried, it was transferred to a crucible and heated in a furnace under argon flow. The temperature was increased to 800 °C at a rate of 5 °C/min and maintained for 2 hours. The obtained black powder was then washed twice with 500 mL of 1 mol/L NaOH solution at 90 °C for 12 hours to remove the silica template. The residue was rinsed with 2 L of water until the filtrate became neutral and dried at 80 °C for 12 hours. The resulting sample was labeled Fe-P-C-800. Unless otherwise noted in the manuscript, Fe-P-C refers to Fe-P-C-800. Analogous samples pyrolyzed at 700 °C and 900 °C were labeled Fe-P-C-700 and Fe-P-C-900, respectively.

Following the same procedure, Fe-N-C and Fe-S-C materials were synthesized by utilizing N- and S-containing organic precursors, specifically 1,10-phenanthroline and 2,2'-bithiophene, respectively. For the synthesis of the P-C sample, triphenylphosphine, a P-containing organic precursor, was used without the addition of Fe salts. This method ensured the production of tailored materials for further catalytic applications.

Scale-up synthesis of 4.8 g Fe-P-C: 600 mg of $Fe(NO_3)_3 \cdot 9H_2O$ and 80 g of 50 wt.% phytic acid solution were dissolved in 1000 mL of H_2O, followed by the addition of 20 g fumed silica; the mixture was stirred and refluxed at 120 °C for 24 h to obtain a homogeneous slurry. Then the water in mixture was removed by rotary evaporation and then placed in a drying oven at 160 °C for another 48 h. The obtained black lumps were grounded into fine powder and then transferred to a crucible and heated in a furnace under argon flow. The temperature was controllably ramped at a rate of 5 °C min^{-1} to 800 °C, and maintained at 800 °C for 2 h. When cooling to room temperature, the obtained carbon@silica composite was treated with 1 mol/L NaOH solution at 90 °C for 12 h, followed by filtration and washing with ultrapure water (2 L); this procedure was repeated three times to completely remove the silica template. Finally, the powder was washed with ultrapure water (2 L) for twice and then dried under vacuum at 80 °C for 12 h.

Reaction tests. In a typical reaction, 0.5 mmol p-anisidine, 30 mg Fe-P-C catalyst, and 1.5 mL D_2O were placed in an 8 mL vial fitted with a magnetic stirring bar and septum cap. A needle was inserted through the septum to allow the entry of gaseous reagents. Up to seven such vials were placed in an alloy plate, which was then positioned inside a 300 mL steel Parr autoclave. After sealing, the autoclave was purged three times with N_2, followed by three purges with H_2, and then charged with 20 bar of H_2 at room temperature. The reaction mixture was stirred at 500 r/min and heated to 120 °C for 12 hours. Upon completion, the reaction mixture was extracted with EtOAc (3 × 4 mL). The organic phase was vigorously shaken with 4 mL of H_2O to convert ND_2 to NH_2, and the aqueous phase was further extracted with EtOAc (3 × 4 mL). The combined organic extracts were dried over anhydrous Na_2SO_4. The isolated product was then subjected to NMR analysis to determine the deuterium content.

The yield of **1b** ($Y_{\mathbf{1b}}$) and the deuterium content of **1b** ($D_{\mathbf{1b}}$) were calculated using the following equations:

$Y_{\mathbf{1b}}$ (%) = ($mol_{\mathbf{1b}\text{ isolated}}$)/ ($mol_{\mathbf{1a}\text{ fed}}$) * 100

$D_{\mathbf{1b}}$ (%) = (1-Corresponding ^{1}H NMR peak $area_{\mathbf{1b}\text{ produced}}$/ (Corresponding ^{1}H NMR peak $area_{\mathbf{1a}\text{ standard}}$)) * 100

The TOF calculation formula: TOF (h^{-1})=$\frac{mmol_{1h\ D\ content\ of\ 1a}}{mmol_{Fe}\times 1h}$.

Reusability test. After each reaction, the catalyst was separated from the reaction mixture by centrifugation. The collected catalyst was then washed three times with 50 mL of ethanol and dried at 80 °C for 2 hours. The resulting powder was subsequently used in the next batch reaction.

The actual Fe loadings were determined by **inductively coupled plasma spectroscopy (ICP-OES)** on an IRIS Intrepid II XSP instrument (Thermo Electron Corporation).

X-ray diffraction (XRD) analysis was carried out on a PANalytical X'pert diffractometer using Cu Kα radiation source (λ = 0.15432 nm) with a scanning angle (2θ) of 10°- 80°, operated at 40 kV and 40 mA.

Scanning transmission electron microscopy (STEM) and **energy dispersive X-ray spectroscopy (EDS)** experiments were performed on a JEOL JEM-2100F microscope operated at 200 kV, equipped with an Oxford Instruments ISIS/INCA energy-dispersive X-ray spectroscopy (EDS) system with an Oxford Pentafet Ultrathin Window (UTW) Detector.

The **aberration-corrected high-angle annual dark-field scanning transmission electron microscopy (AC-HAADF-STEM)** analysis was performed on a JEOL JEM-ARM200F equipped with a CEOS probe corrector, with a guaranteed resolution of 0.08 nm. Before microscopy examination, the sample was ultrasonically dispersed in

ethanol for 15-20 min, and then a drop of the suspension was deposited on a copper TEM grid coated with a thin holey carbon film.

X-ray photoelectron spectroscopy (XPS) spectra were obtained on a Thermo ESCALAB 250 X-ray photoelectron spectrometer equipped with Al Kα excitation source and with C as internal standard (C 1s = 284.0 eV).

Soft X-ray absorption spectroscopy (soft-XAS) spectra of P $L_{2,3}$-edge were performed at the beamline MCD-A at the National Synchrotron Radiation Laboratory (NSRL) in Hefei, China.

X-ray absorption spectra (XAS) including X-ray absorption near edge structure (XANES) and extended X-ray absorption fine structure (EXAFS) at Fe K-edge of the samples were measured at the beamline 14W of the Shanghai Synchrotron Radiation Facility (SSRF) in China. The output beam was selected by a Si(111) monochromator, and the energy was calibrated against a Fe foil. The data were collected at room temperature under transmission mode.

Nuclear Magnetic Resonance spectroscopy (NMR) spectra were recorded at room temperature in $CDCl_3$ or d^6-DMSO on a 300/400 MHz Bruker DRX-300/400 NMR spectrometer.

Density Functional Theory (DFT) calculations. Spin-polarized density functional theory (DFT) computations were performed by Vienna ab-initio simulation package (VASP)[1]. The projector augmented wave pseudo-potentials (PAW)[2] were used to describe the interaction between atomic cores and valence electrons. To evaluate the reliability of our computational approach, we performed optimization of the reaction potential energy surface using several exchange-correlation functionals, including Perdew–Burke–Ernzerhof (PBE)[3] and revised PBE from Hammer *et al.* (RPBE)[4] and from Zhang and Yang (revPBE)[5]. Additionally, we employed PBE including van der Waals dispersion corrections with the lates parameter (D3)[6] to account for van der Waals interactions and utilized an implicit solvent model to incorporate solvation effects with VASPSol mode[7] The cutoff energy was set by 500 eV. The FeP_1C_3 site was constructed in a 6 × 6 periodic graphene supercell according to the experimental coordination number. The vacuum layers were set by 20 Å. A 1 × 1 × 1 Gamma centered Monkhorst Pack k-point sampling was chosen[8]. Geometry optimizations were pursued until the force on each atom falls below the convergence criterion of 0.02 eV/Å and energies were converged within 10^{-5} eV. The climbing-image nudged elastic band (CI-NEB) method[9] in combination with the DIMER method[10] was used to search the transition state, which was verified by only one imaginary frequency connecting the initial and transition states. All reported energetic data include zero-point-energy (ZPE) correction. The energy profiles are corrected using the references of single H_2O molecular, single *p*-methoxyaniline molecular and clean slab. And the Gibbs free energies of periodic model system are estimated by

VASPKIT code[11].

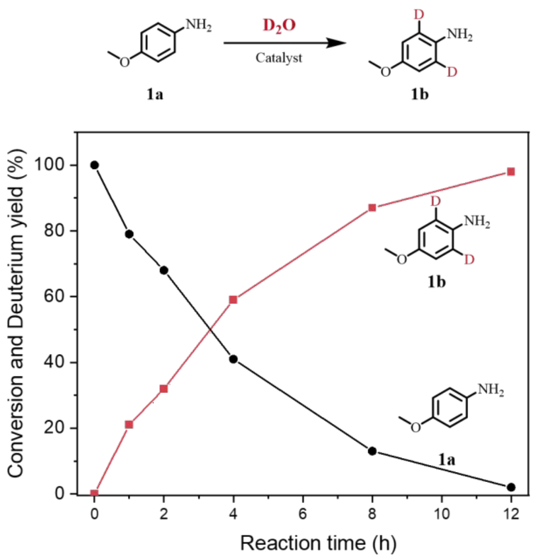

Figure S1. Conversion and deuterium yield as a function of reaction time in the presence of Fe-P-C. Reaction conditions: 0.5 mmol **1a**, 30 mg Fe-P-C-800, 1.5 mL D_2O, 2 MPa H_2, 120 ^oC, 12 h.

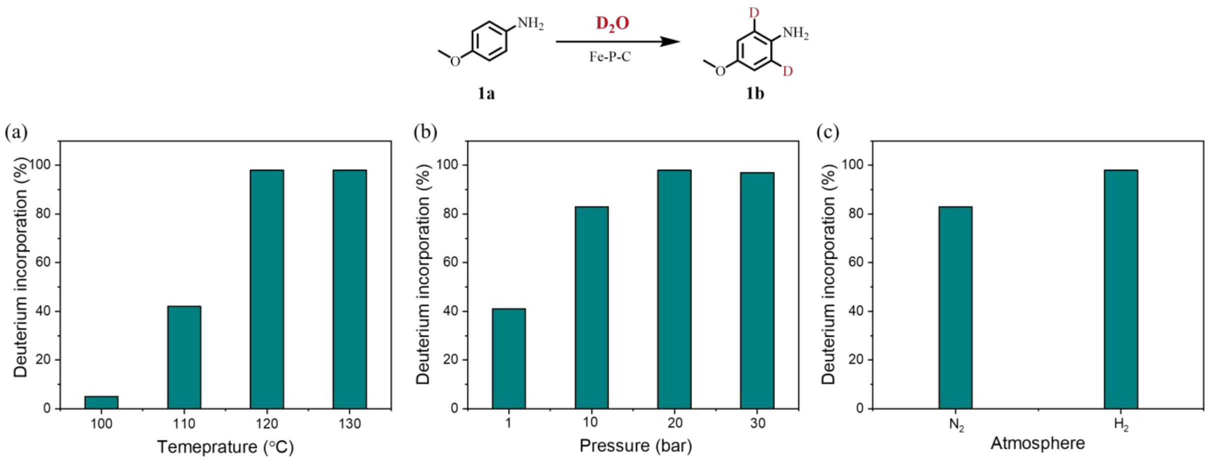

Figure S2. Optimization of reaction conditions in the presence of Fe-P-C. (a) Reaction temperature, (b) H_2 pressure, and (c) atmosphere. Standard reaction conditions: 0.5 mmol **1a**, 30 mg Fe-P-C-800, 1.5 mL D_2O, 2 MPa H_2, 120 °C, 12 h.

The reaction conditions, including temperature, H_2 pressure, and atmosphere, were systematically optimized. As depicted in Figures S2a-b, the hydrogenation process failed to proceed below 120 °C and 20 bar H_2. Optimal conditions were determined to be ≥ 120 °C and ≥ 20 bar H_2 pressure. Figure S2c illustrates the performance of this catalyst system is better in the presence of hydrogen, which probably due to in situ reduction and removal of surface adsorbed oxygen species on iron center as confirmed by previous work[12].

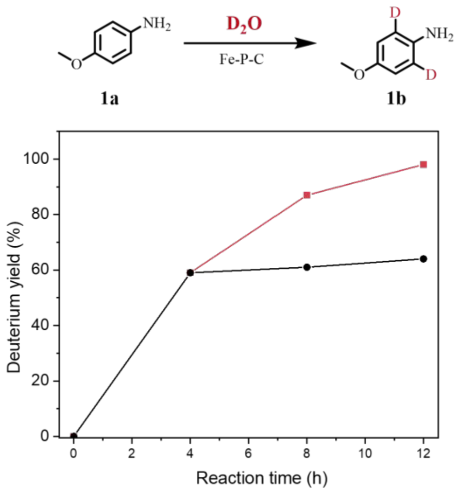

Figure S3. Hot-filtration experiment. Reaction conditions: 0.5 mmol **1a**, 30 mg Fe-P-C-800, 1.5 mL D_2O, 2 MPa H_2, 120 ^oC, defined time.

The hot-filtration experiment was conducted after a 4-hours standard reaction. The filtered solution was obtained at 80 °C to remove the Fe-P-C catalyst, after which the reaction mixture was allowed to proceed at 20 bar H_2 and 120 °C in the absence of solid Fe-P-C catalyst. No significant increase in the yield of **1b** product indicates the intrinsic nature of the heterogeneous catalytic process during deuteration.

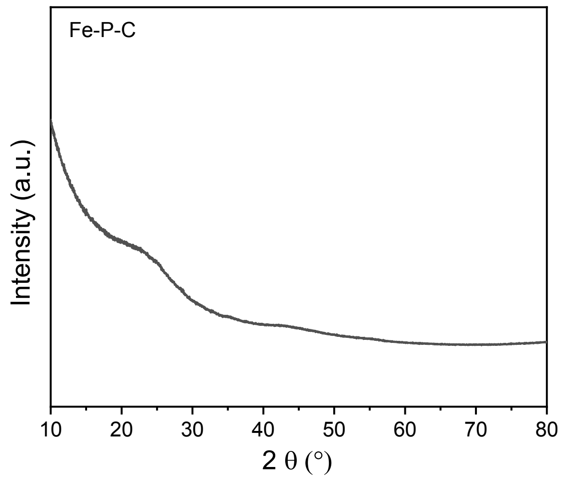

Figure S4. XRD pattern of Fe-P-C-800.

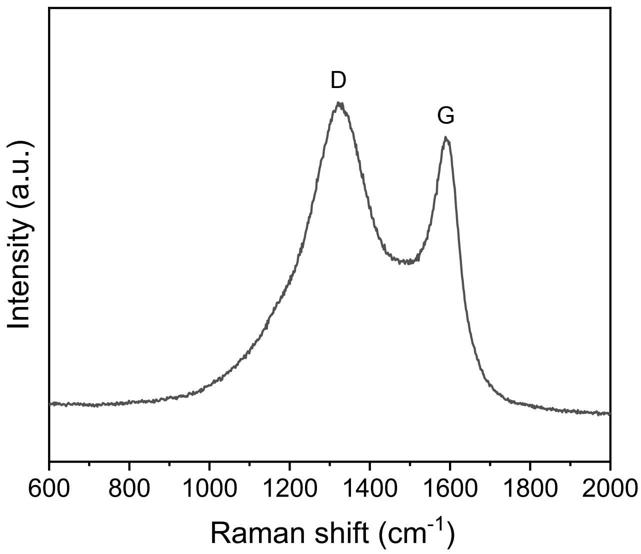

Figure S5. Raman spectra of Fe-P-C-800.

Raman spectra exhibits a vacancy or defect site-related D band at 1325 cm^{-1}, along with an in-plane sp^2-hybridized graphitic layer with E_{2g} symmetry-related G band at 1589 cm^{-1}. The corresponding intensity ratio of I_D/I_G values is 1.1, indicating the presence of an amorphous and defective carbon structure in Fe-P-C-800.

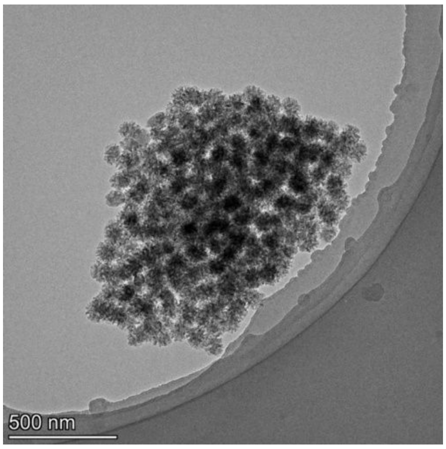

Figure S6. The STEM images of Fe-P-C-800 sample.

No obvious Fe or P-related nanoparticles or clusters can be detected in the Fe-P-C catalyst.

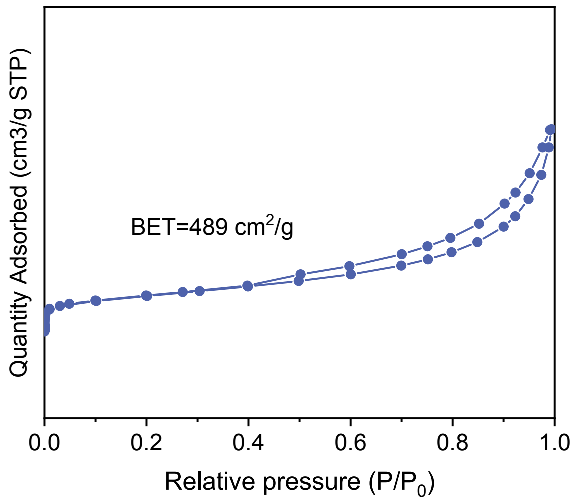

Figure S7. Brunauer-Emmett-Teller surface area of Fe-P-C sample.

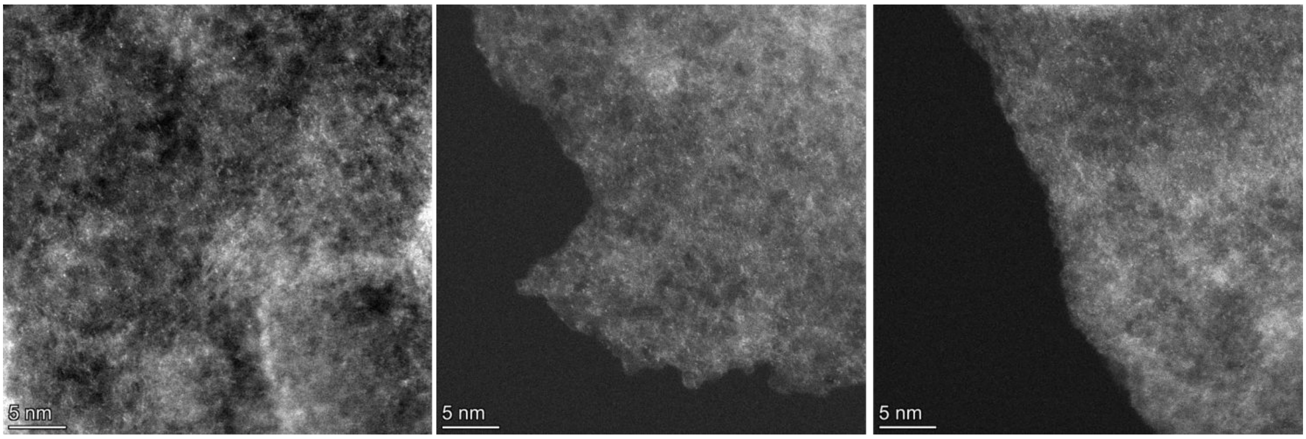

Figure S8. HADDF-STEM images of Fe-P-C sample.

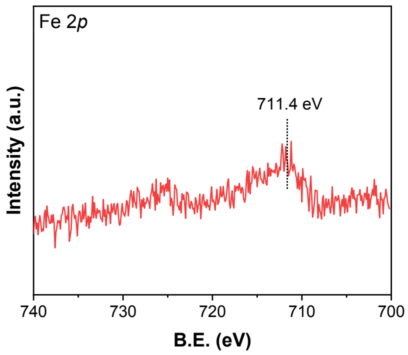

Figure S9. Fe *2p* X-ray photoelectron spectroscopy of Fe-P-C sample.

The Fe $2p_{3/2}$ binding energy in Fe-P-C is measured at 711.4 eV, suggesting that Fe atoms have an oxidation state < +3.

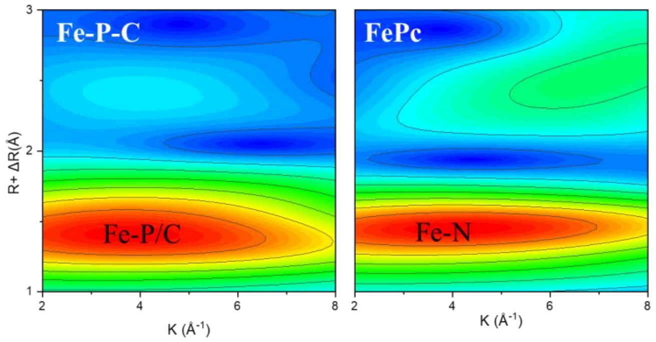

Figure S10. 2D EXAFS wavelet transform (WT) analysis of Fe-P-C and FePc samples.

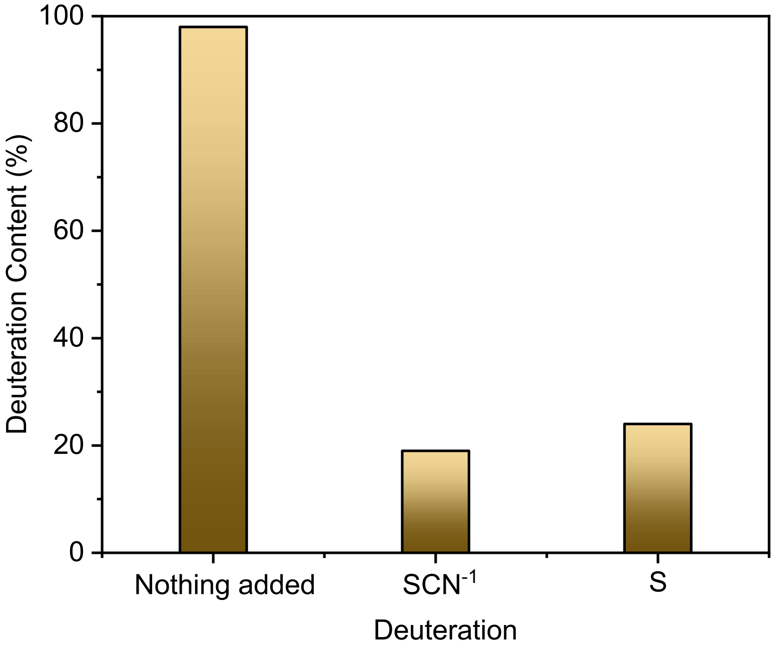

Figure S11. Poisoning experiments. Reaction conditions: 0.5 mmol substrate, 30 mg Fe-P-C-800 catalyst, 2 equiv. corresponding poisoning chemical (KSCN or S), 1.5 mL D_2O, 20 bar H_2, 120 °C, 12 h.

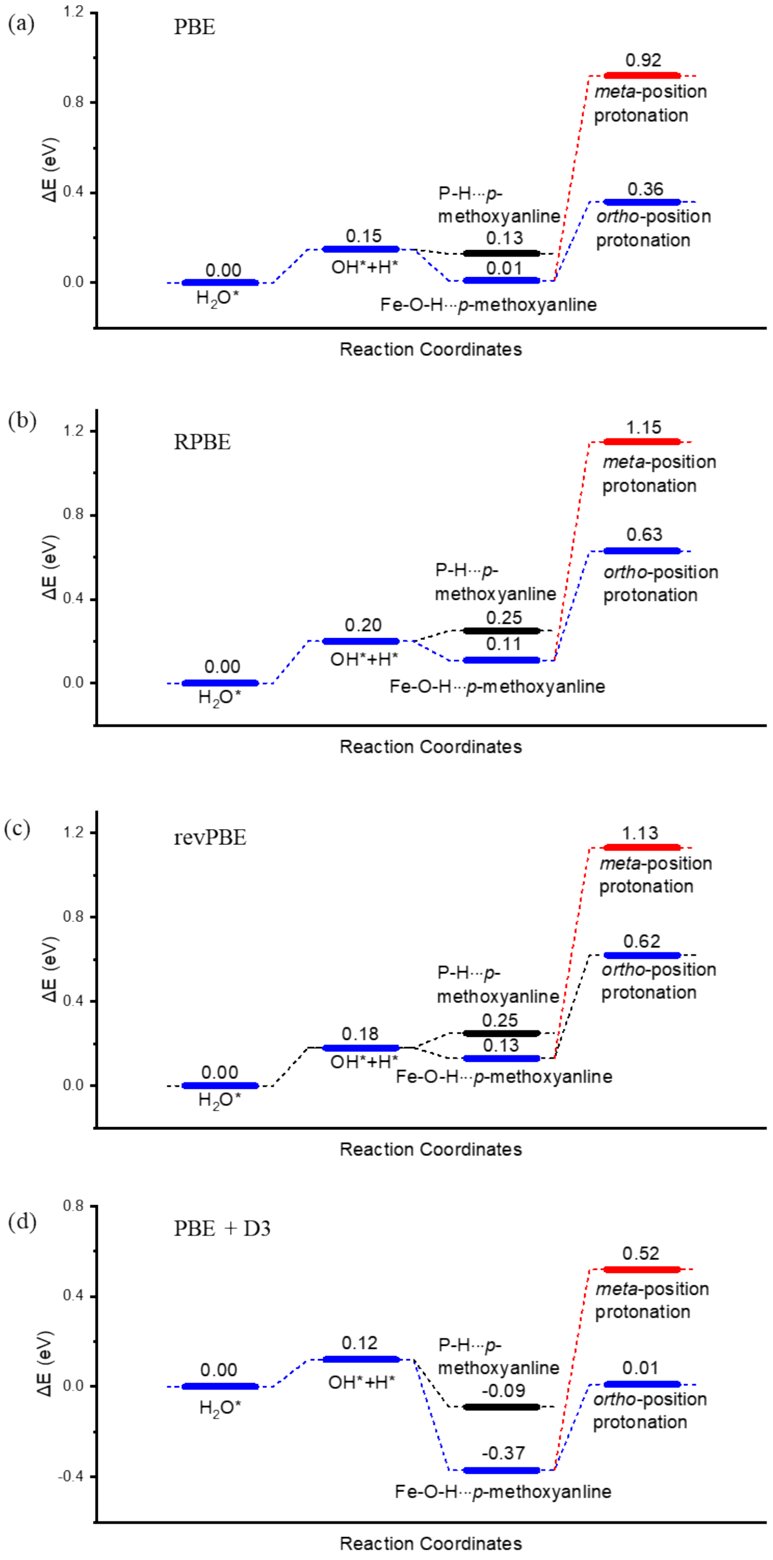

Figure S12. Relative energy profiles (ΔE) of protonation on FeP_1C_3 by Perdew–Burke–Ernzerhof (PBE) functional (a), RPBE functional (b), revPBE functional (c) and PBE+D3 method (d).

To simplify further analysis, the method tests were carried out using H_2O as the model molecule. The potential energy surfaces were re-optimized using four different methods, including Perdew–Burke–Ernzerhof (PBE), RPBE, revPBE, and PBE including van der Waals dispersion corrections with the lates parameter (PBE+D3). Energy barriers obtained from RPBE and revPBE are slightly higher (0.03 – 0.27 eV) than those from PBE, while the PBE+D3 functional considerably strengthens van der Waals interactions between the phenyl ring and the substrate (by 0.22 – 0.40 eV). The results indicate that the choice of functional does not significantly impact the reaction direction.

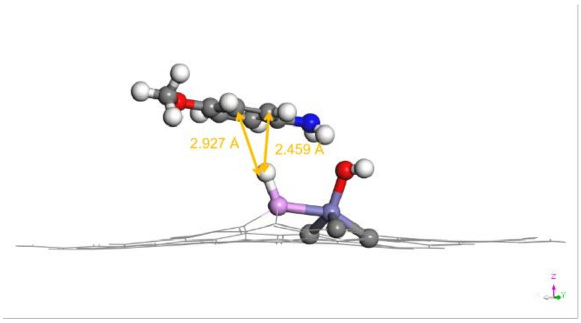

Figure S13. Spatial distance of H species on P-D* with *ortho* and *meta* position of *p*-methoxyaniline.

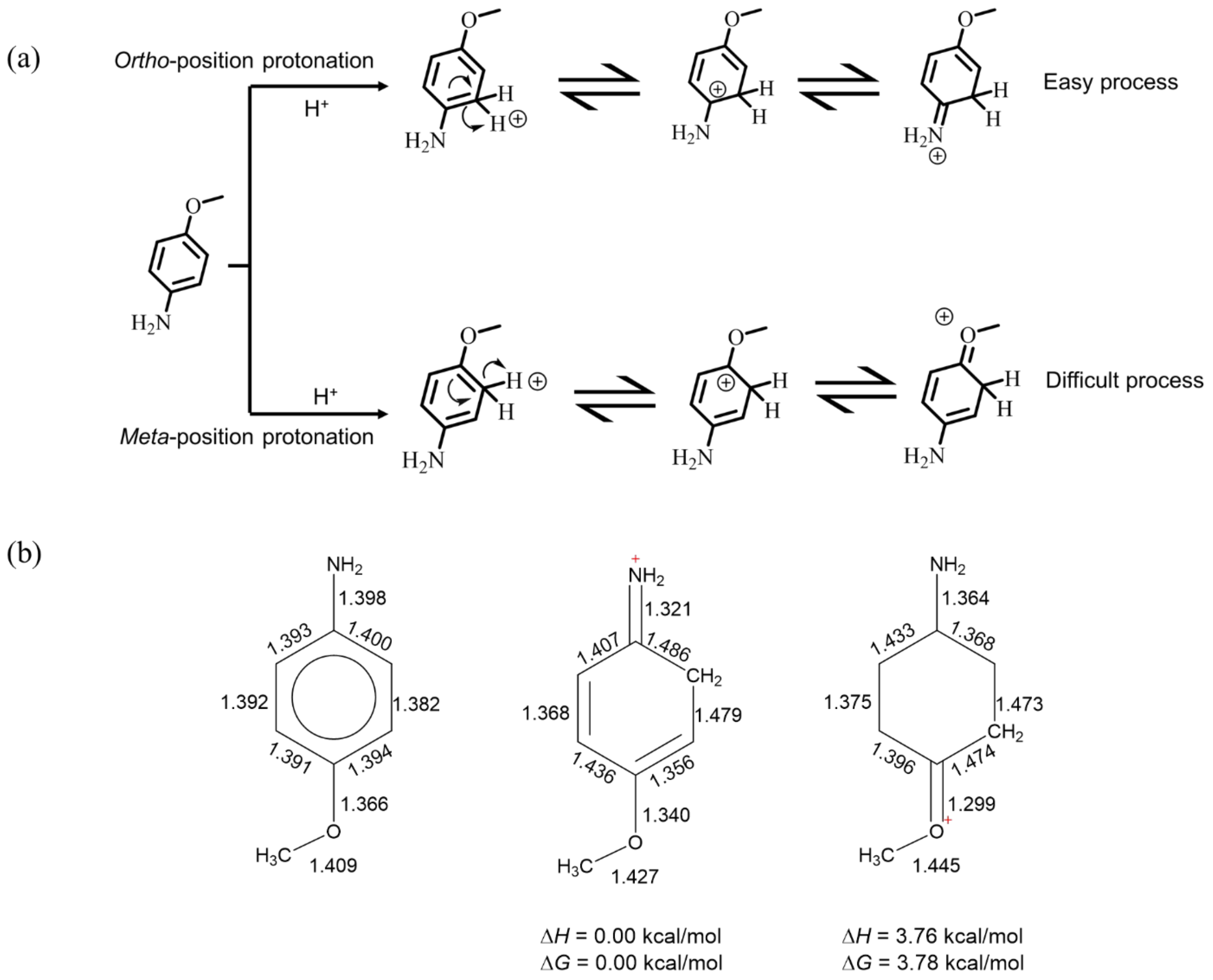

Figure S14. (a) *Ortho*- and *meta*-position protonation of *p*-methoxyaniline as well as (b) computed bond lengths and relative energy (B3PW91/TZVP).

B3PW961/TZVP computations show that the protonation at the *ortho*- and *meta*-position results stronger C-N bond shortening than C-O bond shortening (0.077 *vs*. 0.067 Å) on one hand, and on the other hand, the *ortho*-protonation is more favored thermodynamically than the *meta*-protonation by 3.78 kcal/mol in Gibbs free energy at room temperature, and this favors the *ortho*-protonation of over 99%.

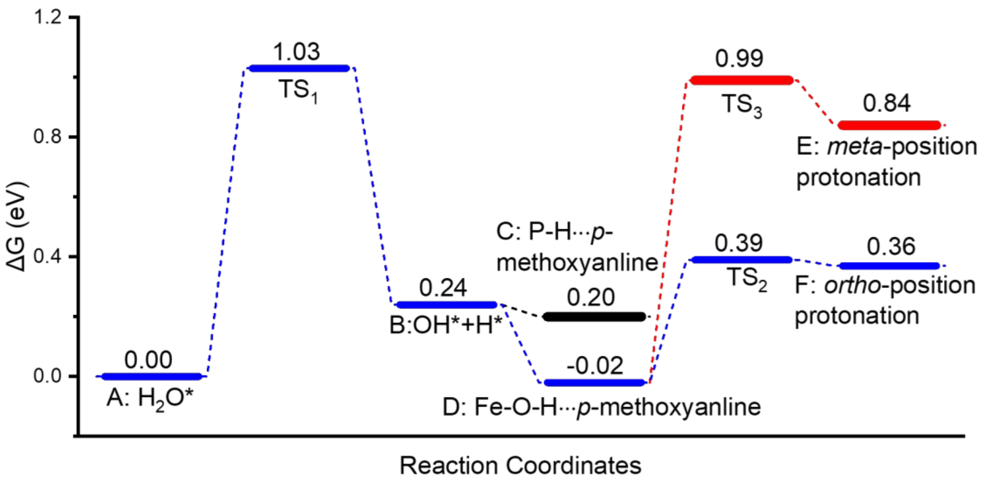

Figure S15. Relative Gibbs free energy (ΔG) profiles of protonation on FeP_1C_3.

Compared with Figure 3, the dissociation of H_2O has a lower Gibbs free energy barrier (1.03 vs. 1.10 eV), which is still the rate-determining step of the overall reaction. As a result, the isotopic substitution of H with D does not affect the overall reaction trend or the rate-determining step.

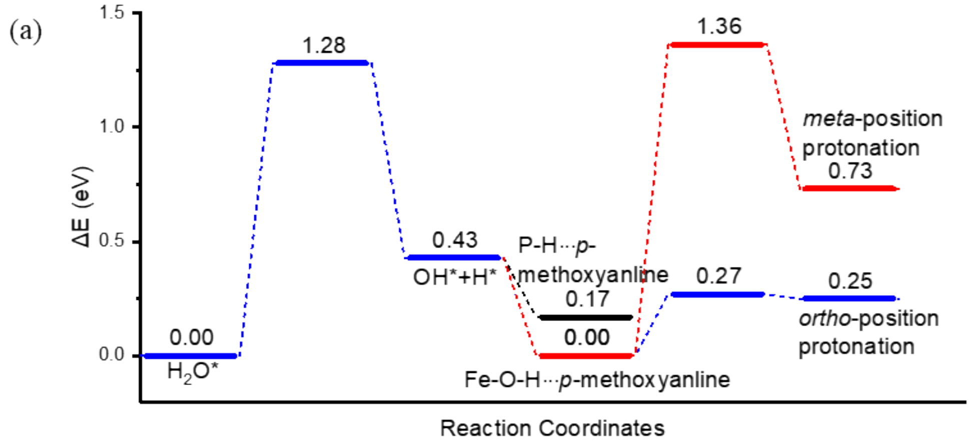

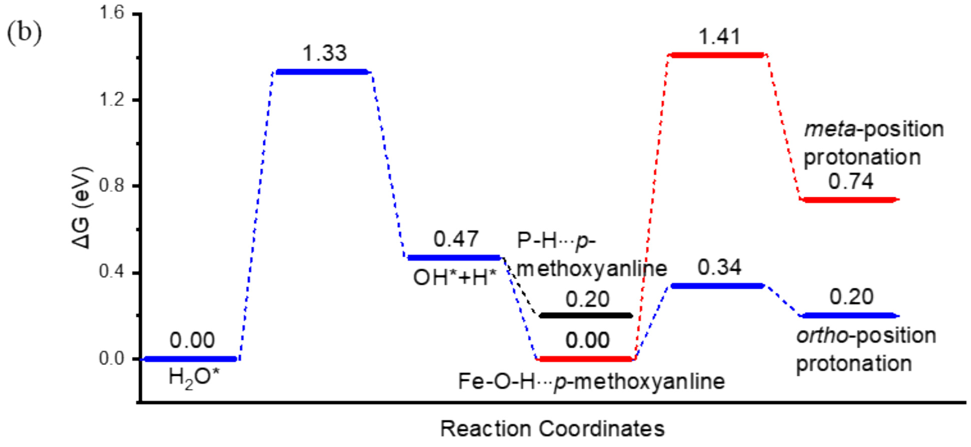

Figure S16. Relative energy profiles (a, Δ*E*) and Gibbs free energy (b, Δ*G*) profiles of deuteration on FeP_1C_3 by PBE+D3 functional combined with an implicit solvent model.

Furthermore, to assess the effects of solvation and weak intermolecular forces, the energy profile was recalculated using PBE+D3 combined with an implicit solvent model. The results consistently identify H_2O dissociation as the rate-determining step, confirming that neither solvation nor dispersion interactions alter the reaction trend.

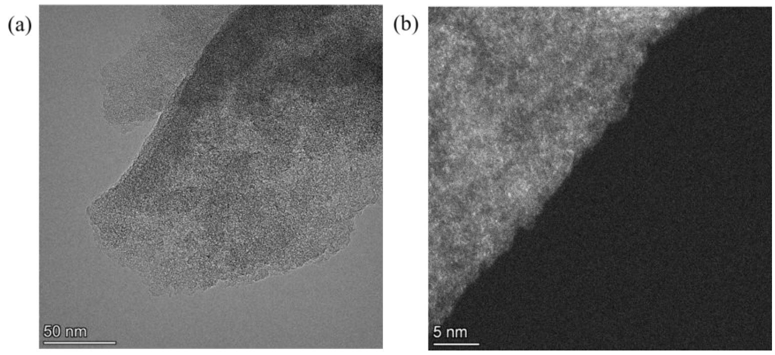

Figure S17. The (a) STEM and (b) HAADF STEM images of scale-up synthetic Fe-P-C catalyst.

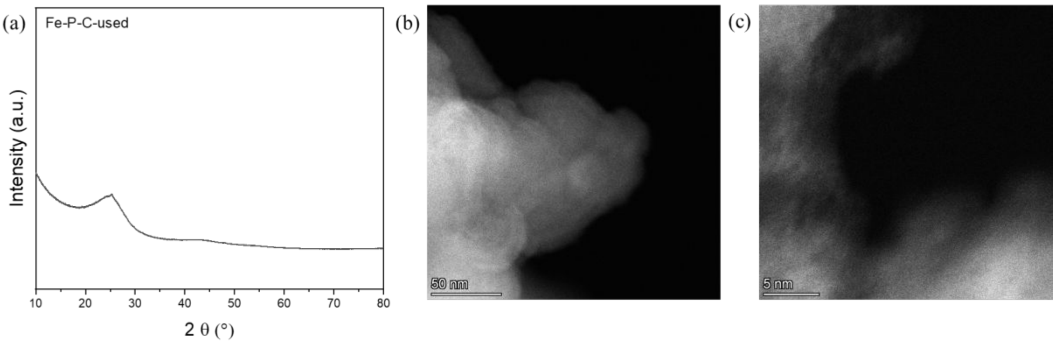

Figure S18. (a) XRD and (b-c) TEM images of Fe-P-C-used catalyst.

Table S1. Catalytic performance of Fe-P-C single-atom catalysts as well as other references.[a]

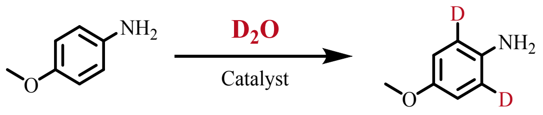

Entry	Catalysts	Deuterium incorporation (%)
		**1b**
1	Fe-N-C-700	< 5
2	Fe-N-C-900	< 5
3	Fe-S-C-700	46
4	Fe-S-C-900	69
5	Cu-P-C-700	21
6	Cu-P-C-900	59
7	Fe powder	< 1
8	Ru/C	< 1
9	Pd/C	< 1
10	Pt/C	< 1

[a] Reaction conditions: 0.5 mmol **1a**, 30 mg catalyst, 1.5 mL D_2O, 2 MPa H_2, 120 ^{o}C, 12 h.

Table S2. The Fe loading determined by ICP-AES analysis in Fe-P-C-800 sample.

Entry	Analytical method	Weight loading (%)
		Fe
1	ICP	0.15

Table S3. The content of P element determined by XPS analysis in Fe-P-C and P-C samples.

Entry	Method	Atomic ratio (at%)
		P
1	Fe-P-C	3.2
2	P-C	1.9

Table S4. The best-fitted EXAFS results of Fe-P-C sample.[a]

Sample	Shell	CN	R(Å)	σ^2 (10^{-2} Å^2)	ΔE_0 (eV)	r-factor (%)
Fe foil	Fe-Fe_{short}	8	2.46	-	-	-
	Fe-Fe_{long}	6	2.84	-	-	
Fe-P-C	Fe-C	2.9 ± 0.4	1.92 ± 0.03	0.8 ± 0.2	-16.9 ± 3.9	0.3
	Fe-P	1.1 ± 0.2	2.26 ± 0.03	0.7 ± 0.2	-16.9 ± 3.9	

[a]CN is the coordination number for the absorber-back scatterer pair, R is the average absorber-backscatterer distance, σ^2 is the Debye-Waller factor, and ΔE_0 is the inner potential correction. S_0^2 was fixed to 0.70 as determined from Fe foil fitting. The data range used for data fitting in k-space (Δk) and R-space (ΔR) are 3.0-11.5 Å^{-1} and 1.0-2.1 Å, respectively.

Table S5. The superior performance of heterogeneous Fe-P-C single-atom catalyst compared to the reported homogeneous catalysts and heterogeneous nano-catalysts.

Entry	Catalyst	**Molar ratio metal/Substrate**	Substrate	Solvent	D source	D content	Ref.
**Homogeneous catalysts**							
1	Cycloocta-1,5-Ir (I)	**-**	4-Aminotoluene	DMF	D_2	77	13
2	Phosphine/Carbene-Ir complex	**5 mol%**	Acetophenon	Et_2O	D_2	97	14
3	N-heterocycylic carbene-Ir	**5 mol%**	4-methylbenzenesulfonamide	DCM	D_2	90	15
4	$Pd(OAc)_2$	**20 mol%**	2-Methyl-N-(quinolin-8-yl) Benzamide	-	D_2O	98	16
5	AgOTf	**5 mol%**	Tetrahydroquinoline	$CDCl_3$	D_2O	94	17
6	Pd-dual ligand system	**10 mol%**	Parabens	d_1-HFIP	D_2O	Up to 95	18
7	$Ru_3(CO)_{12}$	**5 mol%**	Isoquinoline	-	t-BuOD	80	19
8	$Ru_3(CO)_{12}$	**0.25 mol%**	N,N-dimethylaniline	Toluene	D_2O	96	20
**Heterogeneous single-atom catalyst**							
9	Fe-P-C	**0.16 mol%**	*p*-anisidine	-	D_2O	98	This work
**Heterogeneous nano-catalysts**							
10	Pd/C	**0.9 mol%**	Aniline	-	D_2O	>97	21
11	Pt/C	**0.5 mol%**	Aniline	-	D_2O	98	21
12	$NaBD_4$-activated Pd	**10 wt%**	2-aminobenzoic acid	-	D_2O, $NaBD_4$	Up to 95	22
13	NHC-stabilized Ir nanoparticles	**4 mol%**	N-Butylaniline	THF	D_2	93	23
14	10wt% Fe-Cellulose-1000	**20 mol%**	*p*-anisidine	-	D_2O	96	12
15	4.8wt% Mn-Starch-1000	**20 mol%**	*p*-anisidine	-	D_2O	94	24

Supplementary References

1. Kresse, G.; Furthmüller, J. Efficiency of ab-initio total energy calculations for metals and semiconductors using a plane-wave basis set. *Comput. Mater. Sci*. **1996**, *6*, 15–50.

2. Kresse, G.; Joubert, D. From ultrasoft pseudopotentials to the projector augmented-wave method. *Phys. Rev. B* **1999**, 59, 1758–1775.

3. Perdew, J. P.; Chevary, J. A.; Vosko, S. H.; Jackson, K. A.; Pederson, M. R.; Singh, D. J.; Fiolhais, C. Atoms, molecules, solids, and surfaces: Applications of the generalized gradient approximation for exchange and correlation. *Phys. Rev. B* **1992**, 46, 6671–6687.

4. Hammer, B.; Hansen, L. B.; Nørskov, J. K. Improved adsorption energetics within density-functional theory using revised Perdew-Burke-Ernzerhof functionals. *Phys. Rev. B* **1999**, 59, 7413.

5. Zhang, Y.; Yang, W. Comment on "Generalized Gradient Approximation Made Simple". *Phys. Rev. Lett*. **1998**, 80, 890.

6. Grimme, S.; Antony, J.; Ehrlich, S.; Krieg, S. A consistent and accurate ab initio parametrization of density functional dispersion correction (DFT-D) for the 94 elements H-Pu. *J. Chem. Phys.* **2010**, 132, 154104.

7. Mathew, K.; Sundararaman, R.; Letchworth-Weaver, K.; Arias, T. A.; Hennig, R. G. Implicit solvation model for density-functional study of nanocrystal surfaces and reaction pathways. *J. Chem. Phys*. **2014**, 140, 084106.

8. Monkhorst, H. J.; Pack, J. D. Special points for Brillouin-zone integrations. *Phys. Rev. B* **1976**, 13, 5188–5192.

9. Jónsson, H.; Mills, G.; Jacobsen, K.W. Nudged elastic band method for finding minimum energy paths of transitions, in: B.J. Berne, G. Ciccotti, D.F. Coker (Eds.) Classical and Quantum Dynamics in Condensed Phase Simulations, World Scientific, Hackensack, **1998**.

10. Henkelman, G.; Jonsson, H. A dimer method for finding saddle points on high dimensional potential surfaces using only first derivatives, *J. Chem. Phys.* **1999**, 111, 7010-7022.

11. Wang V.; Xu, N.; Liu, J. C.; Tang, G.; Geng, W. T. VASPKIT: A user-friendly interface facilitating high-throughput computing and analysis using VASP code. *Comput. Phys. Commun.*, **2021**, 267, 108033.

12. Li, W. et. al. Scalable and selective deuteration of (hetero)arenes. *Nat. Chem.* **14**, 334–341 (2022).

13. J. Hickey, M. et. al. Iridium-catalysed labelling of anilines, benzylamines and nitrogen heterocycles using deuterium gas and cycloocta-1,5-dienyliridium(I) 1,1,1,5,5,5-hexafluoropentane-2,4-dionate. *Tetrahedron Letters*, **44**, 3959–3961 (2003).
14. R. Cochrane, A. et. al. Practically convenient and industrially-aligned methods for iridium-catalysed hydrogen isotope exchange processes. *Org. Biomol. Chem.* **12**, 3598 (2014).
15. J. Kerr, Wi.; Reid, M.; Tuttle, T. Iridium-Catalyzed C−H Activation and Deuteration of Primary Sulfonamides: An Experimental and Computational Study. *ACS Catal.* **5**, 402−410 (2015).
16. Yu, Y. et. al. Palladium-Catalyzed H/D Exchange Reaction with 8-Aminoquinoline as the Directing Group: Access to ortho-Selective Deuterated Aromatic Acids and β-Selective Deuterated Aliphatic *Acids. J. Org. Chem.* **83**, 7860−7866 (2018).
17. Hao, N.; Cong, X.; Dong, B. Silver-catalyzed regioselective deuteration of (hetero)arenes and a-deuteration of 2-alkyl azaarenes. *RSC Adv.*, **10**, 25475 (2020).
18. Farizyan, M.; Mondal, A.; Mal, S.; Deufel, F.; Gemmeren, M. Palladium-Catalyzed Nondirected Late-Stage C−H Deuteration of Arenes. *J. Am. Chem. Soc.* **143**, 16370−16376 (2021).
19. Gröll, B.; Schnü rch, M.; D. Mihovilovic, M. Selective Ru(0)-Catalyzed Deuteration of Electron-Rich and ElectronPoor Nitrogen-Containing Heterocycles. *J. Org. Chem.* **77**, 4432−4437 (2012).
20. Zhan, M. et. al. A convenient method for the Ru(0)-catalyzed regioselective deuteration of N-alkyl-substituted anilines. *Tetrahedron Letters,* **55**, 5070–5073 (2014).
21. Sajiki, H. et. al. Aromatic ring favorable and efficient H–D exchange reaction catalyzed by Pt/C. Tetrahedron Letters, **46**, 6995–6998 (2005).
22. Derdau, V.; Atzrodt, J.; Zimmermann, J.; Kroll, C.; Brückner, F. Hydrogen–Deuterium Exchange Reactions of Aromatic Compounds and Heterocycles by $NaBD_4$-Activated Rhodium, Platinum and Palladium Catalysts. *Chem. Eur. J.* **15**, 10397–10404 (2009).
23. Valero, M. et. al. NHC-Stabilized Iridium Nanoparticles as Catalysts in Hydrogen Isotope Exchange Reactions of Anilines. *Angew. Chem. Int. Ed.* **59**, 3517 –3522 (2020). *Angew. Chem.* **132**, 3545–3550 (2020).
24. Bourriquen, F.; Rockstroh, N.; Bartling, S.; Junge, K.; Beller, M. Manganese-Catalysed Deuterium Labelling of Anilines and ElectronRich (Hetero)Arenes. *Angew. Chem. Int. Ed.* **61**, e202202423 (2022). *Angew. Chem.* **134**, e202202423 (2022).

NMR Spectra

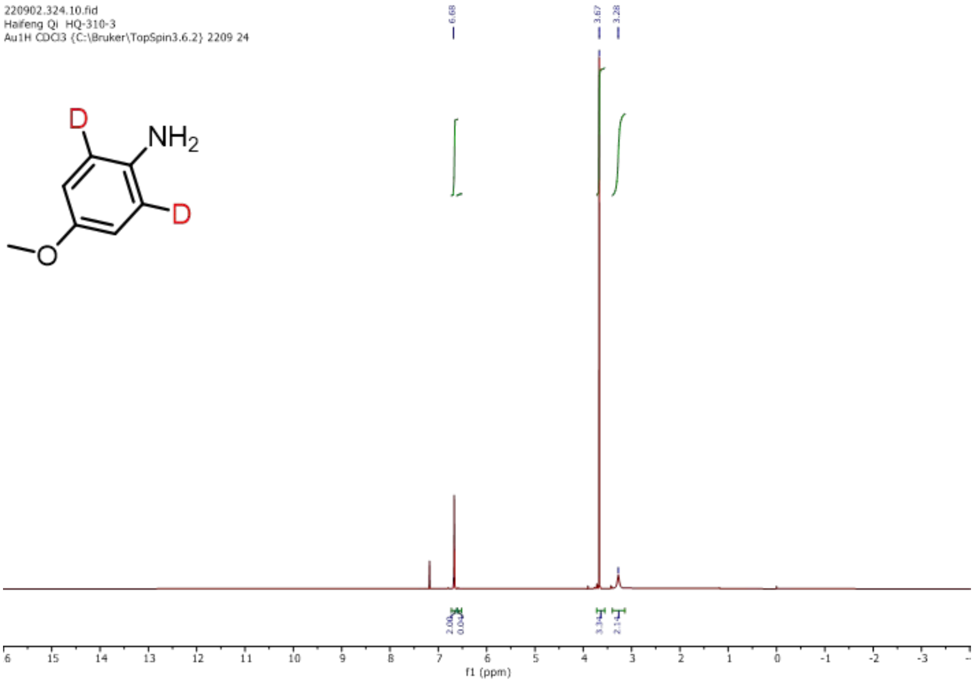
220902.324.10.fid
Haifeng Qi HQ-310-3
Au1H CDCl3 {C:\Bruker\TopSpin3.6.2} 2209 24
6.68
3.67
3.28
D
NH2
D
O
2.00
0.04
3.34
2.14
f1 (ppm)

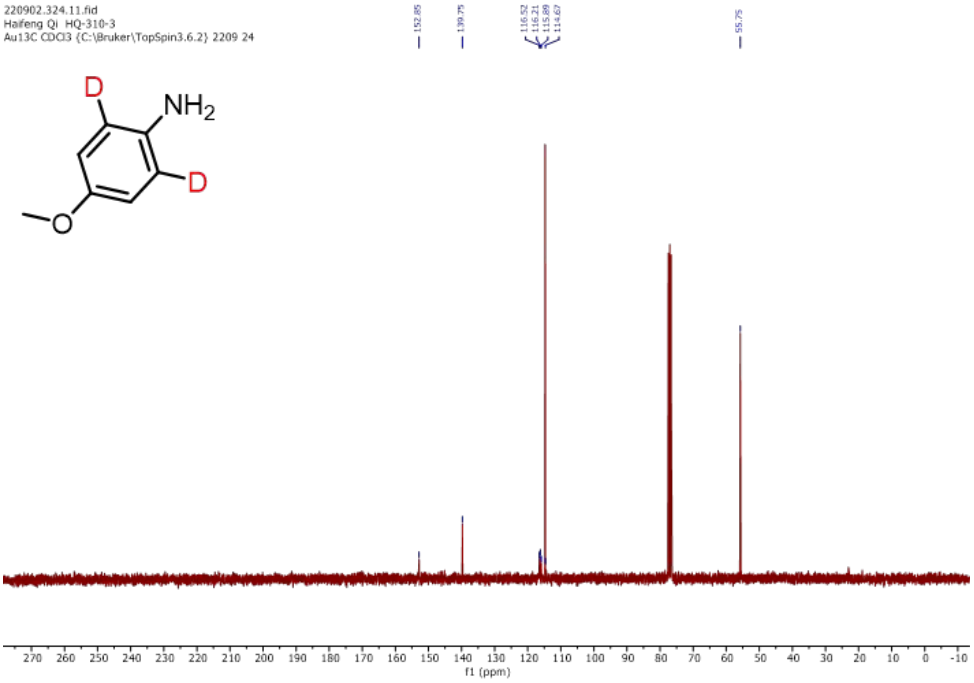
220902.324.11.fid
Haifeng Qi HQ-310-3
Au13C CDCl3 {C:\Bruker\TopSpin3.6.2} 2209 24
152.85
139.75
116.52
116.21
115.89
114.67
55.75
D
NH2
D
O
f1 (ppm)

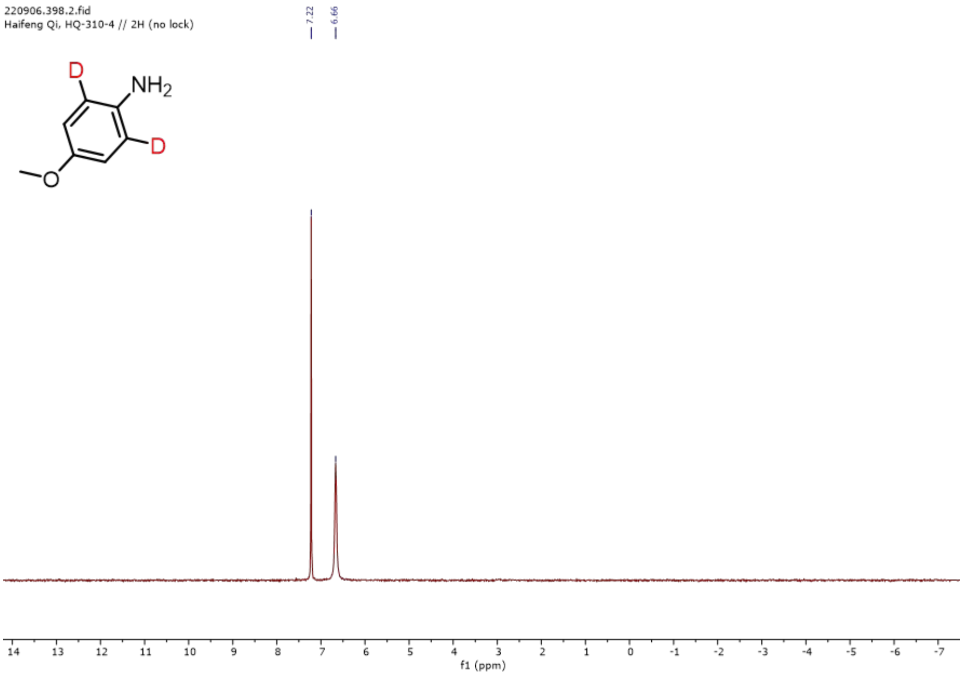

Figure S19. ^{1}H, ^{13}C NMR and ^{2}H spectra of deuterated product **1c**.

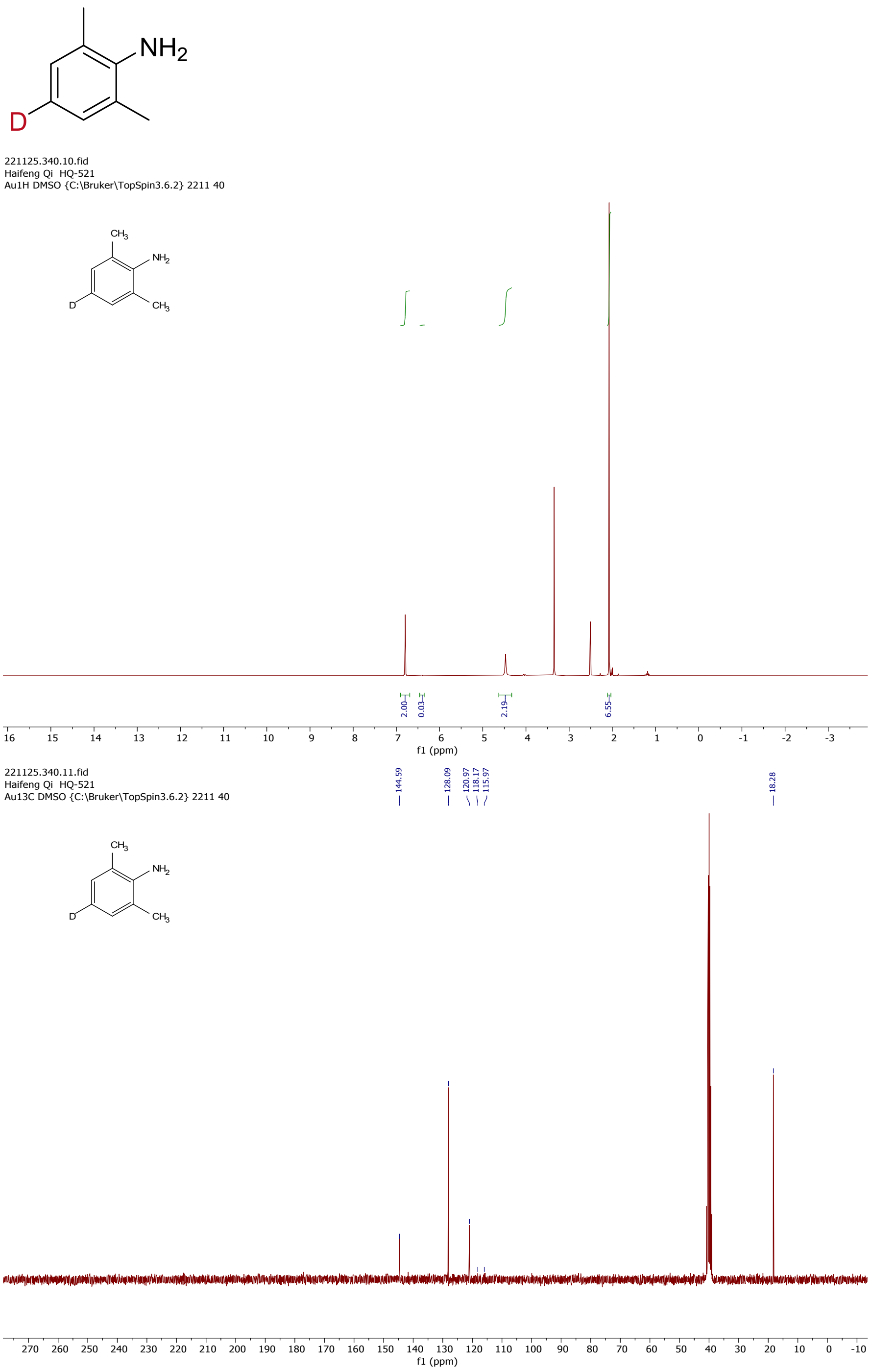

Figure S20. ^{1}H and ^{13}C NMR spectra of deuterated product **2b**.

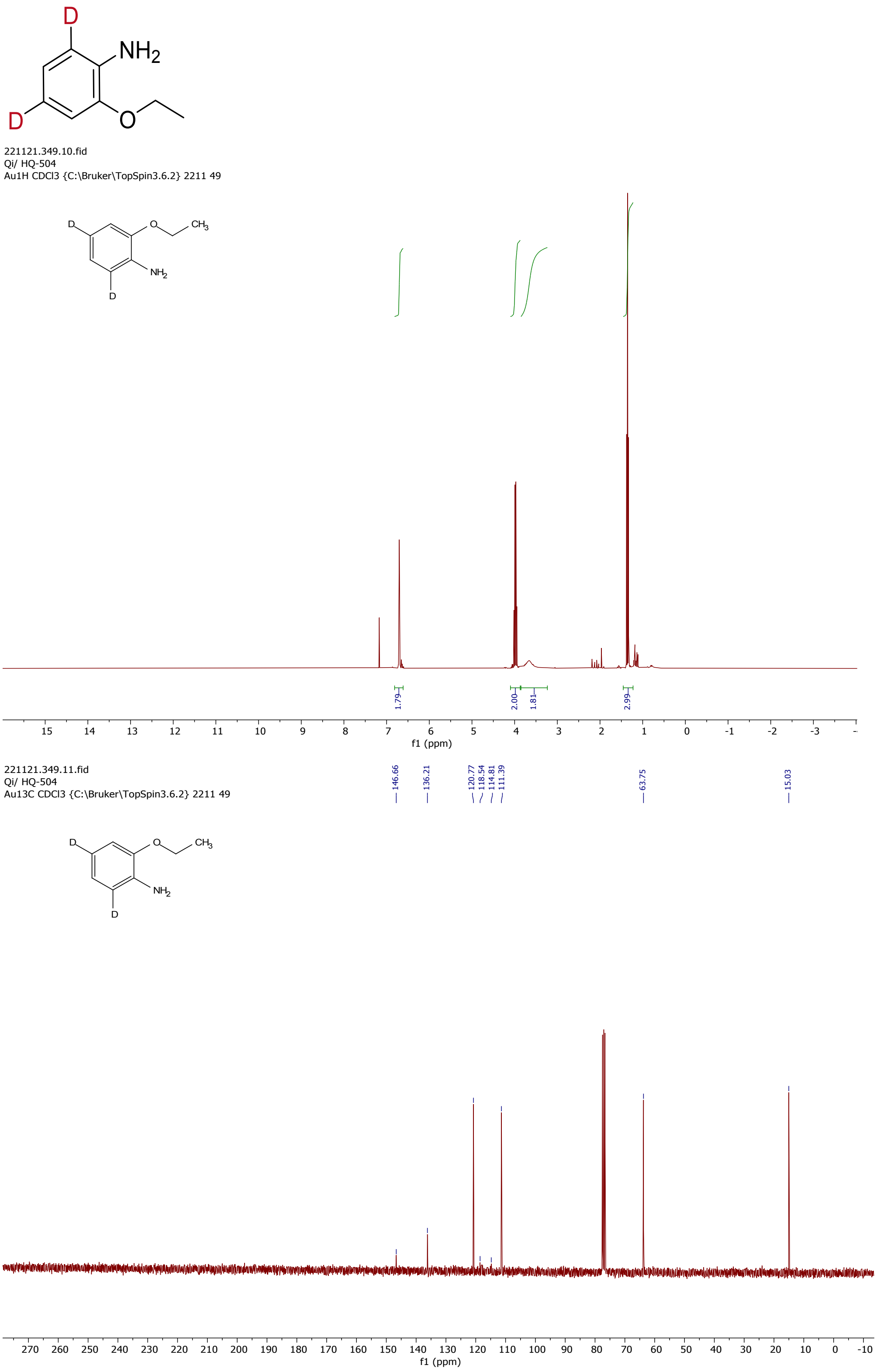

Figure S21. ^{1}H and ^{13}C NMR spectra of deuterated product **3b**.

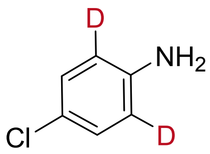

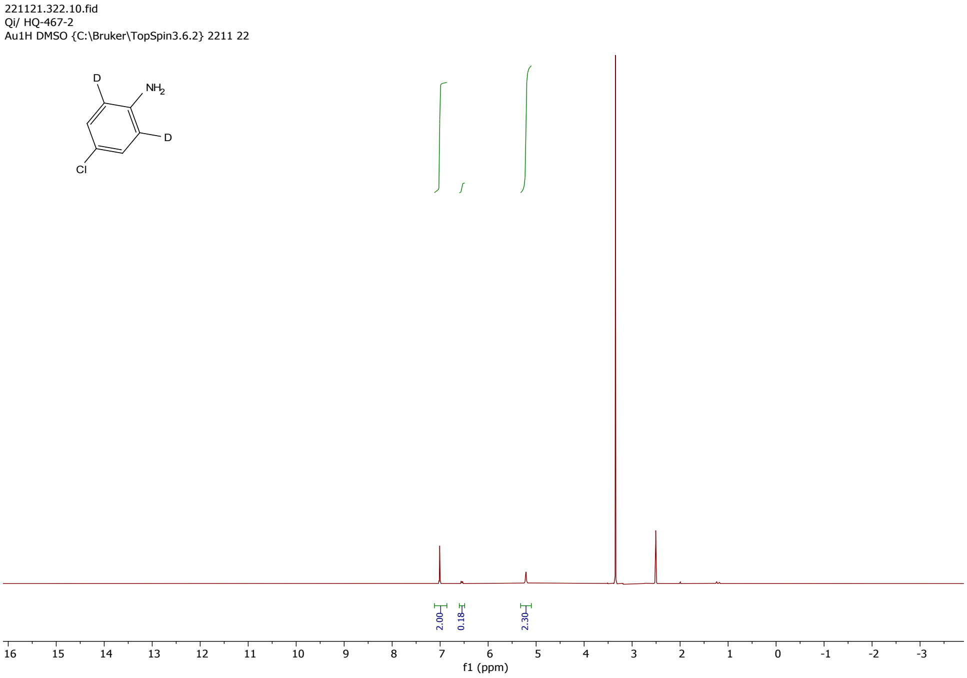

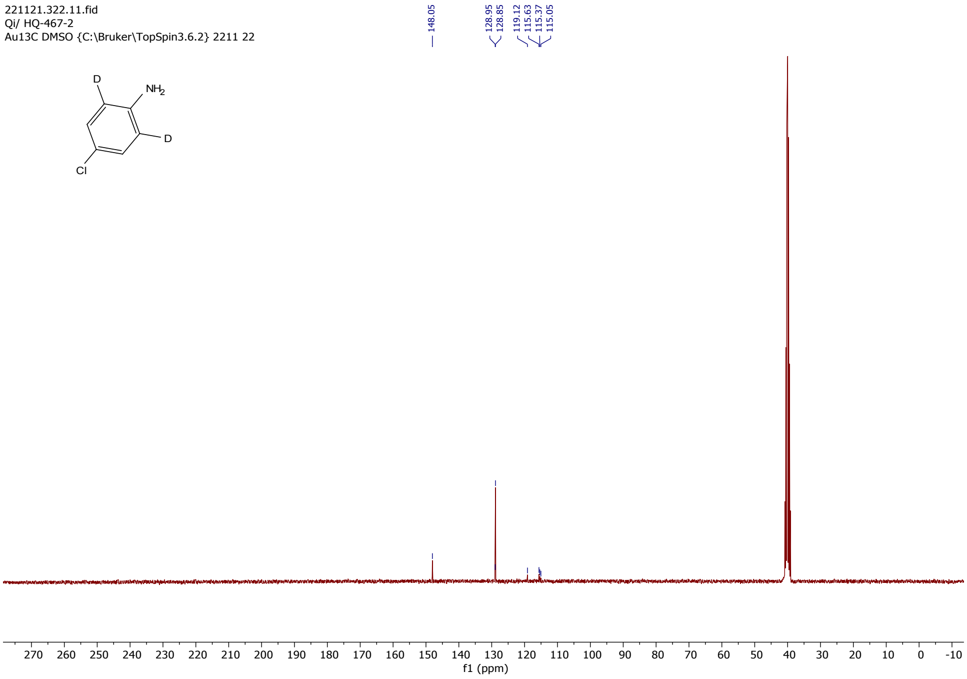

Figure S22. ^{1}H and ^{13}C NMR spectra of deuterated product **4b**.

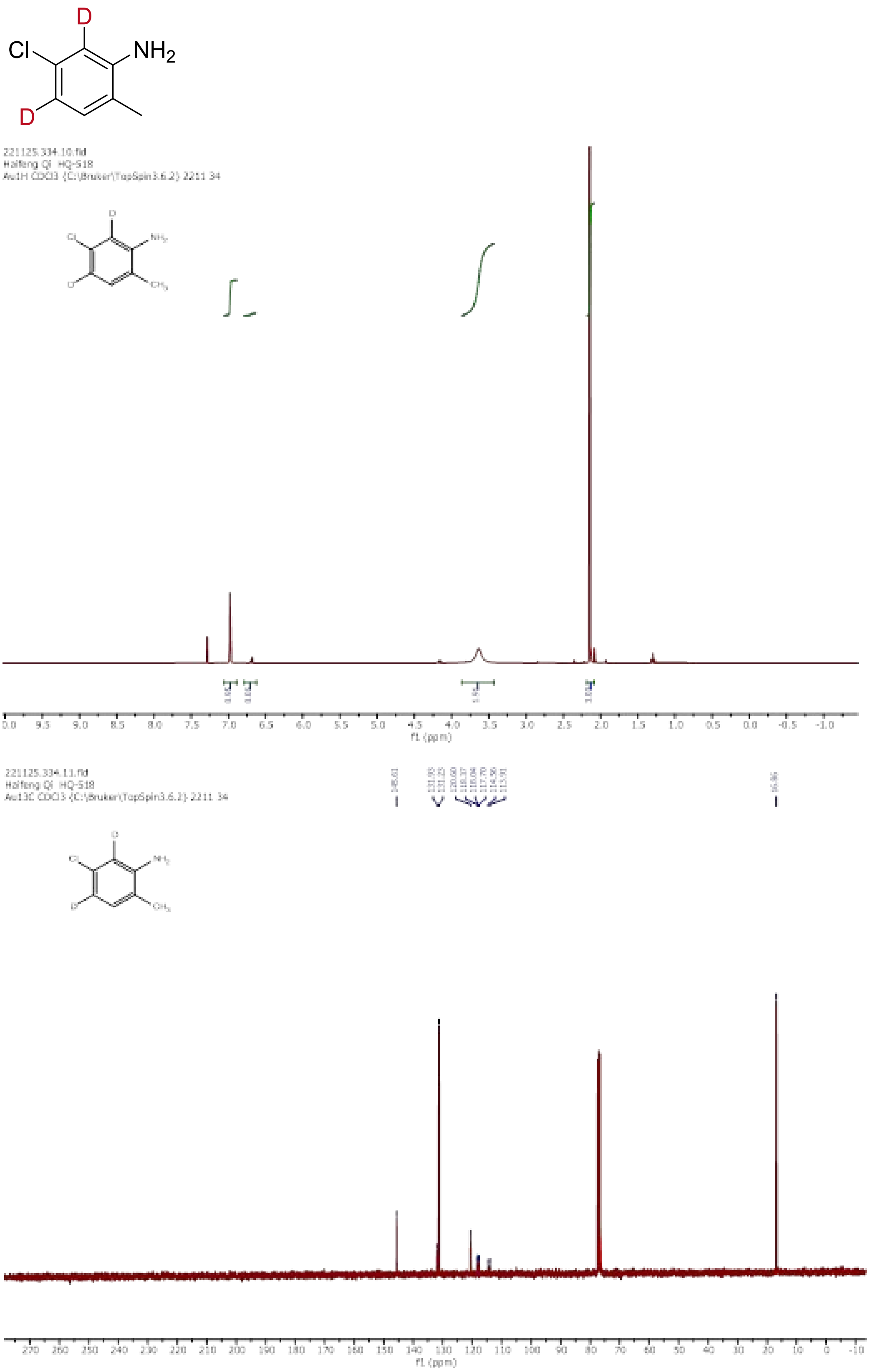

Figure S23. ^{1}H and ^{13}C NMR spectra of deuterated product **5b**.

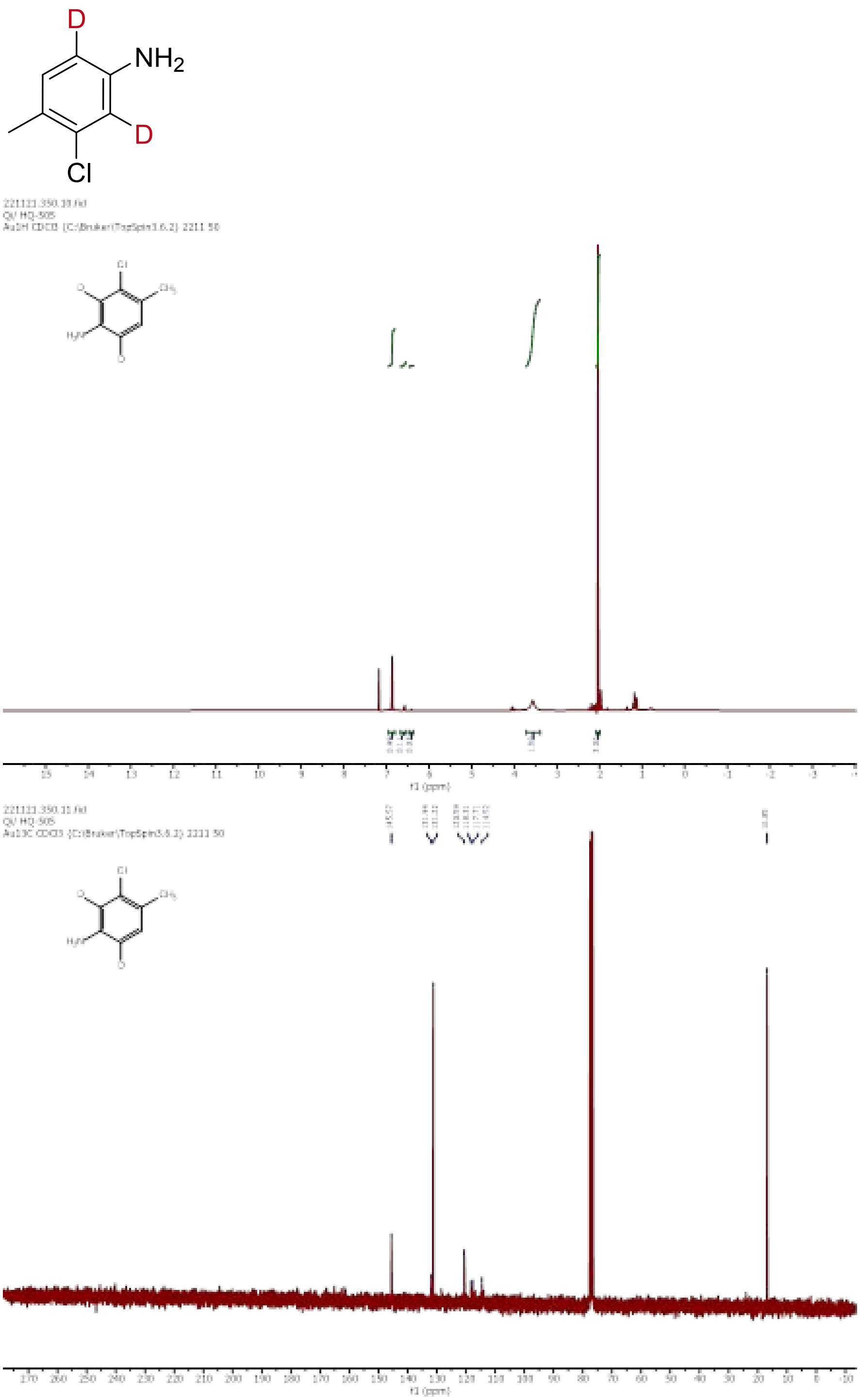

Figure S24. ^{1}H and ^{13}C NMR spectra of deuterated product **6b**.

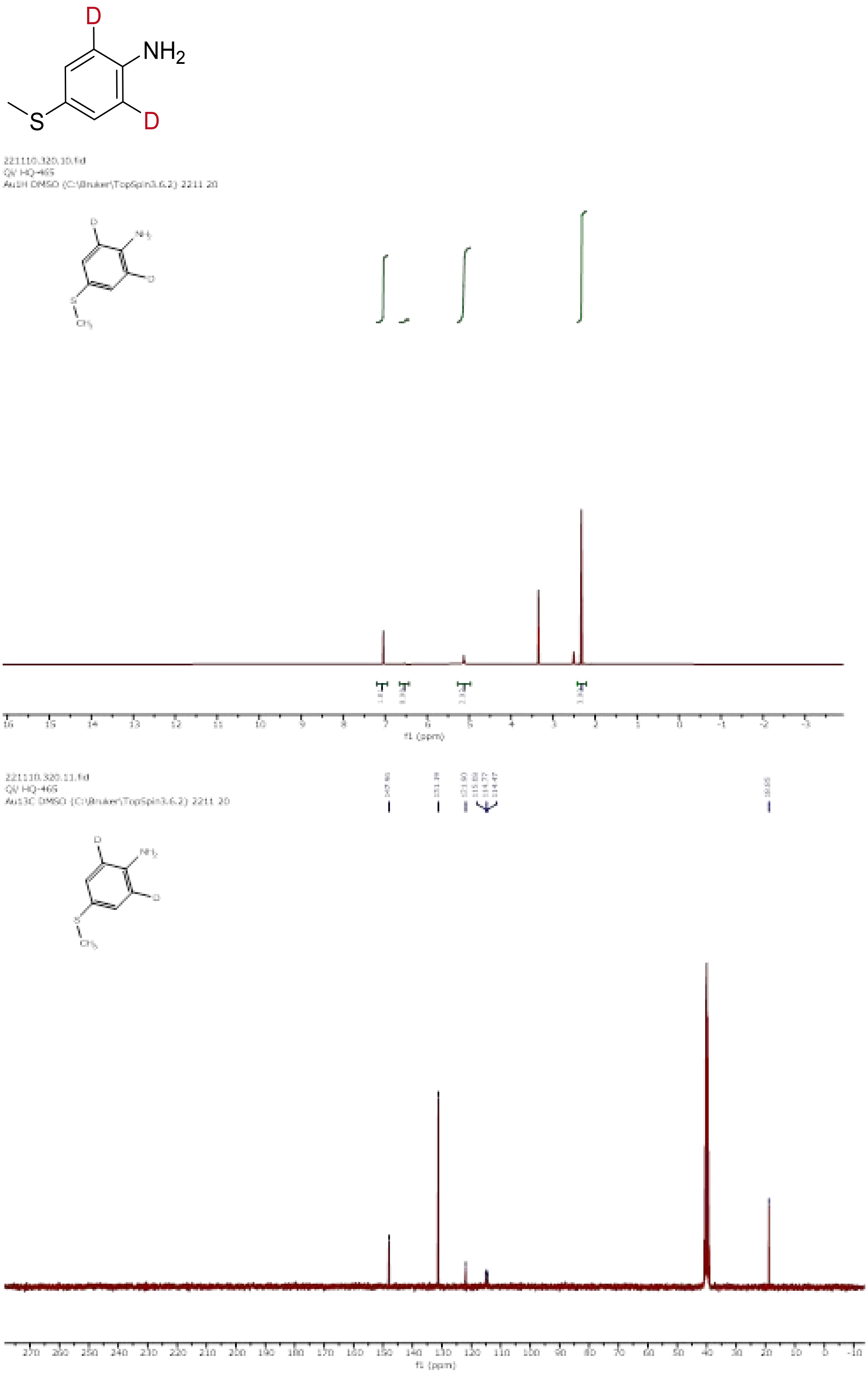

Figure S25. ^{1}H and ^{13}C NMR spectra of deuterated product **7b**.

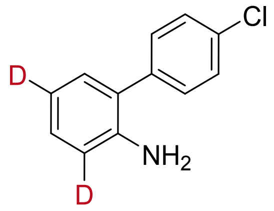

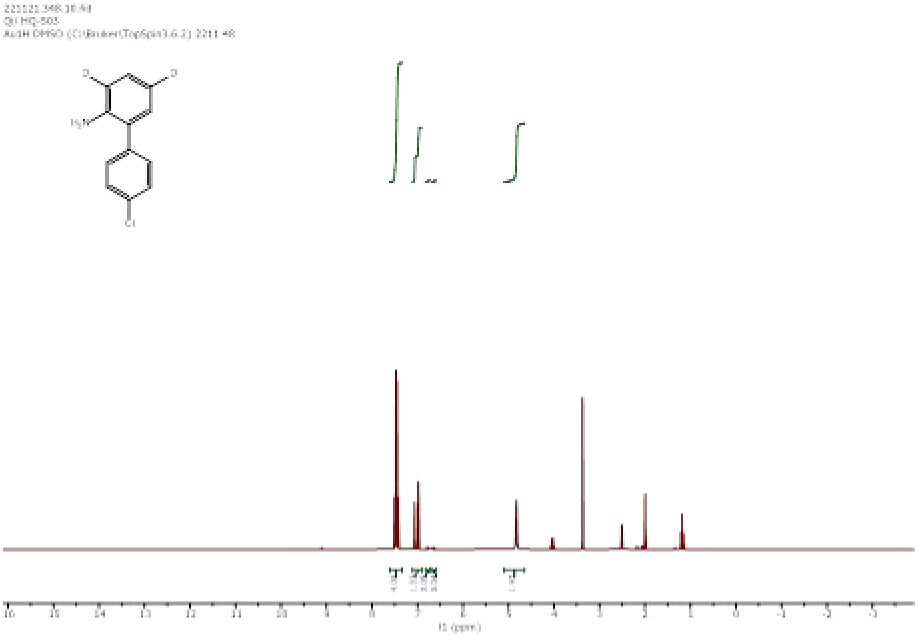

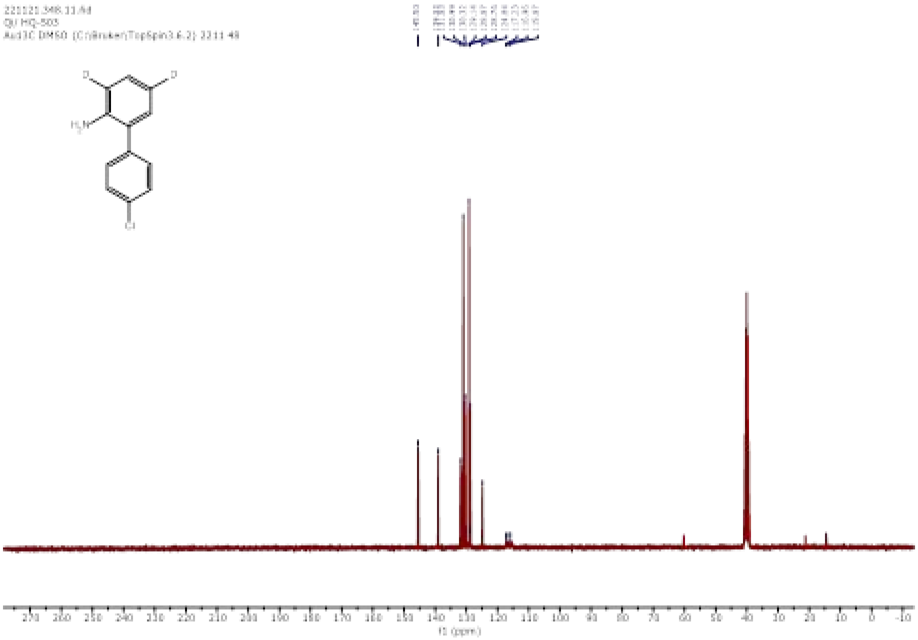

Figure S26. ^{1}H and ^{13}C NMR spectra of deuterated product **8b**.

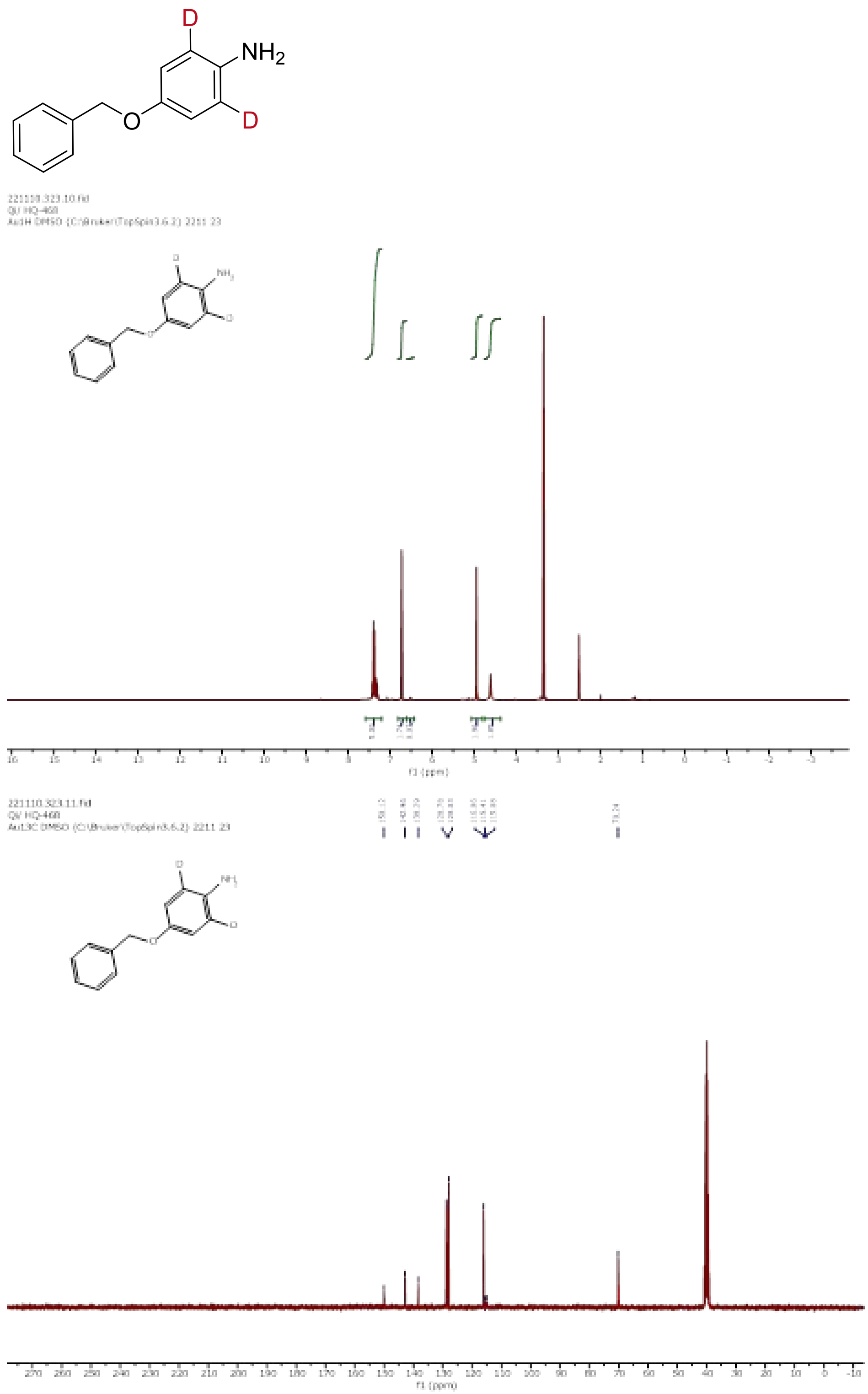

Figure S27. ^{1}H and ^{13}C NMR spectra of deuterated product **9b**.

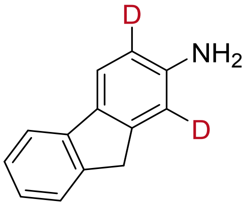

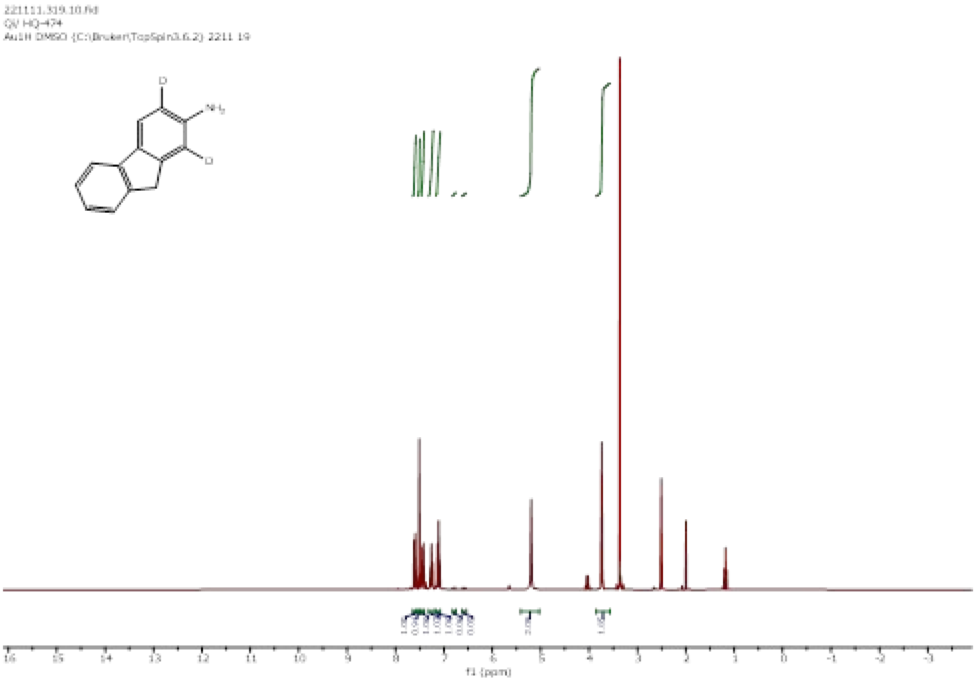

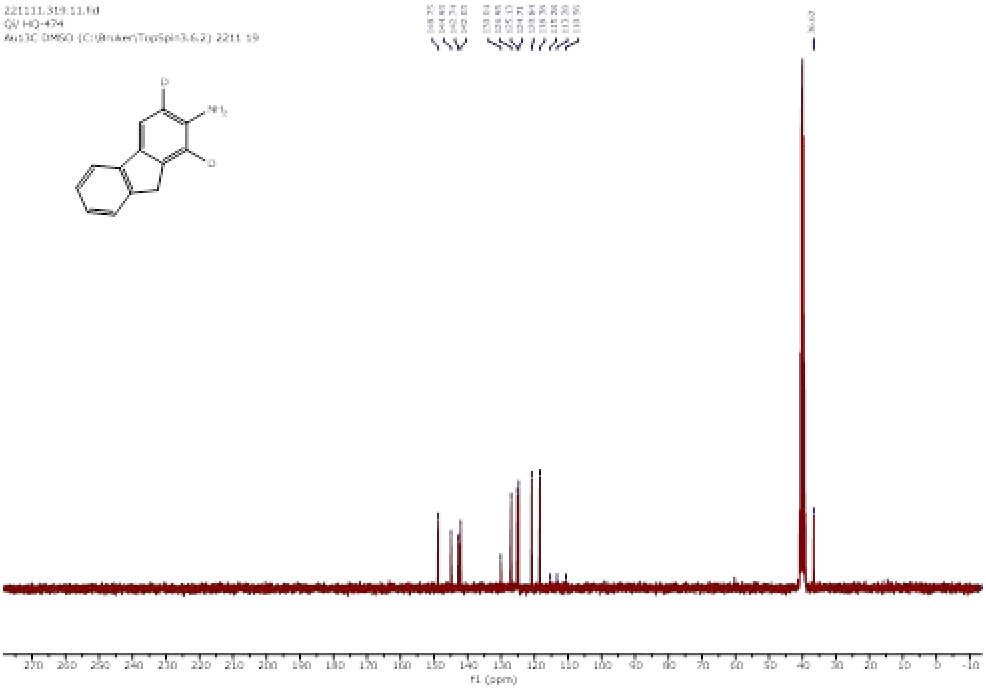

Figure S28. ^{1}H and ^{13}C NMR spectra of deuterated product **10b**.

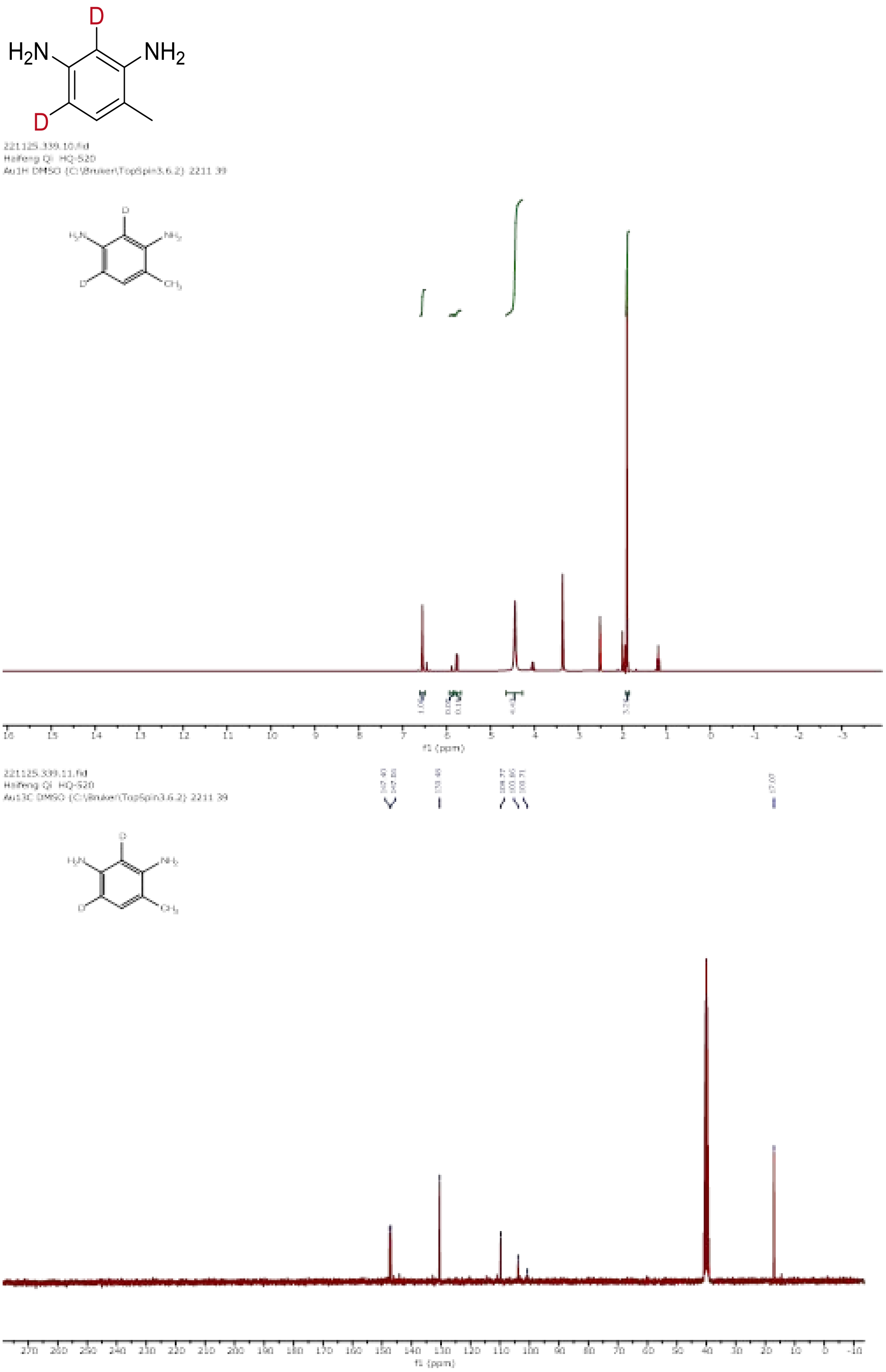

Figure S29. ^{1}H and ^{13}C NMR spectra of deuterated product **11b**.

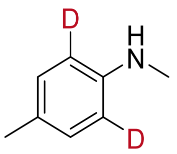

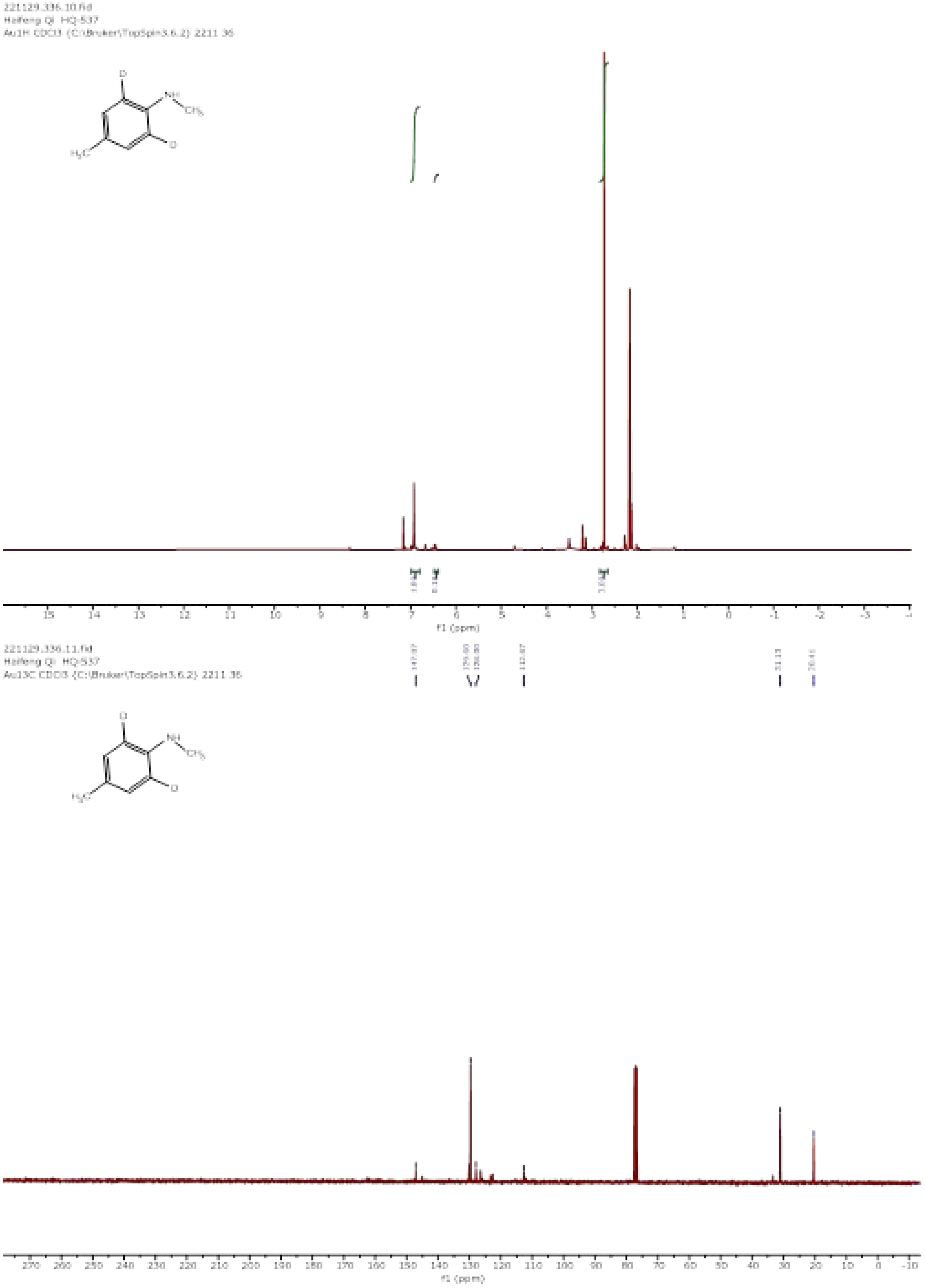

Figure S30. ^{1}H and ^{13}C NMR spectra of deuterated product **12b**.

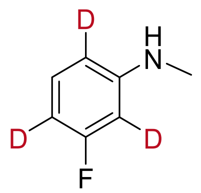

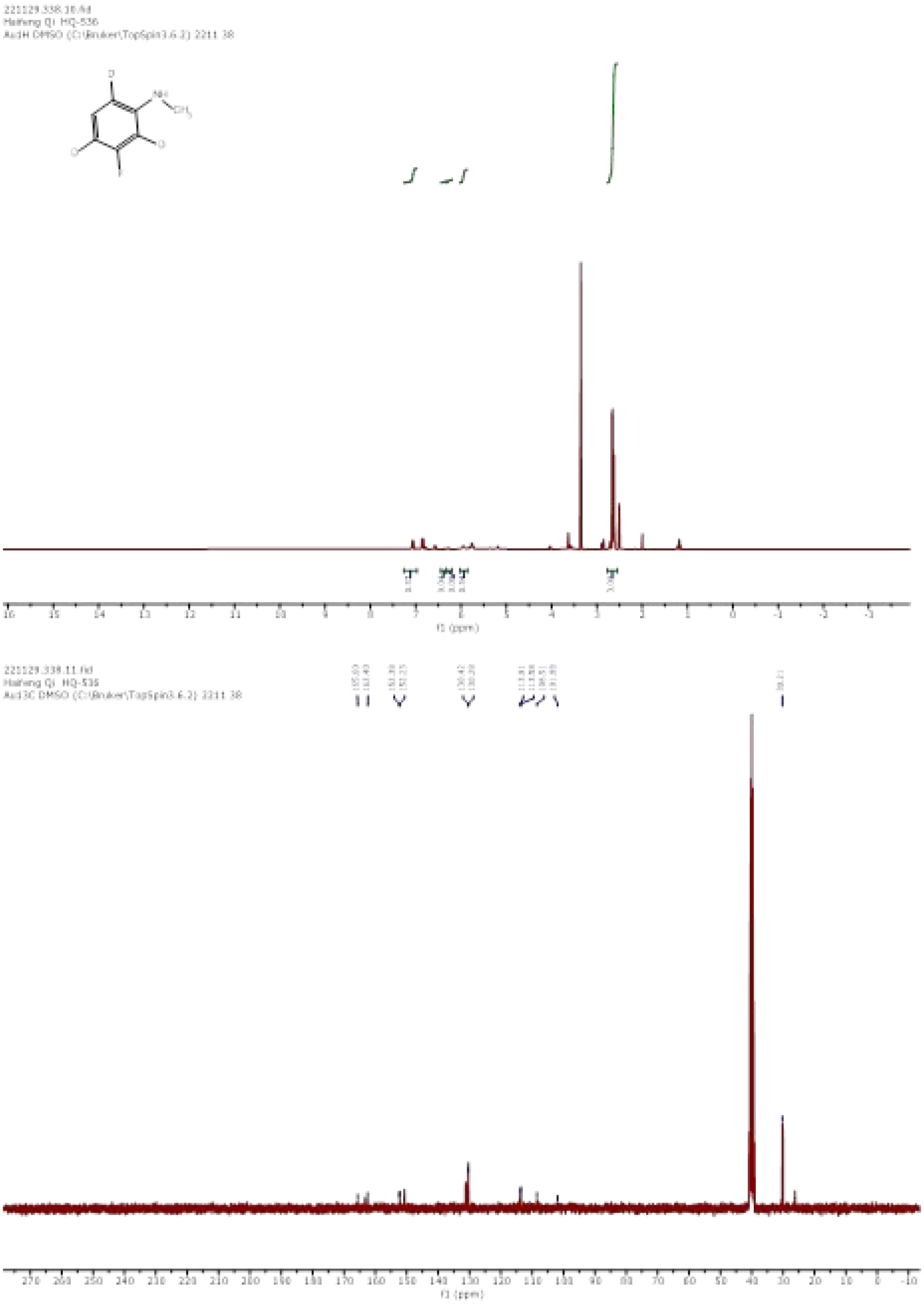

Figure S31. ^{1}H and ^{13}C NMR spectra of deuterated product **13b**.

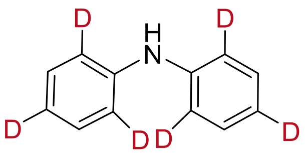

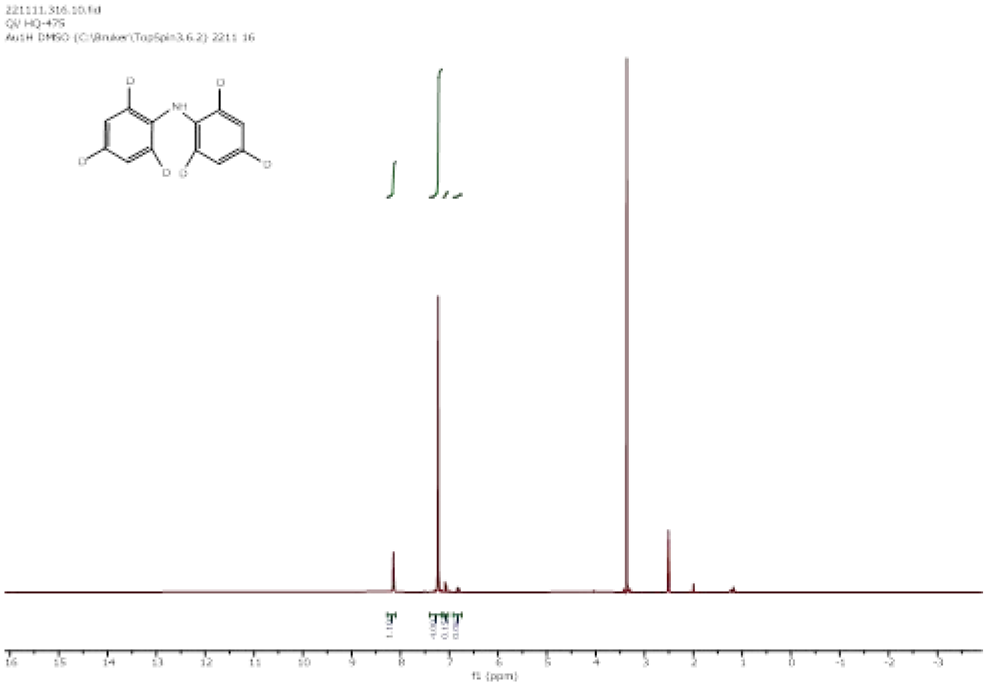

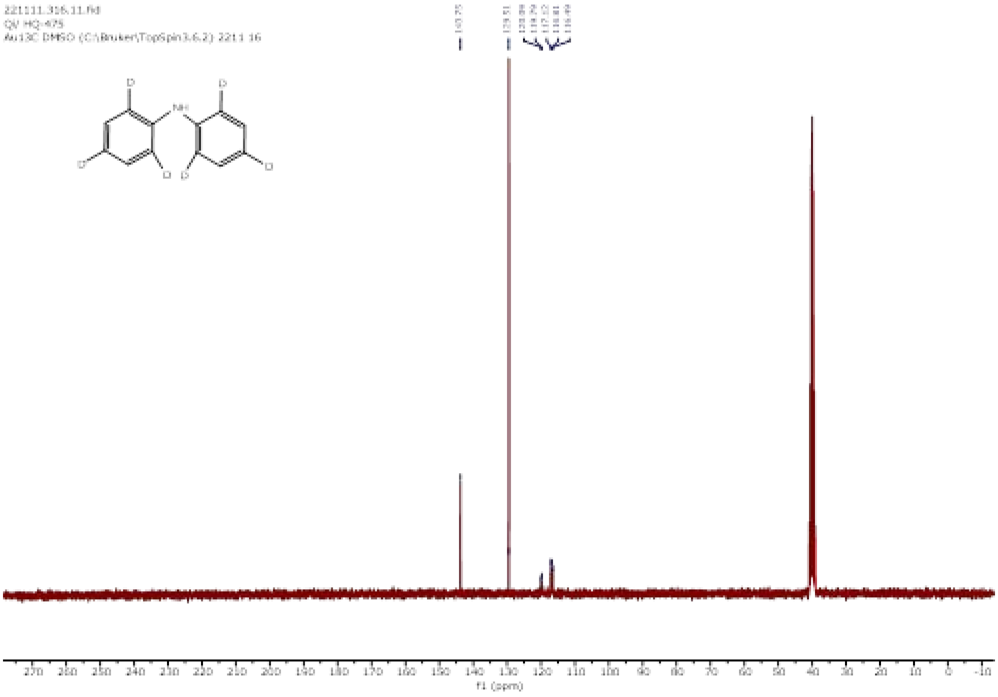

Figure S32. ^{1}H and ^{13}C NMR spectra of deuterated product **14b**.

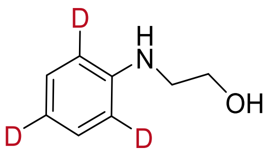

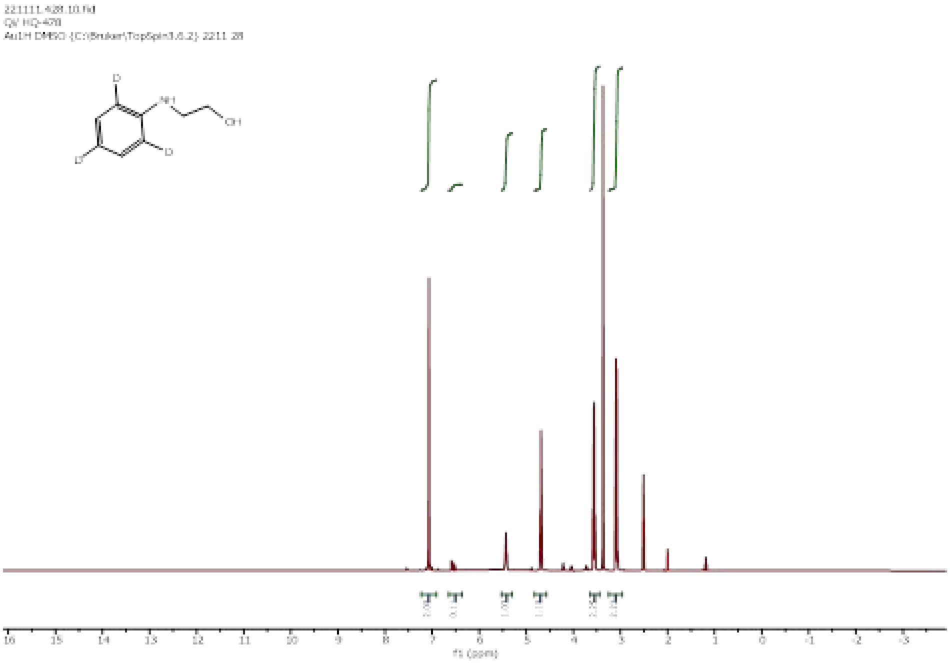

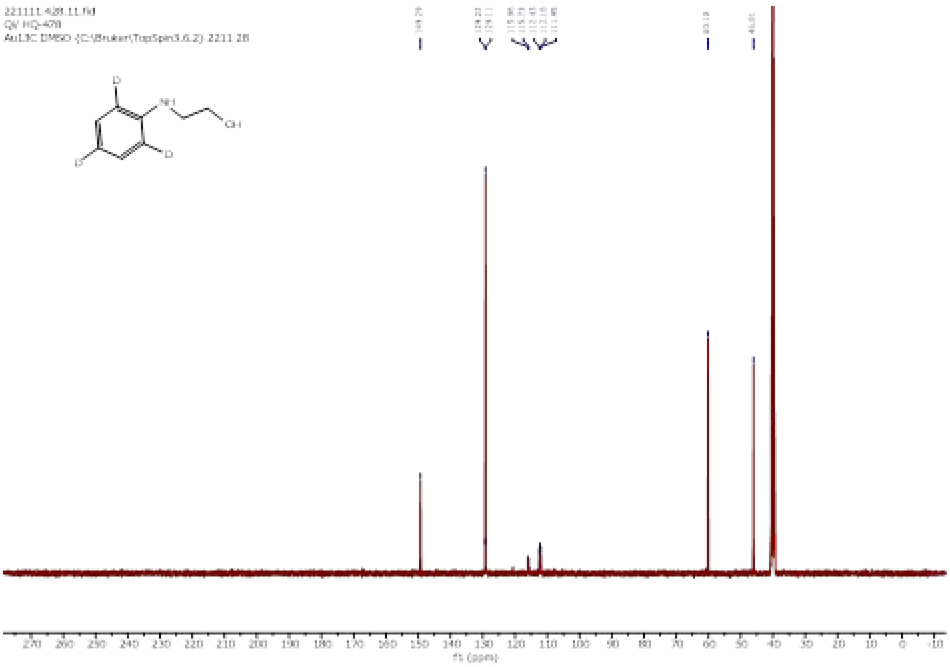

Figure S33. ^{1}H and ^{13}C NMR spectra of deuterated product **15b**.

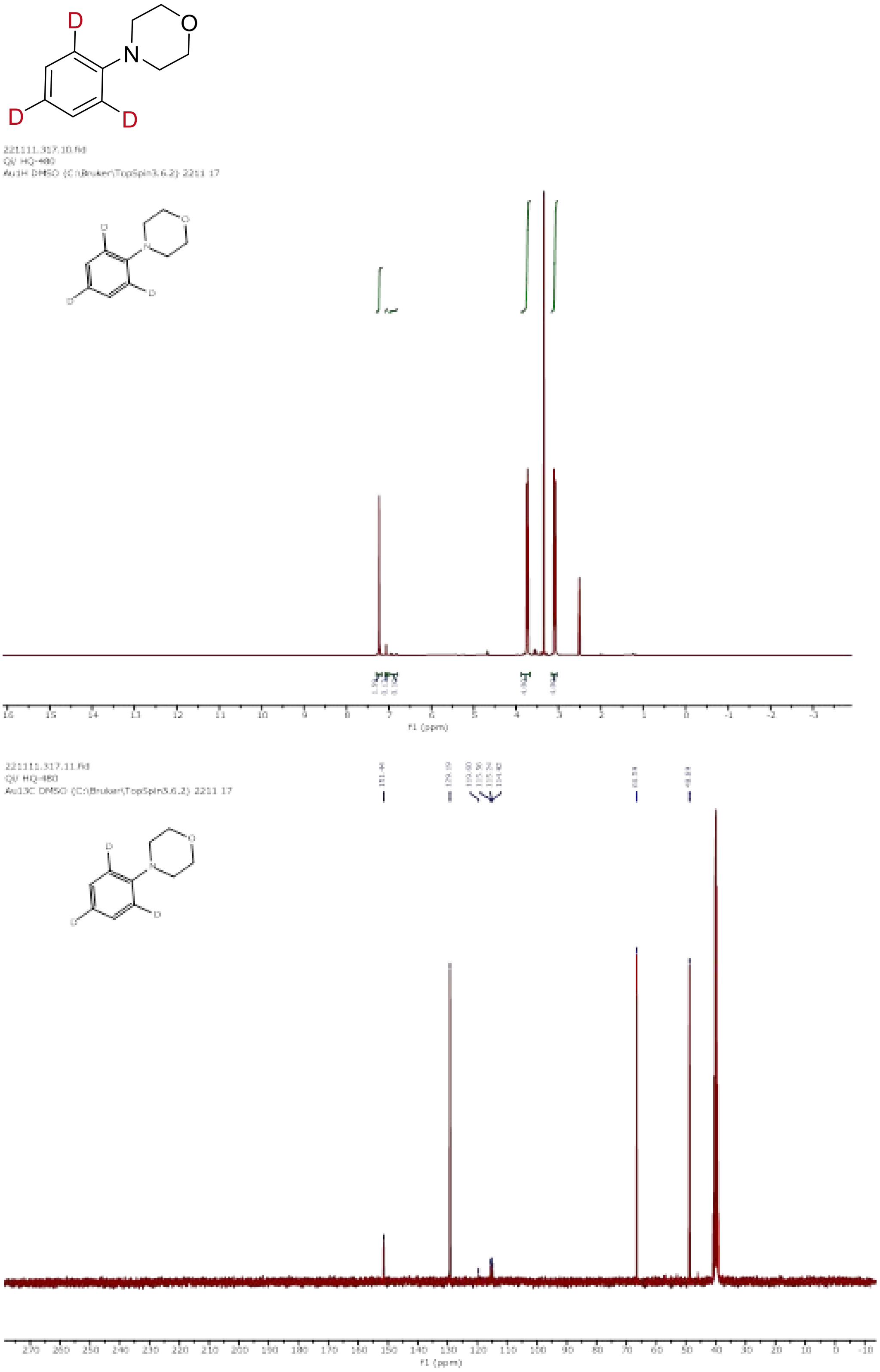

Figure S34. ^{1}H and ^{13}C NMR spectra of deuterated product **16b**.

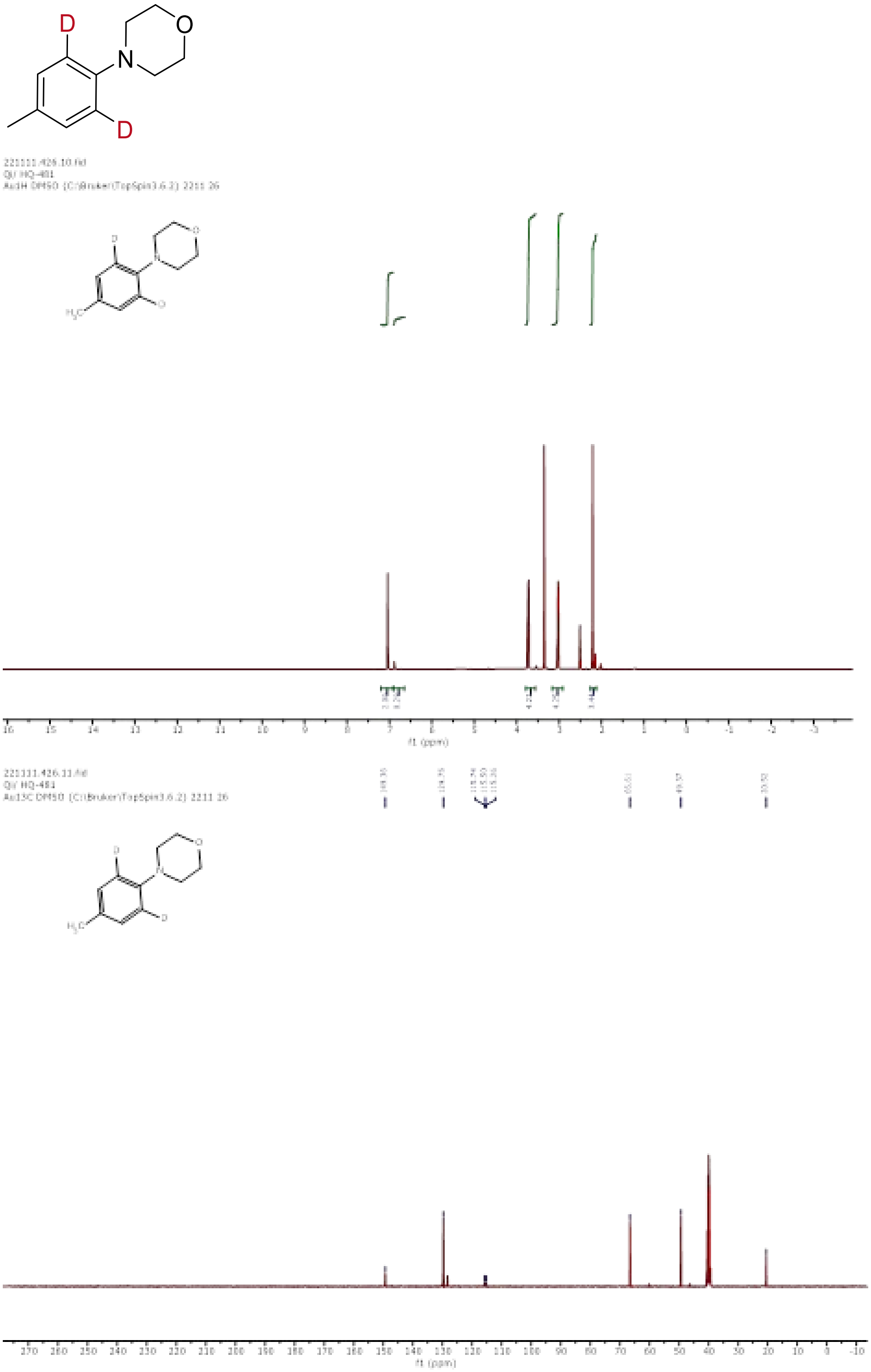

Figure S35. ^{1}H and ^{13}C NMR spectra of deuterated product **17b**.

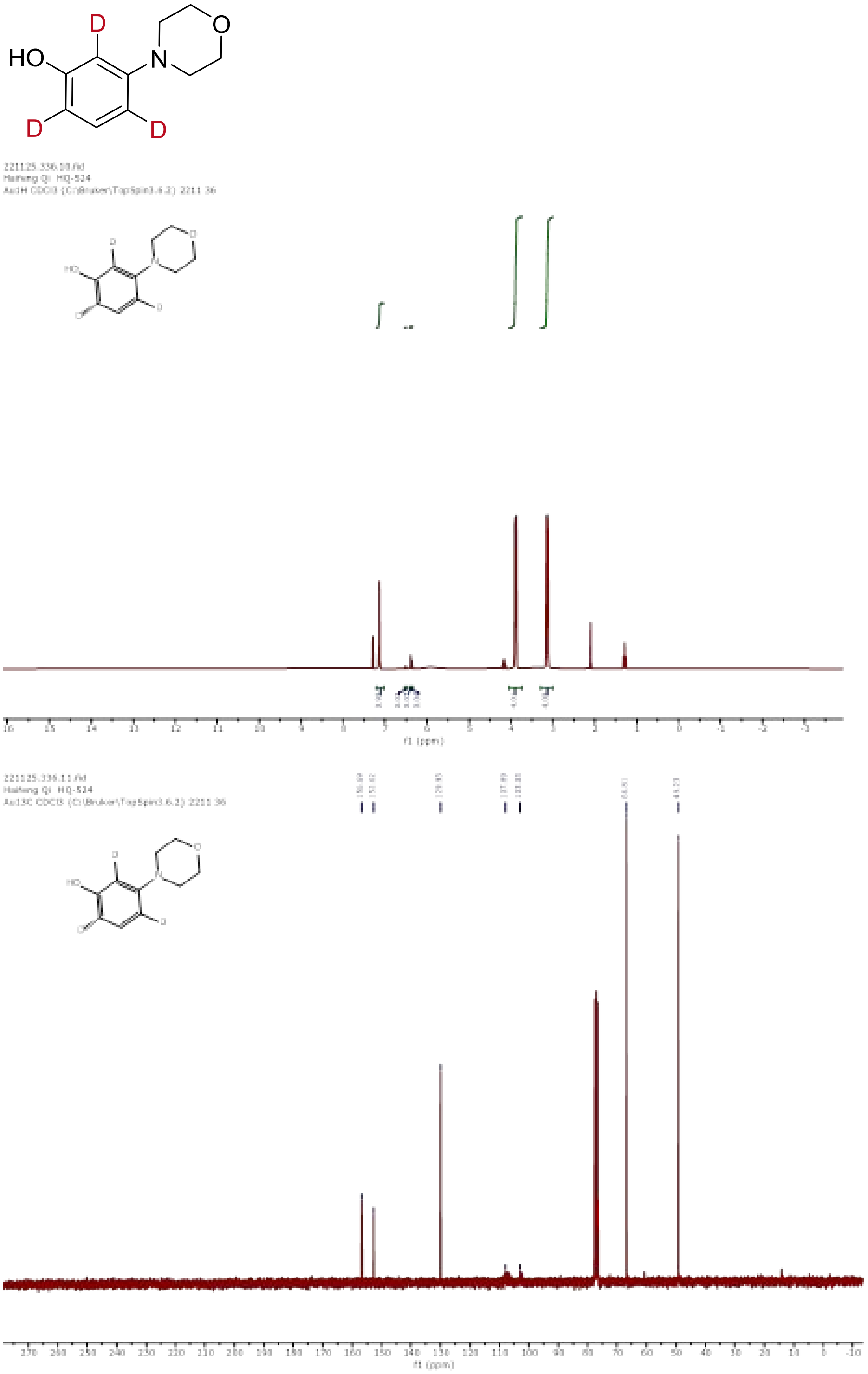

Figure S36. ^{1}H and ^{13}C NMR spectra of deuterated product **18b**.

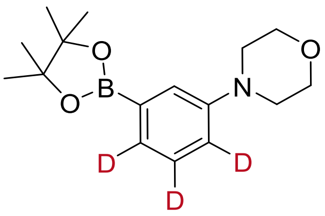

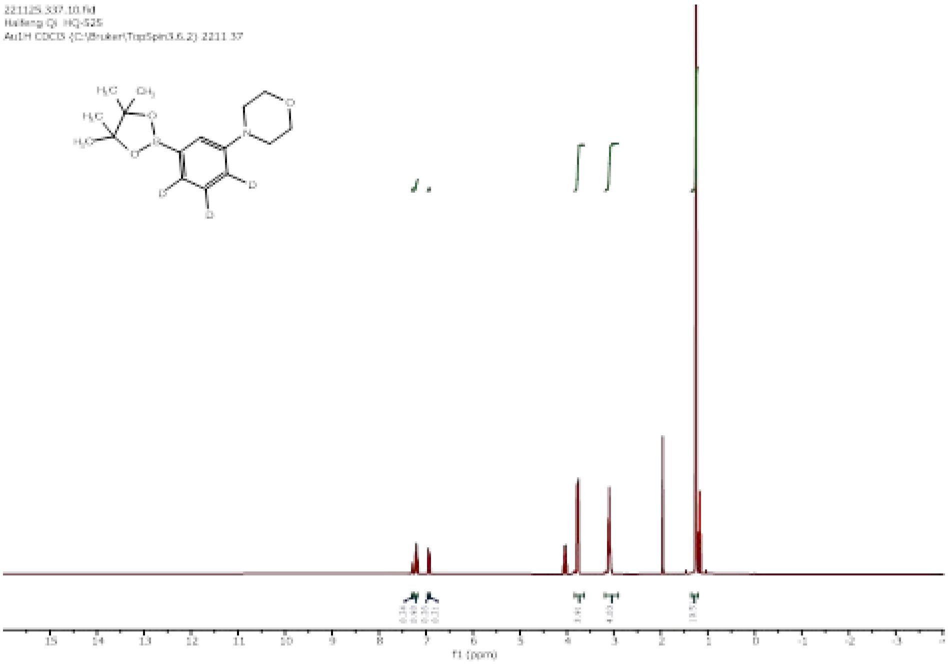

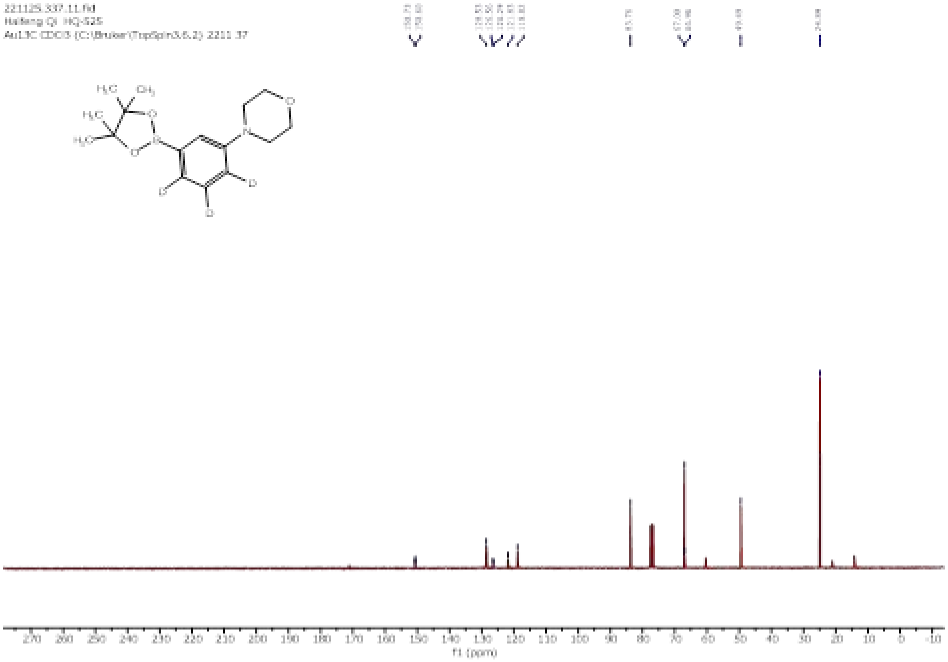

Figure S37. ^{1}H and ^{13}C NMR spectra of deuterated product **19b**.

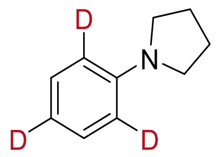

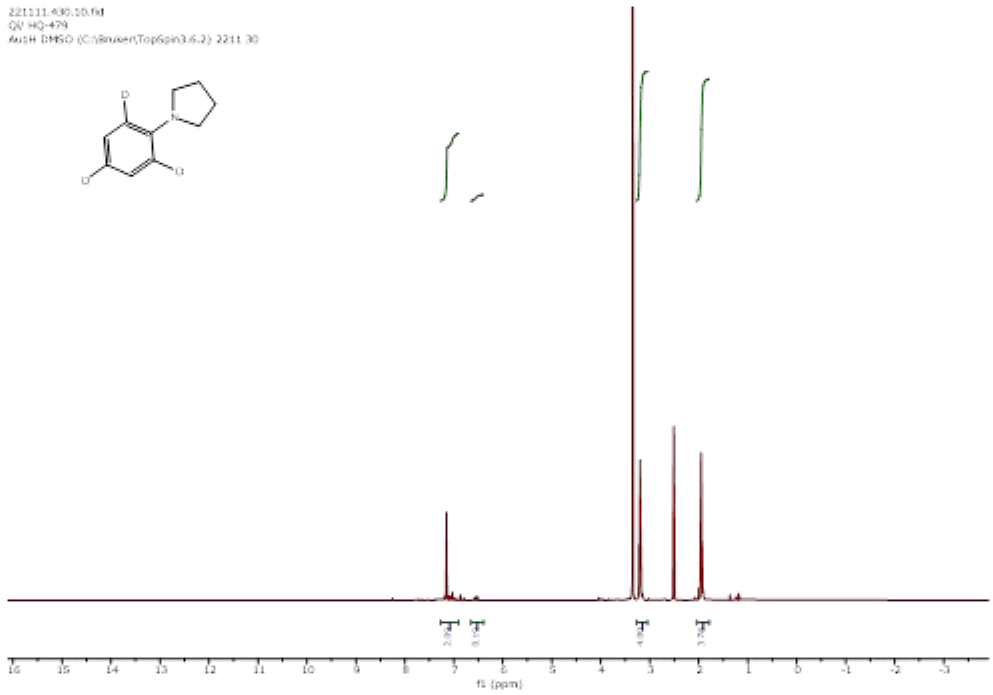

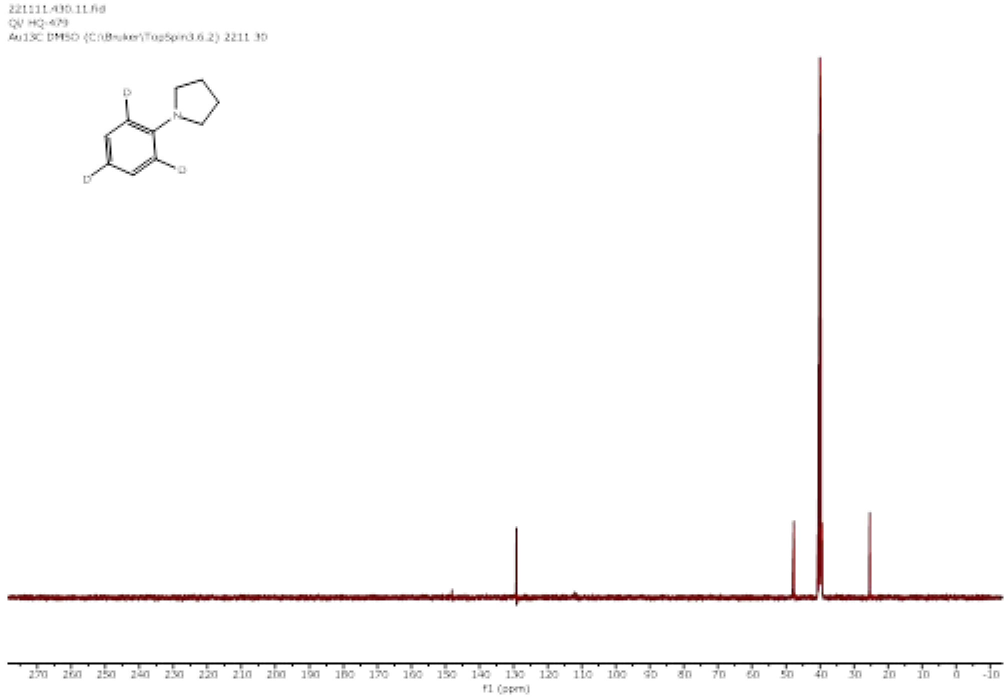

Figure S38. ^{1}H and ^{13}C NMR spectra of deuterated product **20b**.

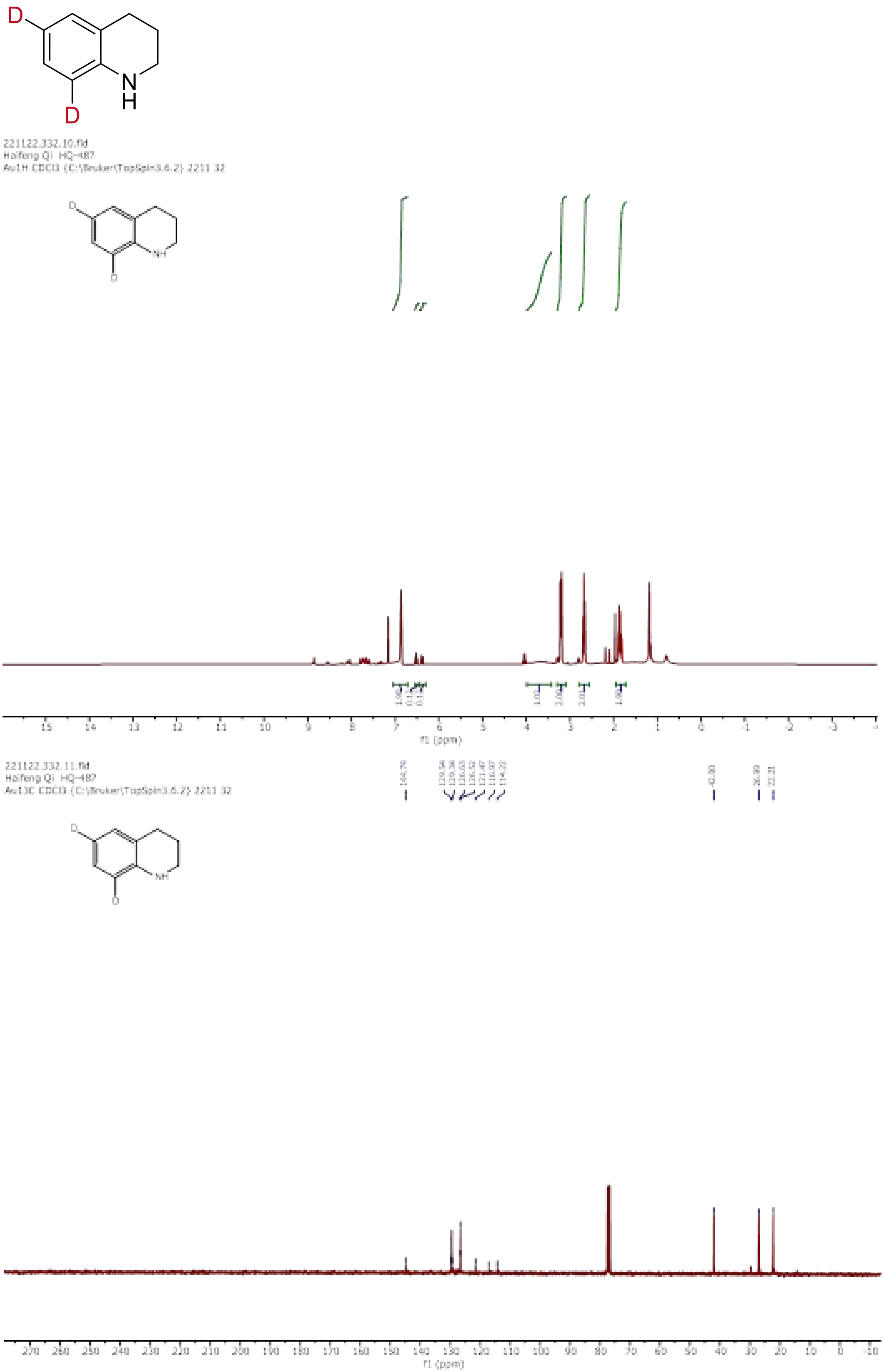

Figure S39. ^{1}H and ^{13}C NMR spectra of deuterated product **21b**.

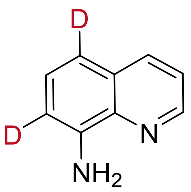

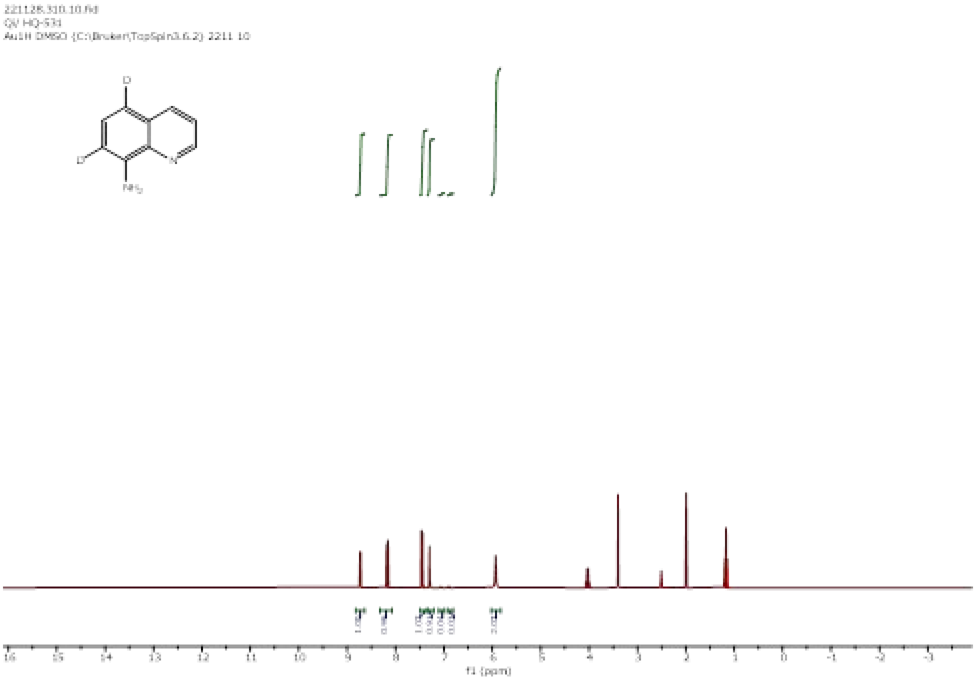

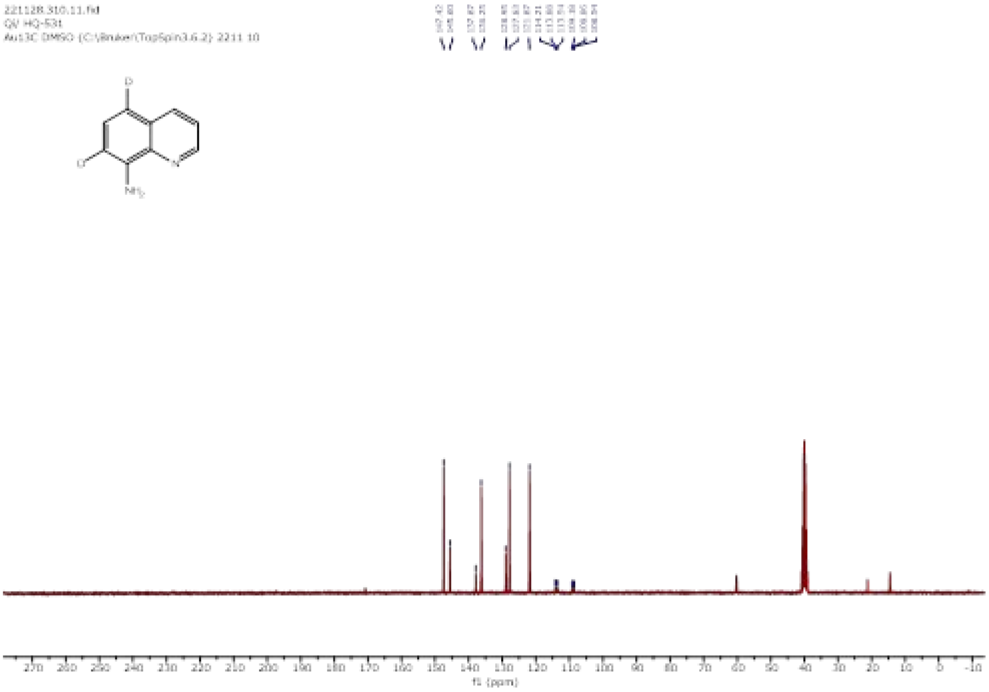

Figure S40. ^{1}H and ^{13}C NMR spectra of deuterated product **22b**.

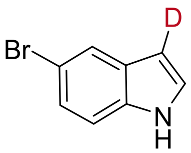

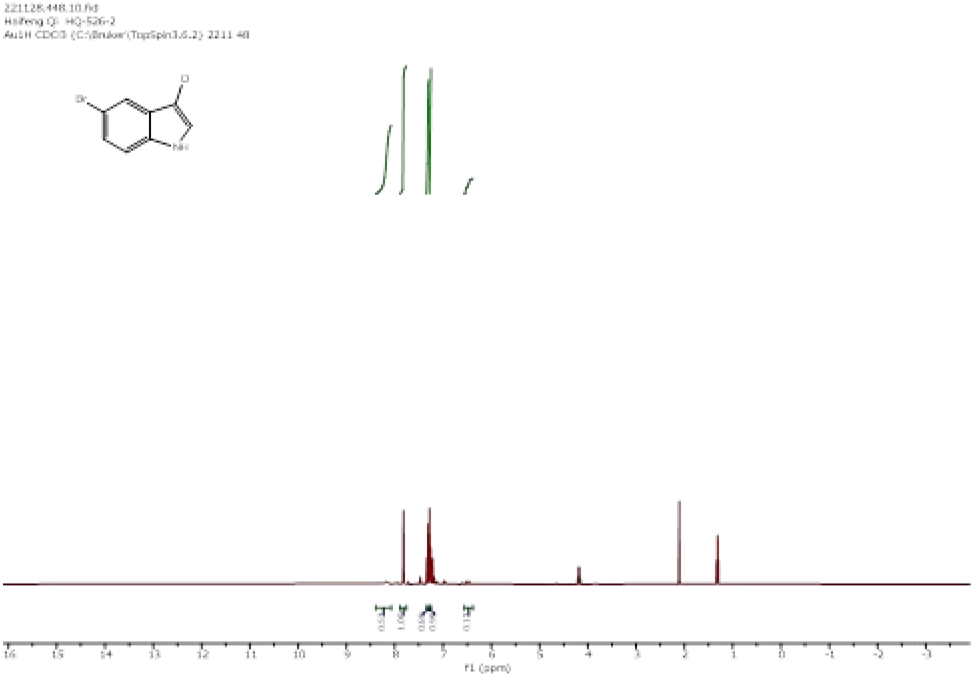

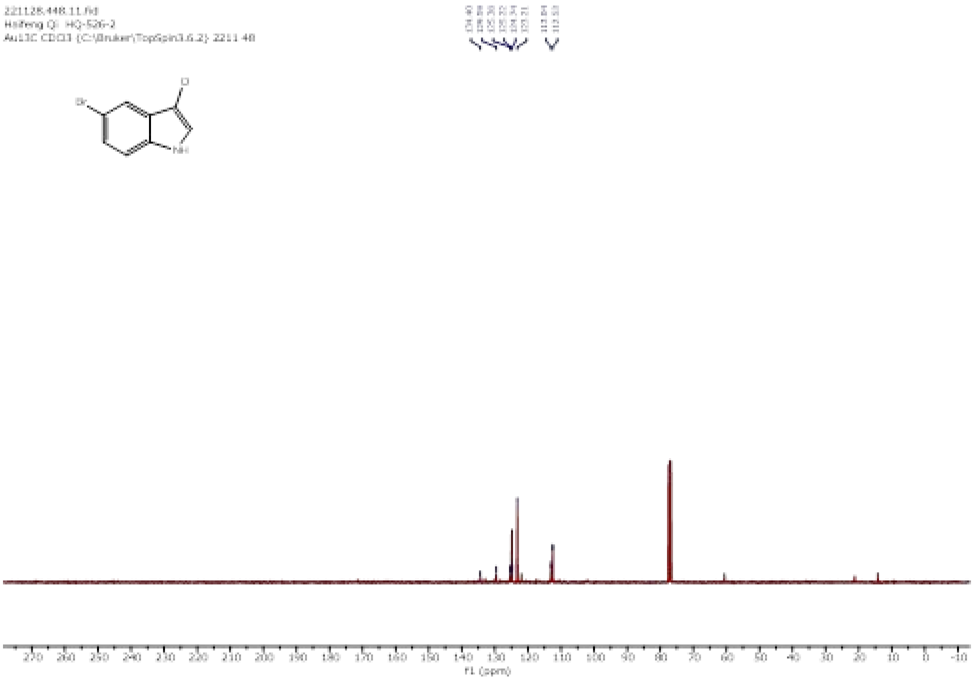

Figure S41. ^{1}H and ^{13}C NMR spectra of deuterated product **23b**.

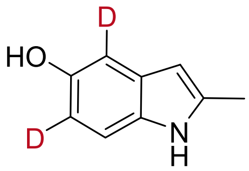

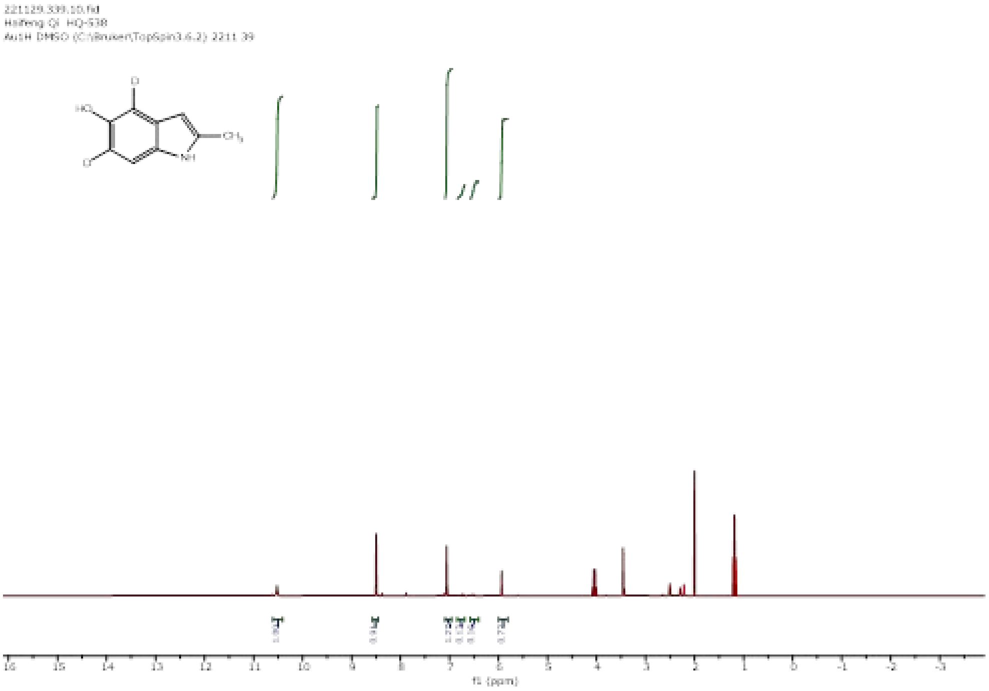

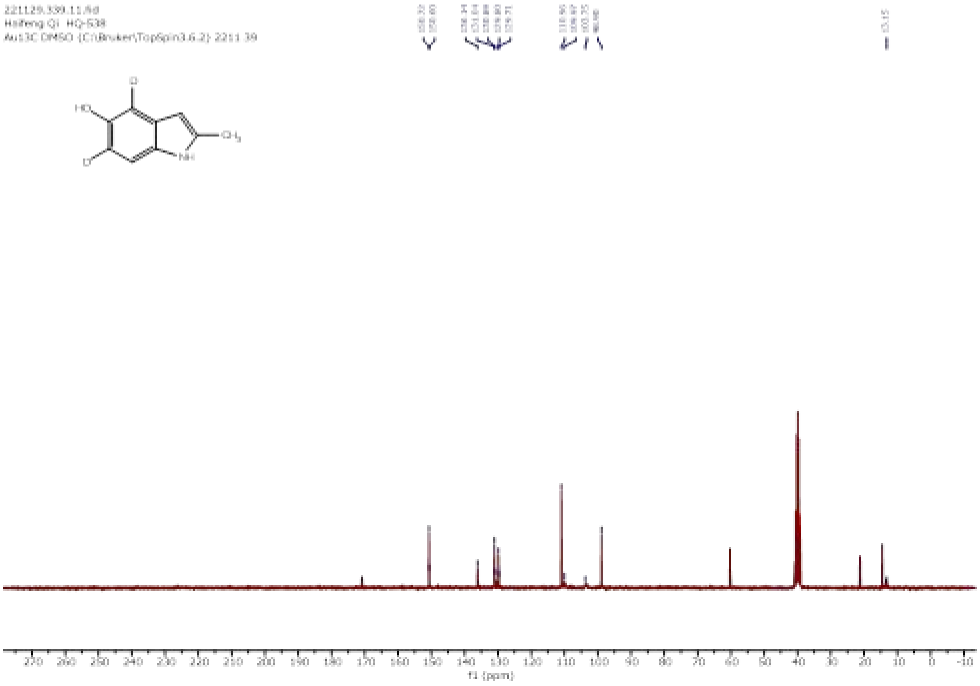

Figure S42. ^{1}H and ^{13}C NMR spectra of deuterated product **24b**.

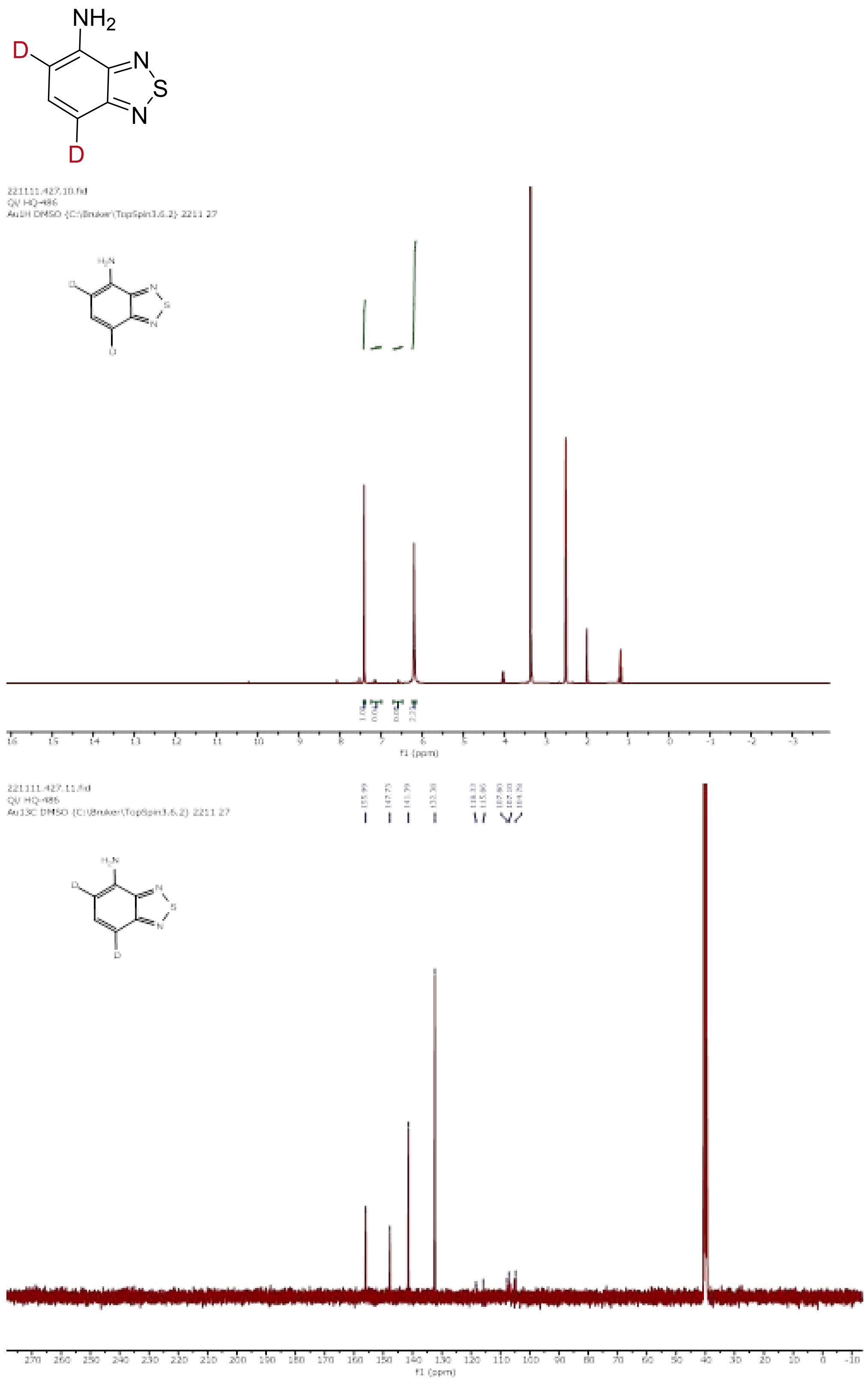

Figure S43. ^{1}H and ^{13}C NMR spectra of deuterated product **25b**.

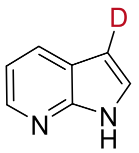

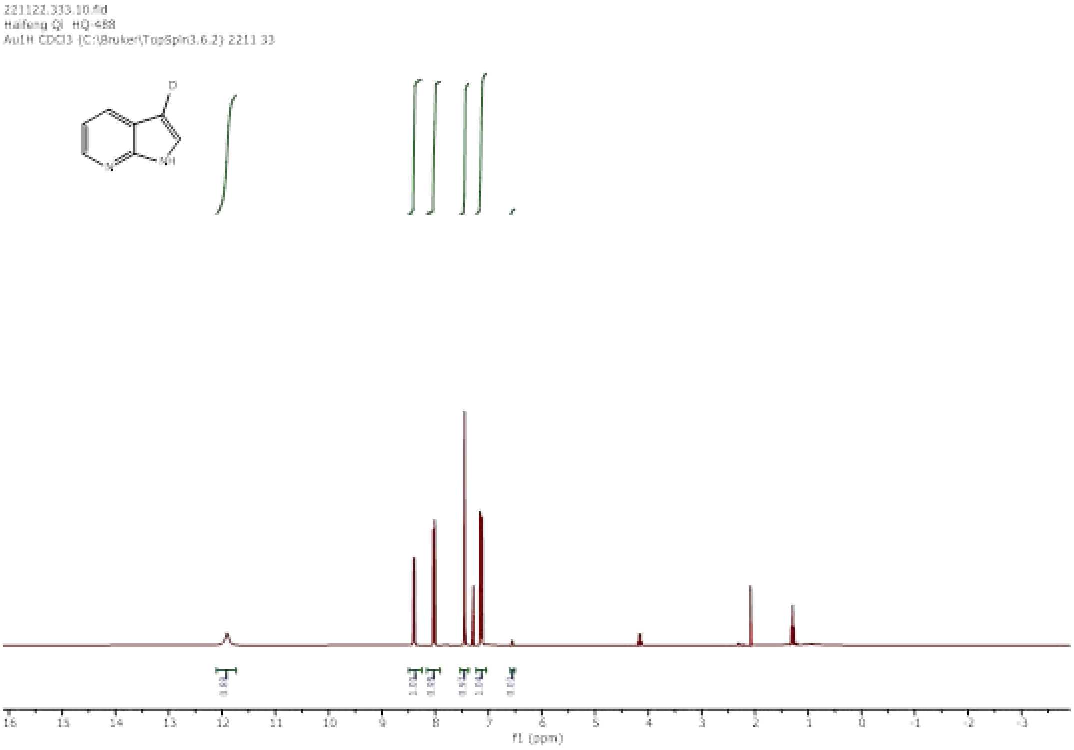

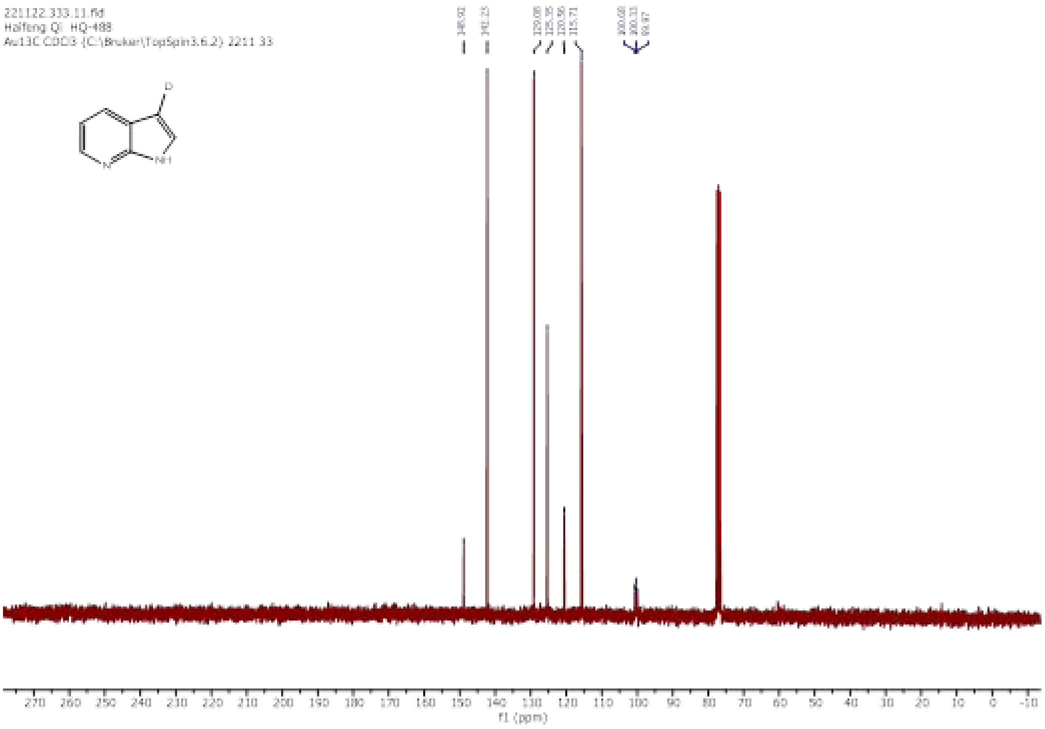

Figure S44. ^{1}H and ^{13}C NMR spectra of deuterated product **26b**.

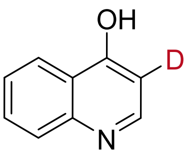

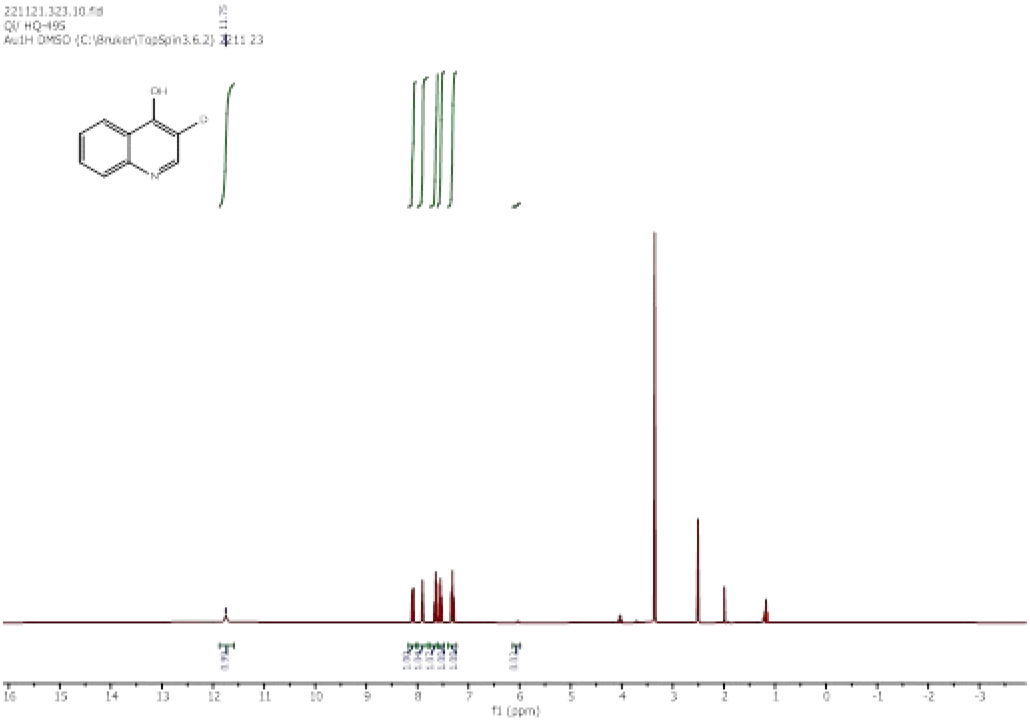

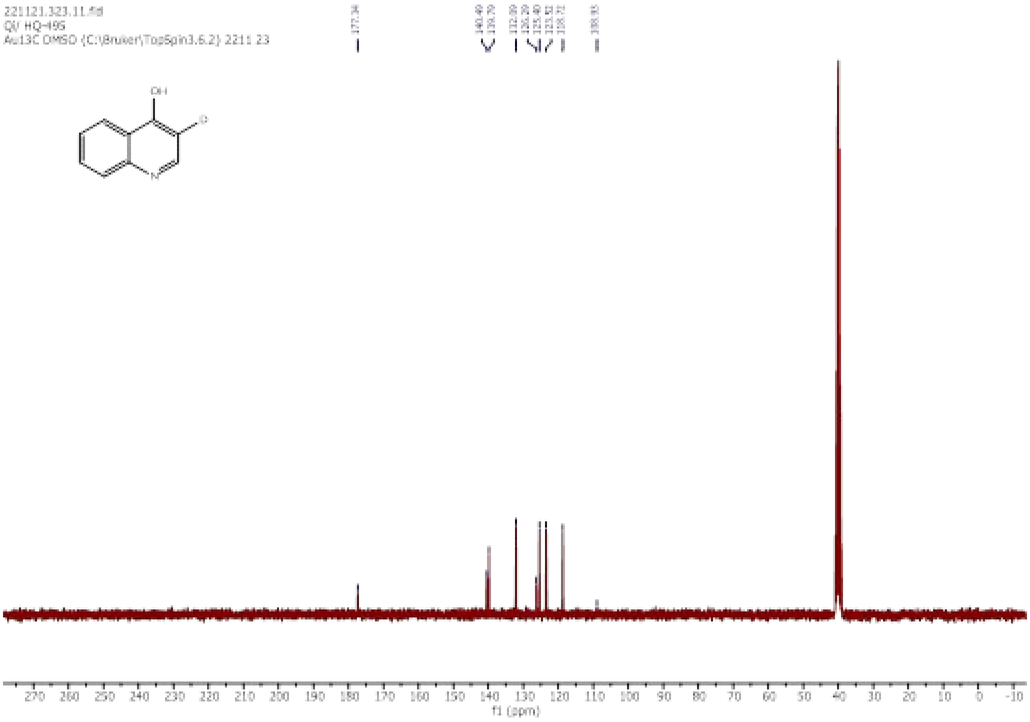

Figure S45. ^{1}H and ^{13}C NMR spectra of deuterated product **27b**.

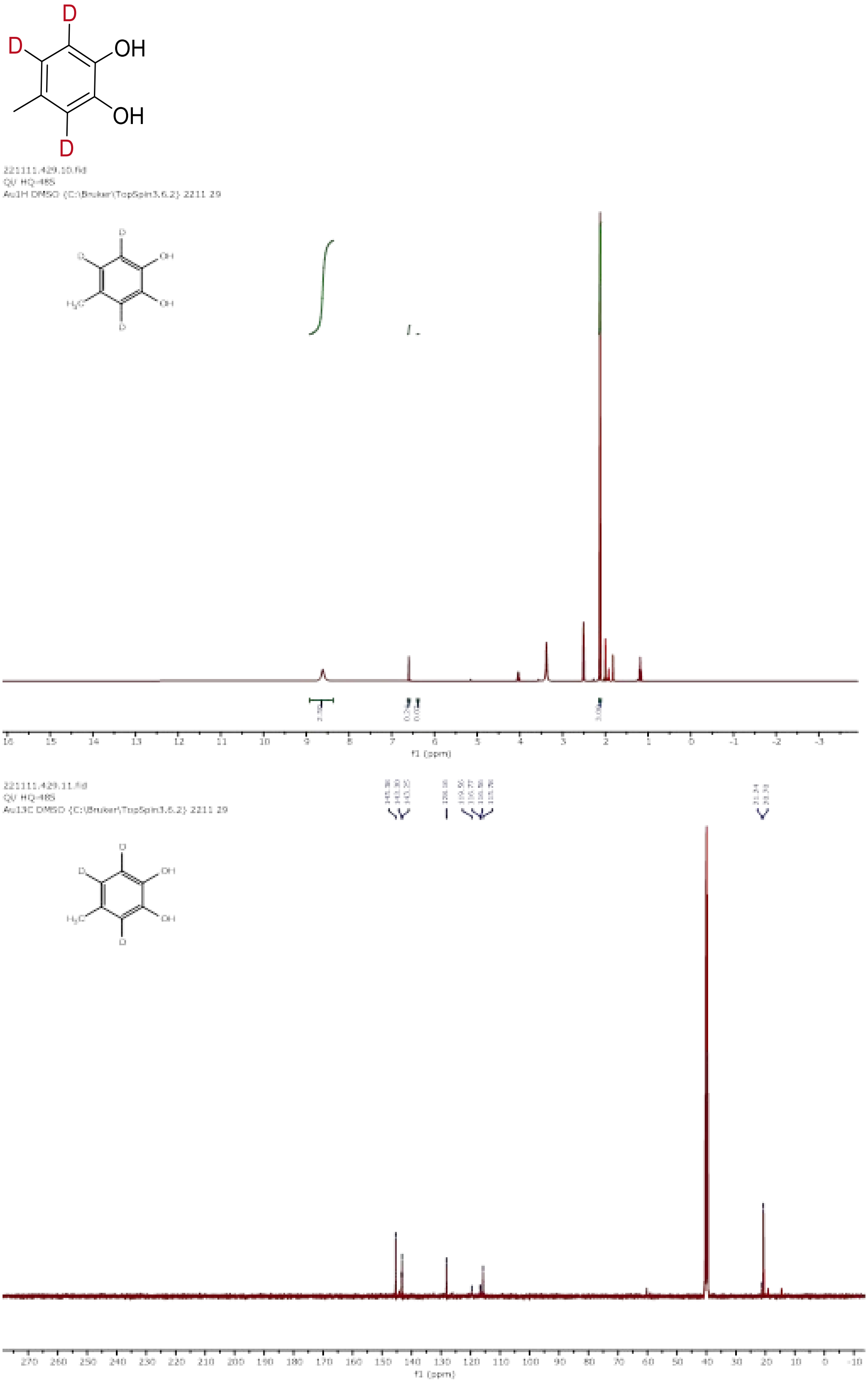

Figure S46. ^{1}H and ^{13}C NMR spectra of deuterated product **28b**.

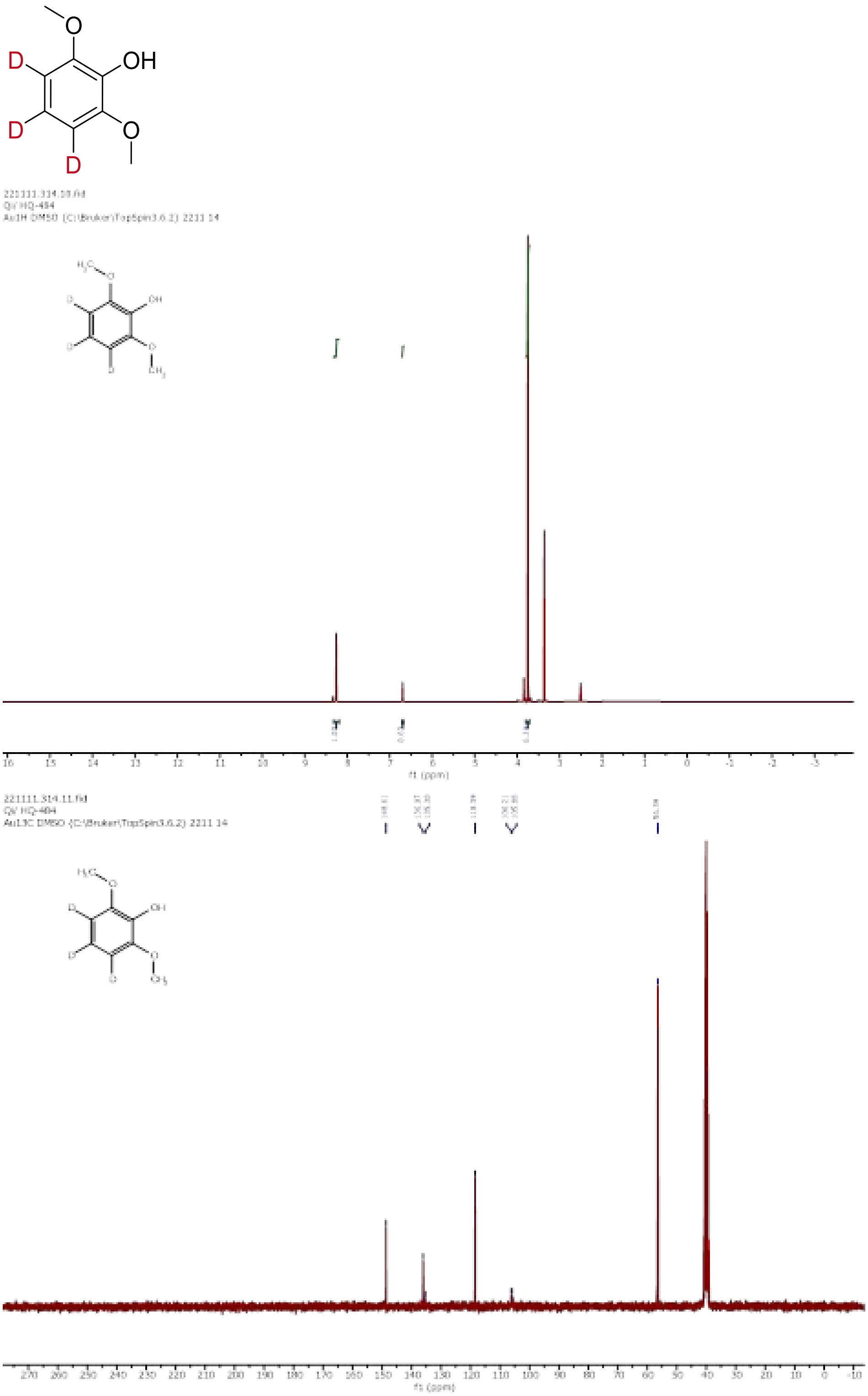

Figure S47. ^{1}H and ^{13}C NMR spectra of deuterated product **29b**.

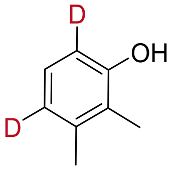

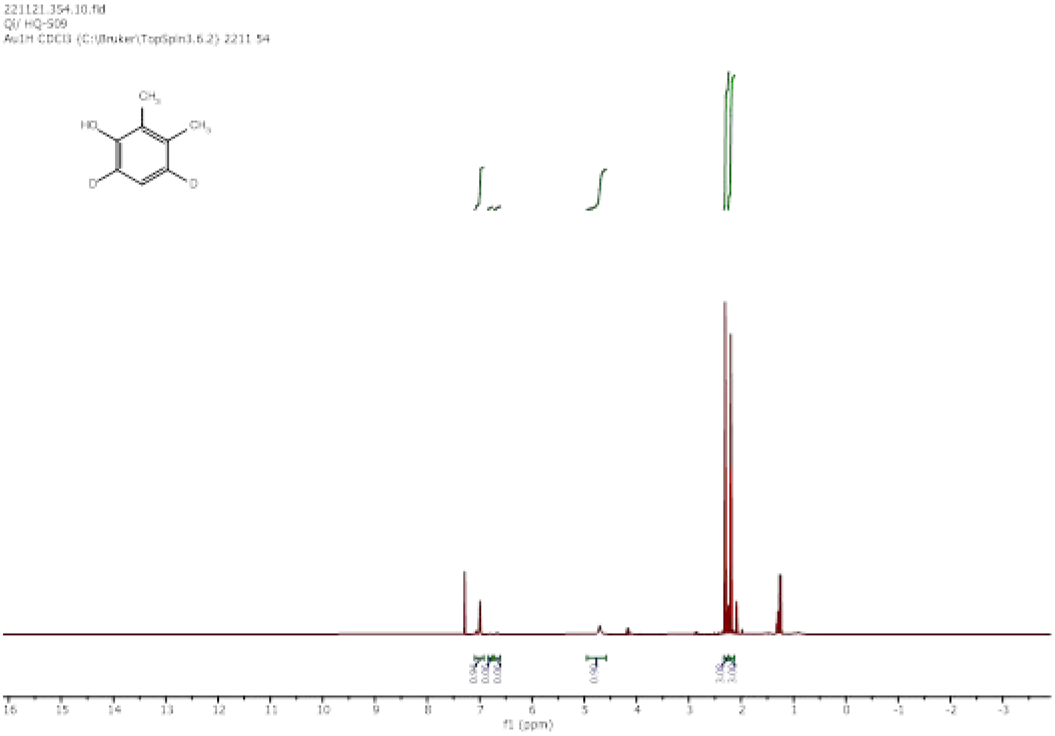

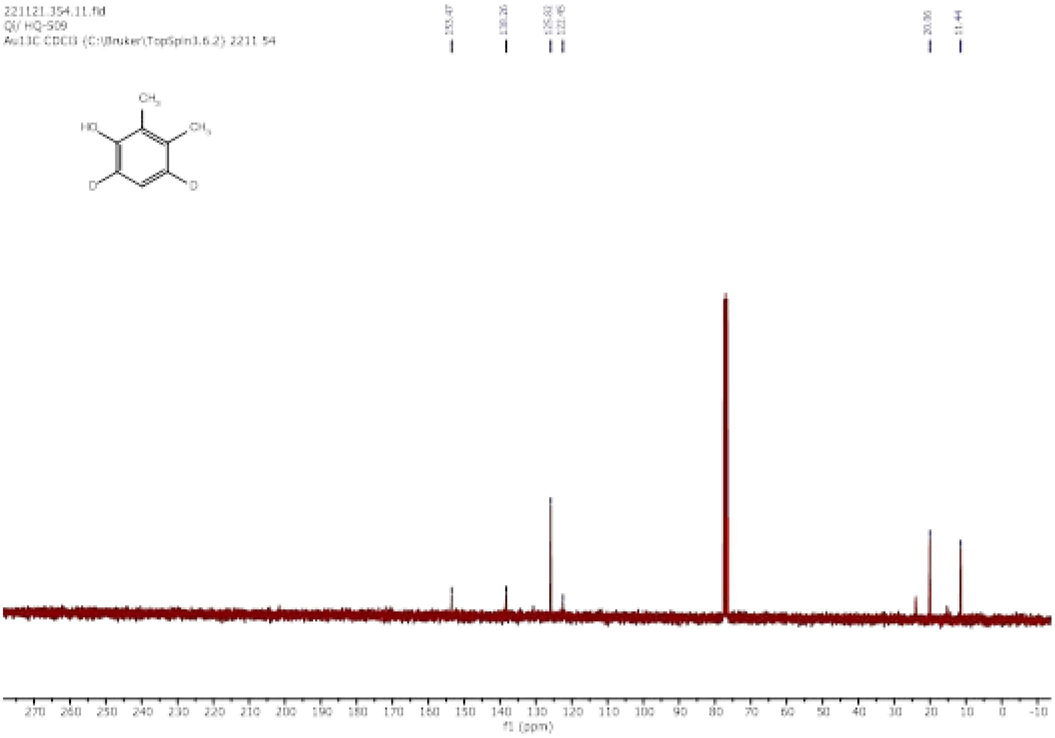

Figure S48. ^{1}H and ^{13}C NMR spectra of deuterated product **30b**.

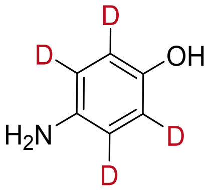

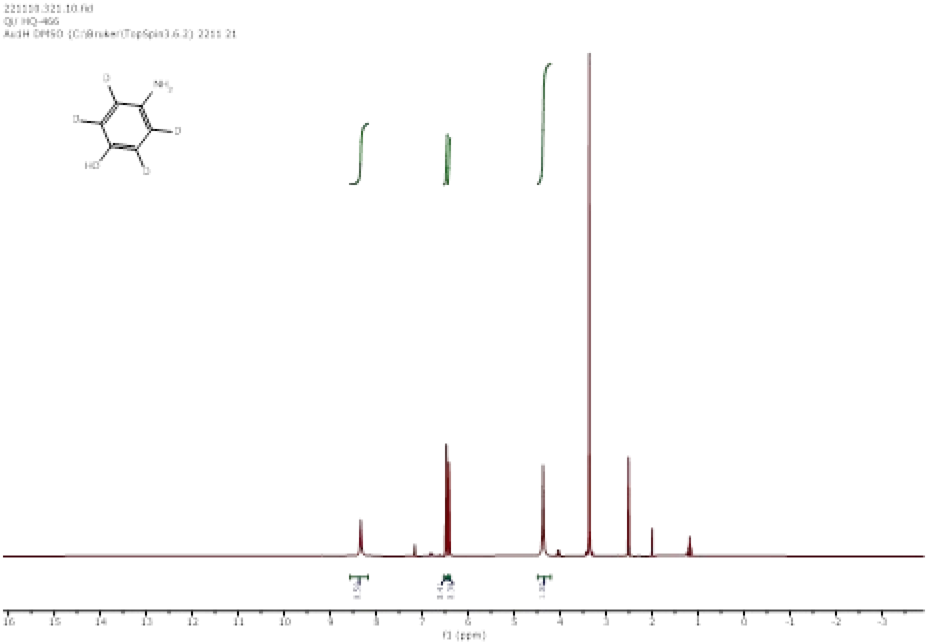

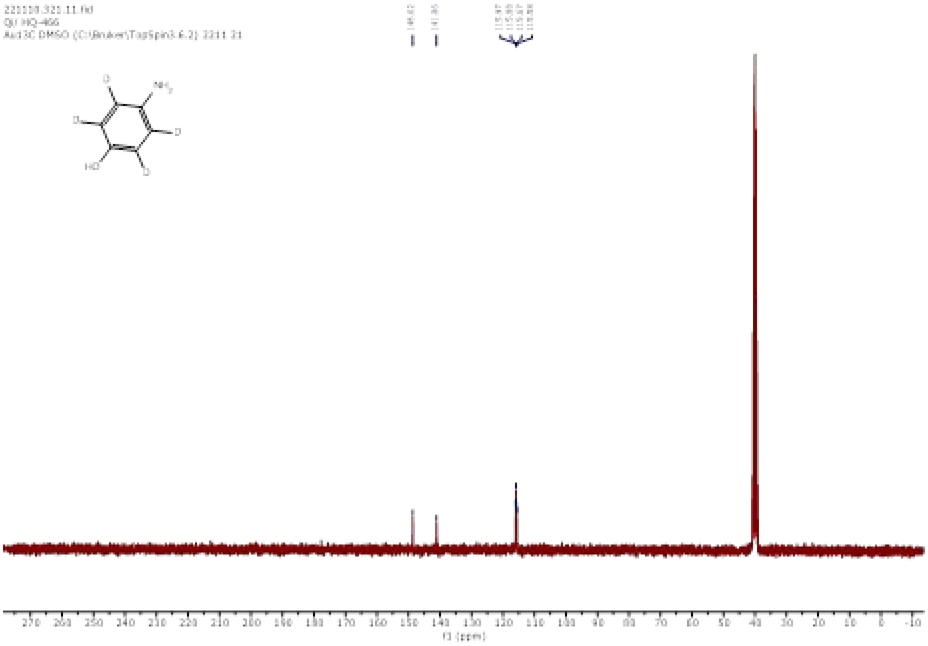

Figure S49. ^{1}H and ^{13}C NMR spectra of deuterated product **31b**.

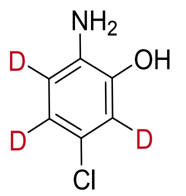

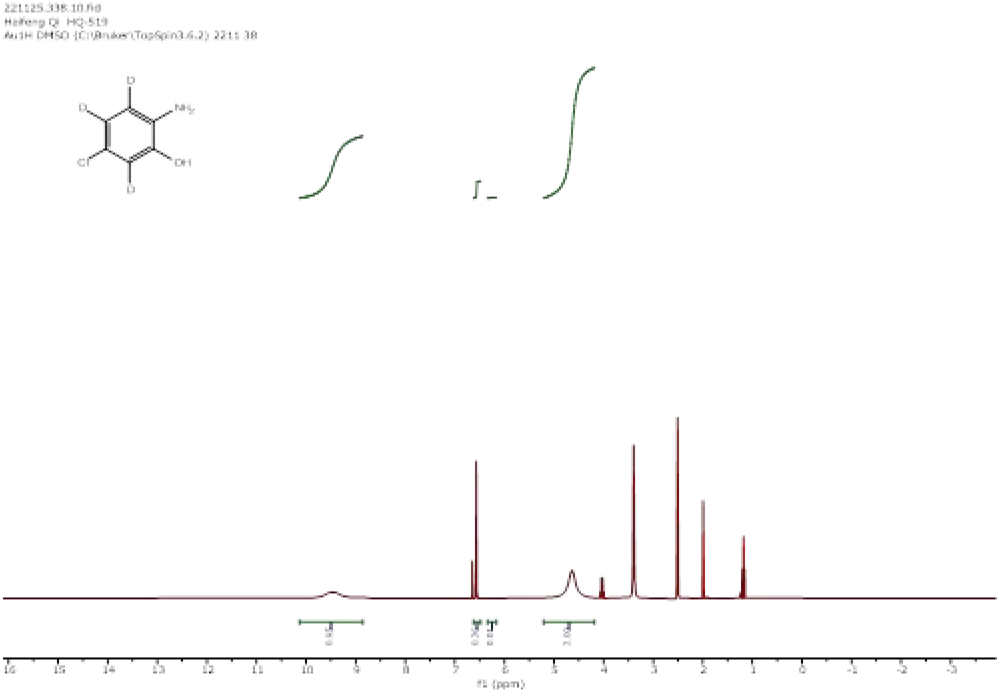

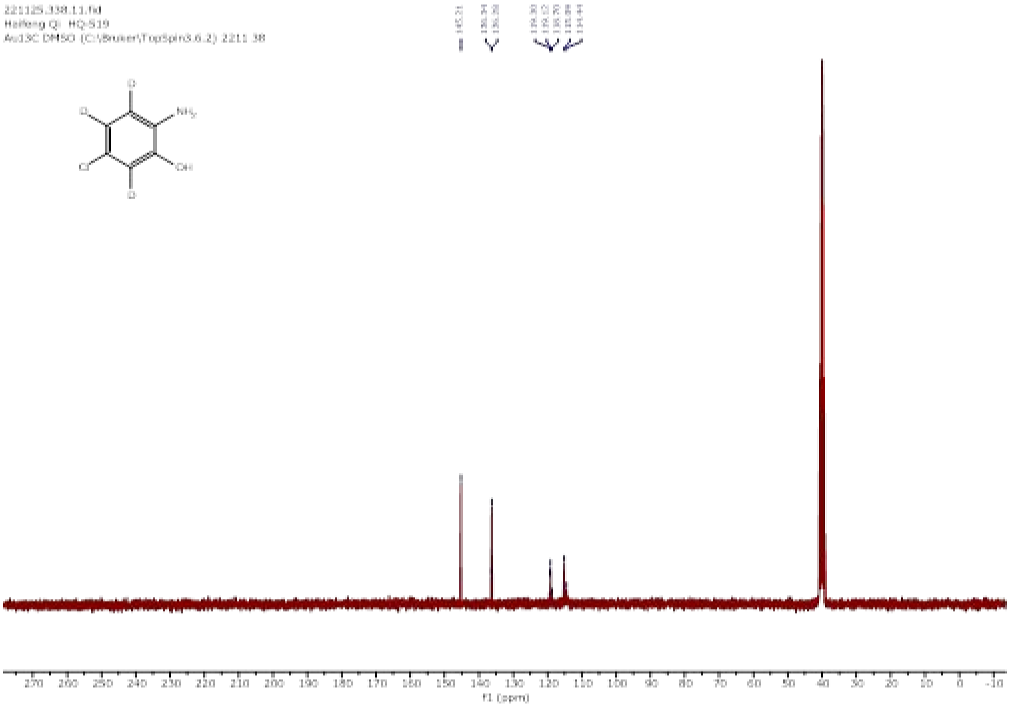

Figure S50. ^{1}H and ^{13}C NMR spectra of deuterated product **32b**.

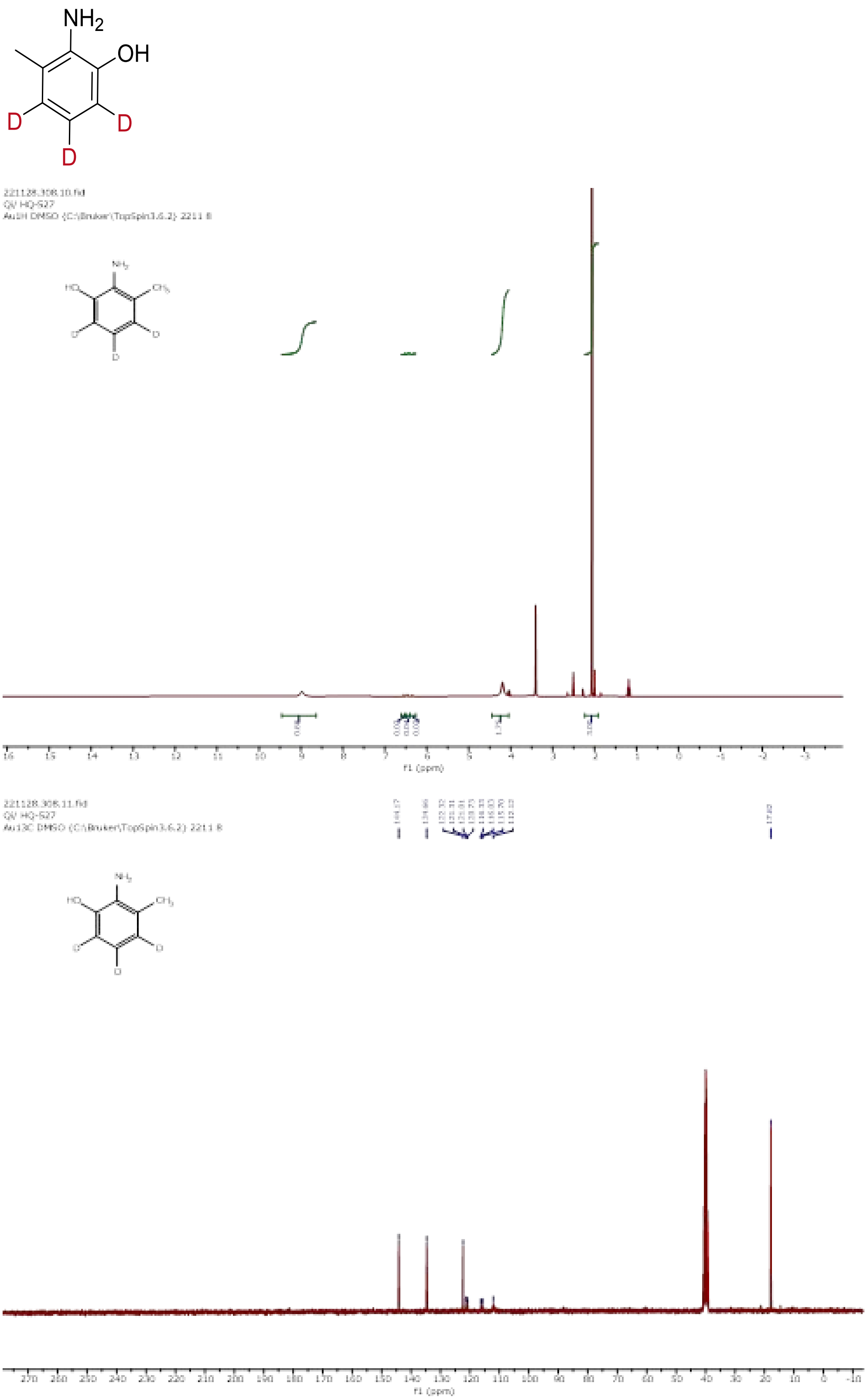

Figure S51. ^{1}H and ^{13}C NMR spectra of deuterated product **33b**.

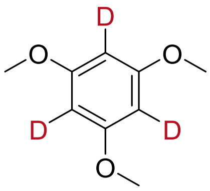

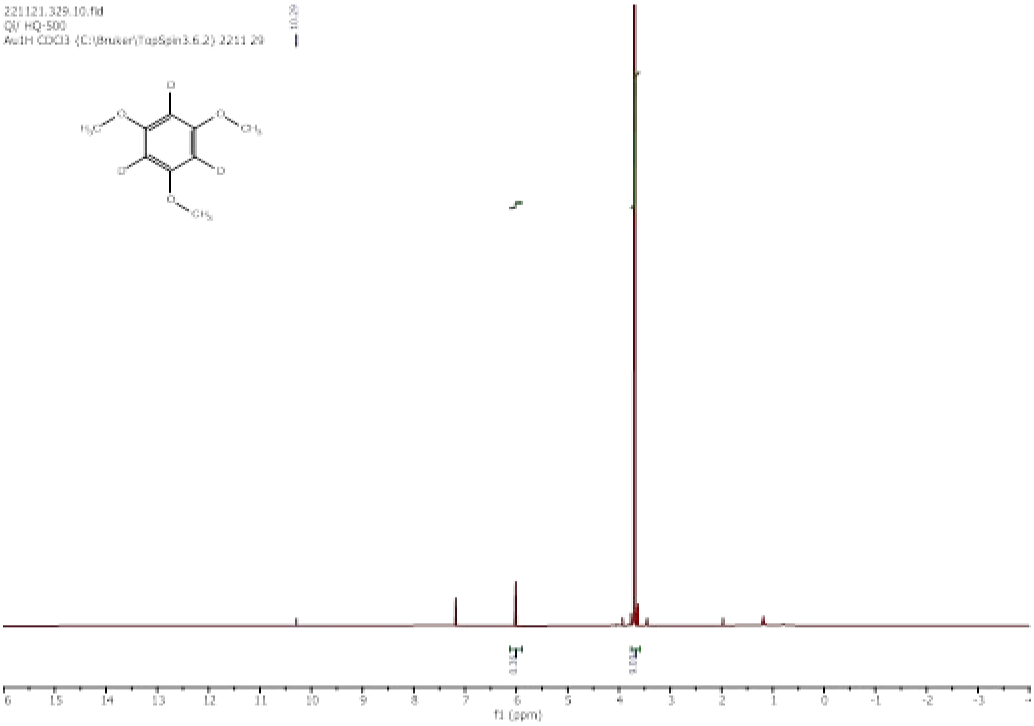

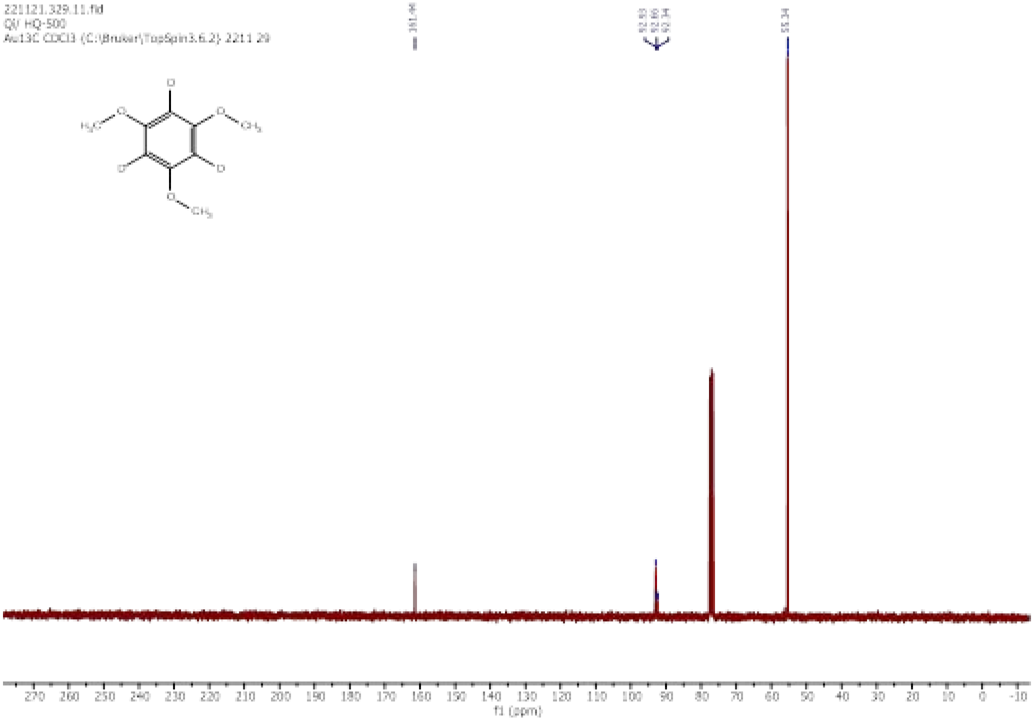

Figure S52. ^{1}H and ^{13}C NMR spectra of deuterated product **34b**.

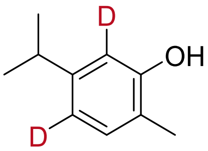

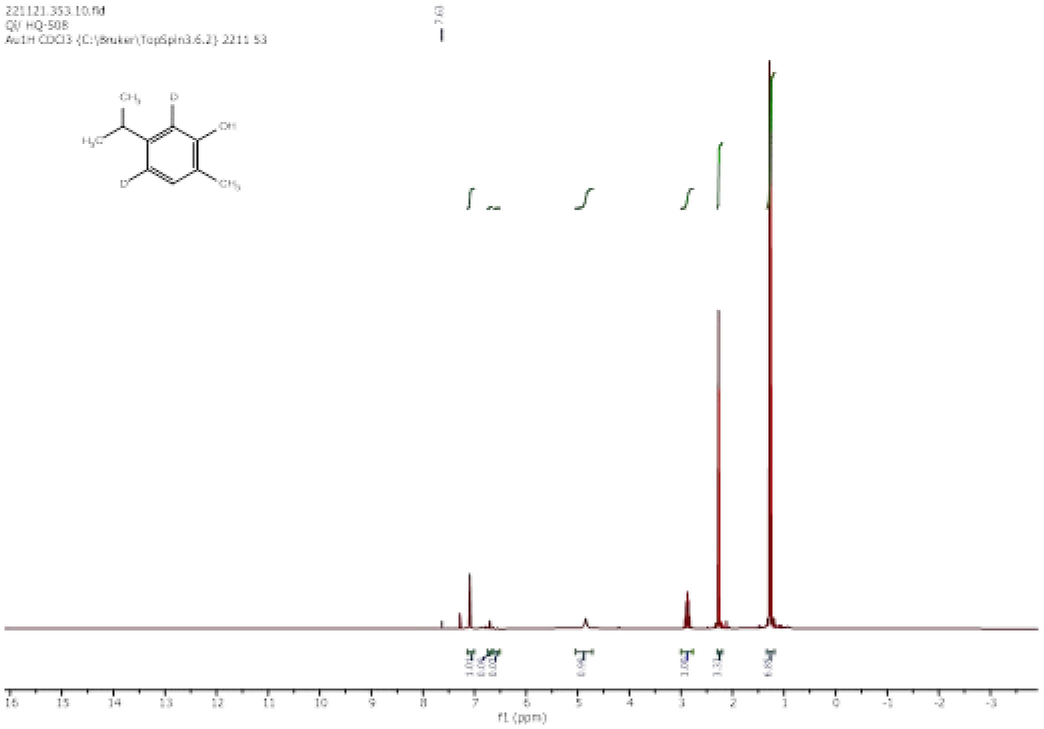

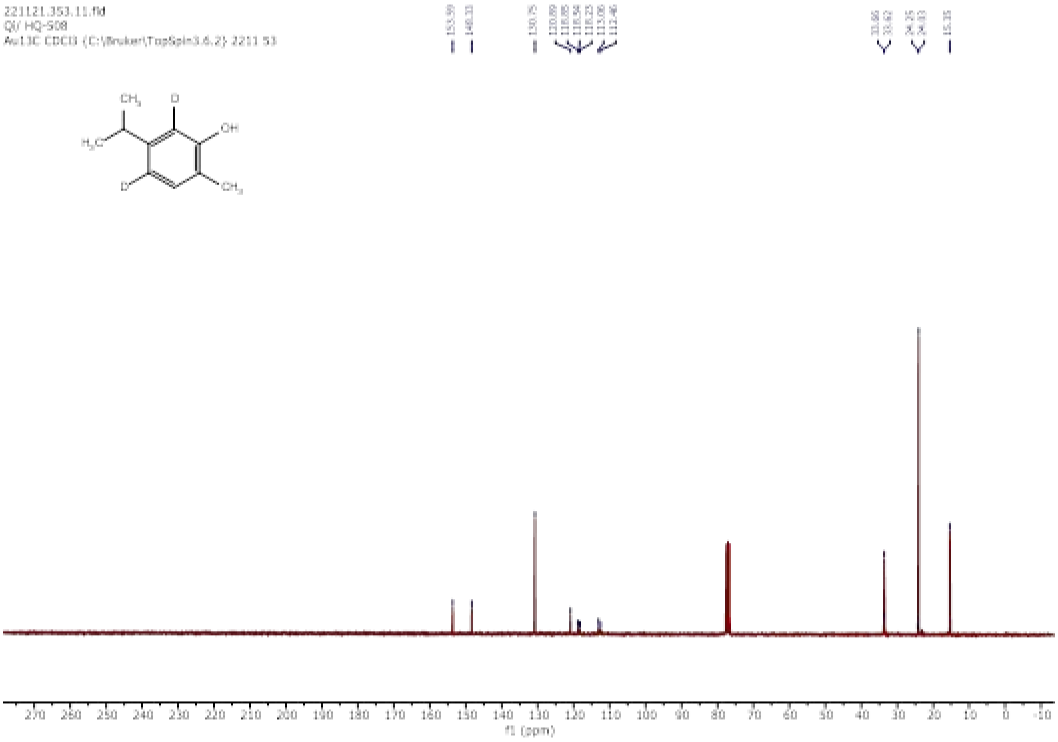

Figure S53. ^{1}H and ^{13}C NMR spectra of deuterated product **35b**.

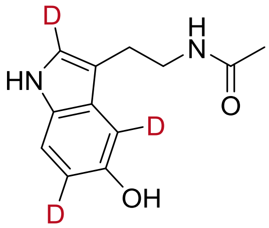

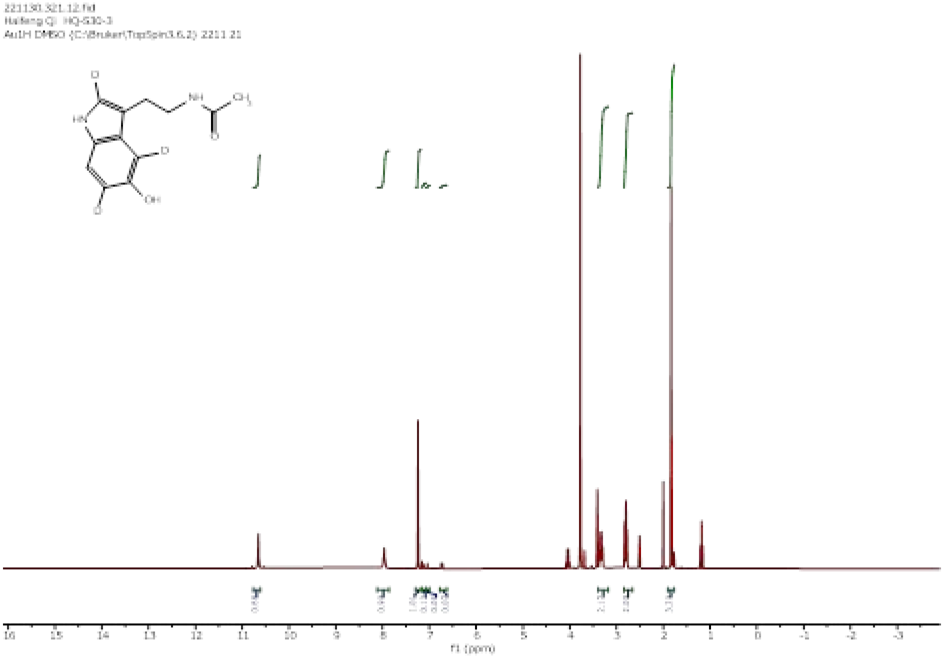

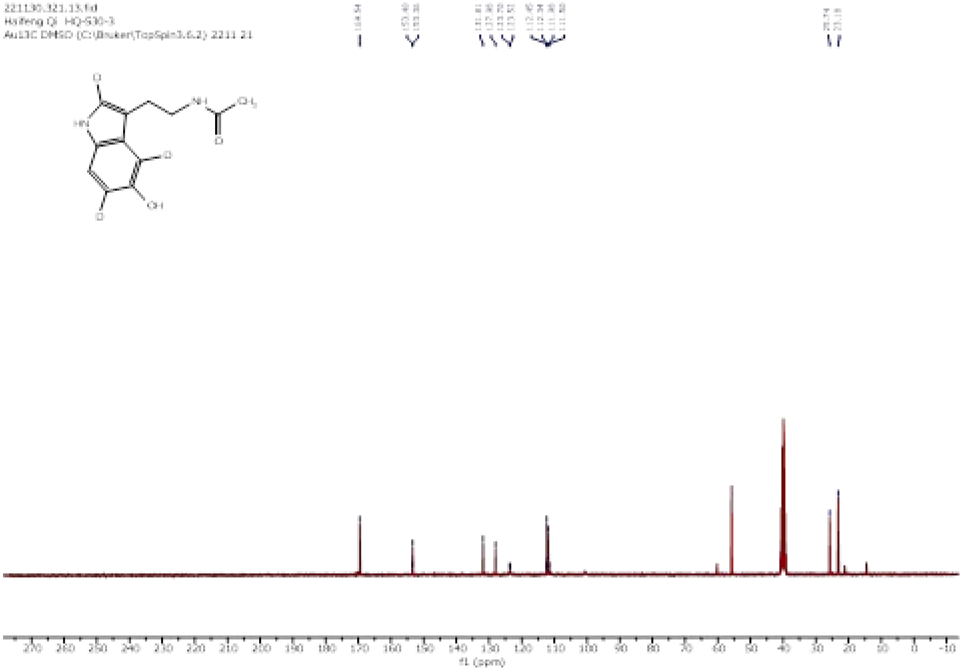

Figure S54. ^{1}H and ^{13}C NMR spectra of deuterated product **36b**.

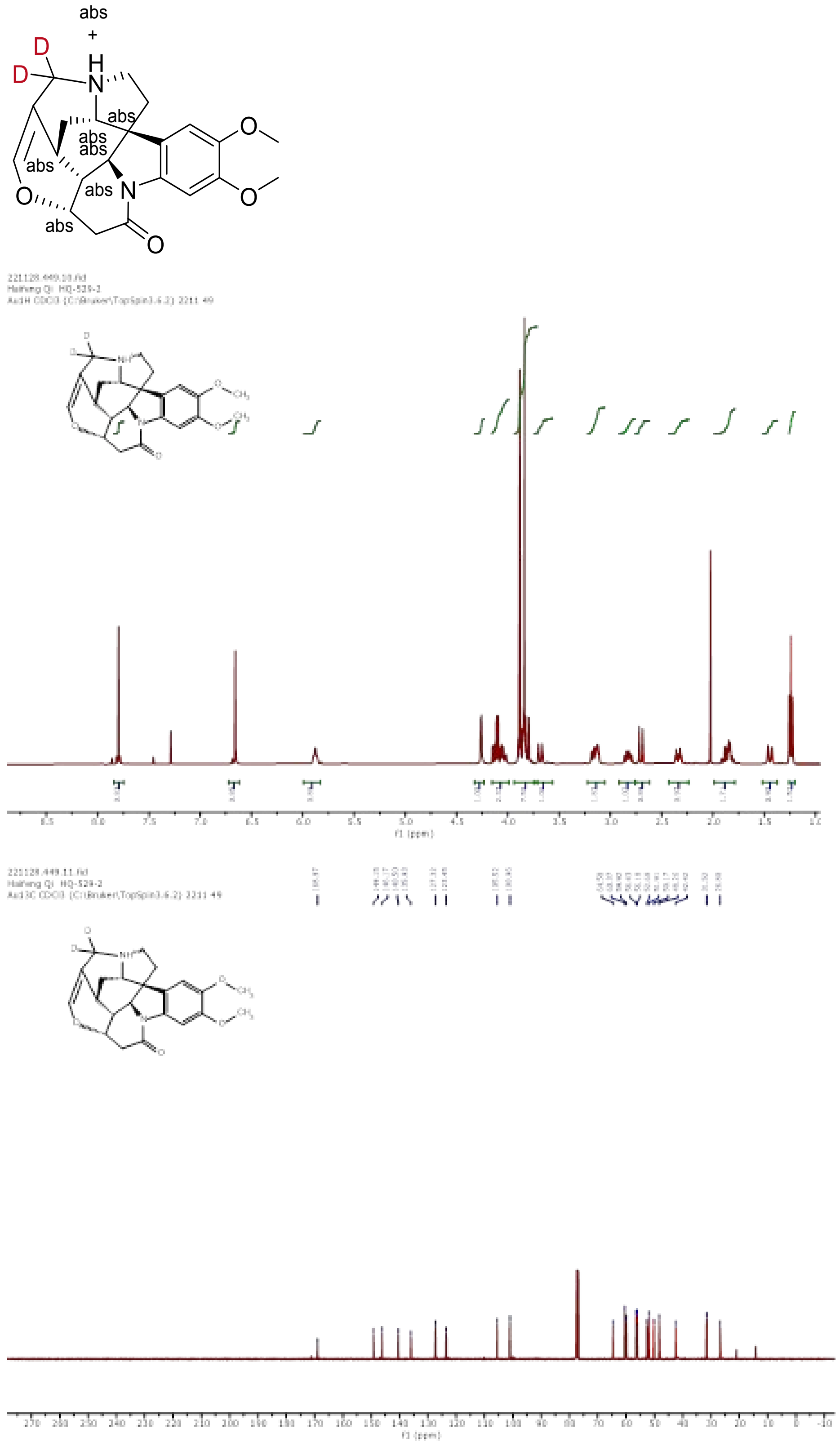

Figure S55. ^{1}H and ^{13}C NMR spectra of deuterated product **37b**.

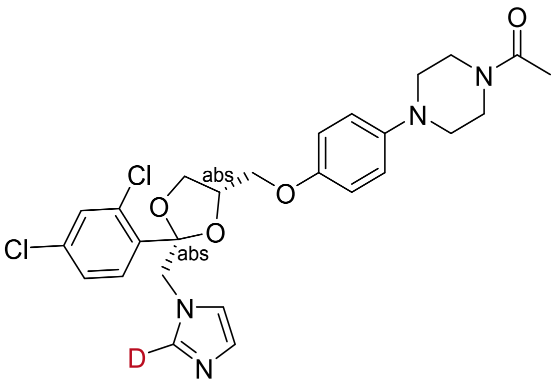

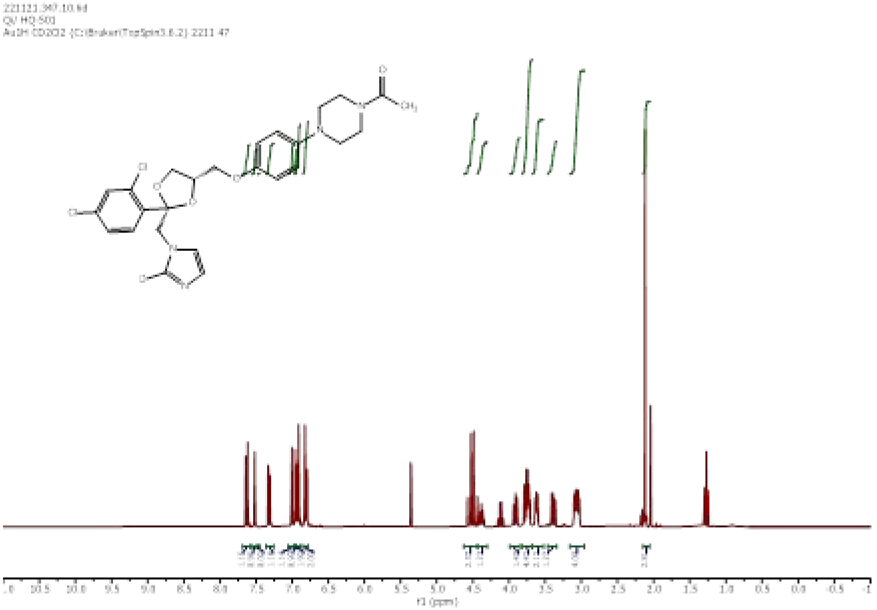

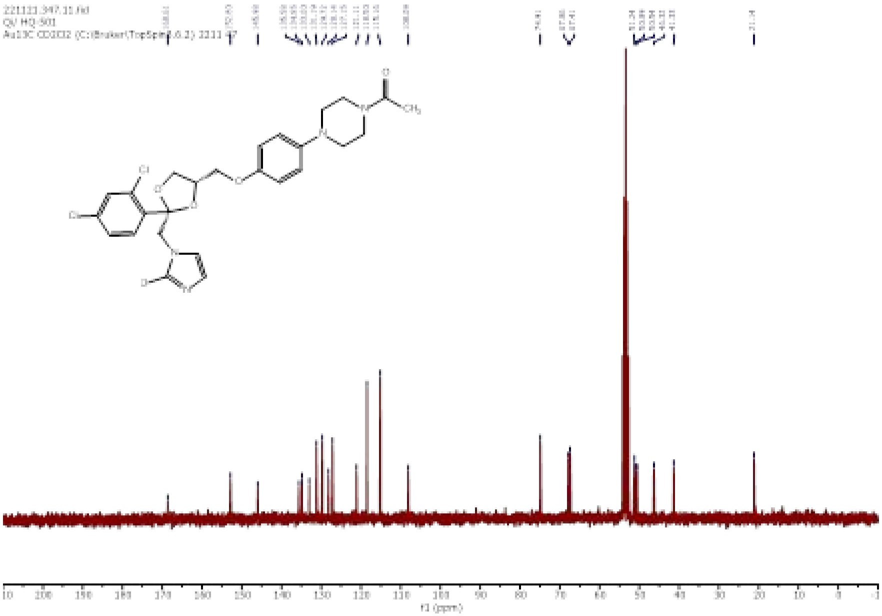

Figure S56. ^{1}H and ^{13}C NMR spectra of deuterated product **38b**.
